# Analysis of the Milk Oligosaccharides Spectrum and Sialylation Status of Milk from West African Dwarf Goat and East Friesian Sheep

**DOI:** 10.1021/acsomega.5c13396

**Published:** 2026-05-27

**Authors:** Lisa Isernhagen, Christina E. Galuska, Andreas Hoeflich, Sebastian P. Galuska

**Affiliations:** 28415 Research Institute for Farm Animal Biology (FBN), Wilhelm-Stahl-Allee 2, Dummerstorf 18196, Germany

## Abstract

Milk oligosaccharides (MOs) modulate multiple physiological processes, including gut health and pathogen defense. However, most research has focused on cows, and comparatively little is known about the structural diversity of MOs in the milk of other farm animals, which are commonly used for milk production, such as goats and sheep. For this reason, we used LC–MS to characterize the MOs in West African dwarf goat and East Friesian sheep milk. The analyses revealed the presence of numerous bioactive MOs. In total, 40 different MO structures were detected. Interestingly, all sheep MOs were also identified in goat milk. As especially sialylated MOs have profound functions on supporting gut health and pathogen defense in the offspring of mammals, we also quantitated the overall concentration of the sialic acids *N*-acetylneuraminic acid (Neu5Ac) and *N*-glycolylneuraminic acid (Neu5Gc), as well as the ratio of sialic acids attached to MOs, using an HPLC approach. While Neu5Gc is the dominant sialic acid in the MOs of East Friesian sheep, with Neu5Ac present only in very low concentrations, in the West African dwarf goat milk the sialic acids where more equally distributed. The obtained results are important not only for the optimization of livestock breeding, but also for humans. In this regard, Neu5Gc is particularly noteworthy, as it is supposed to induce proinflammatory effects in humans.

## Graphical abstract

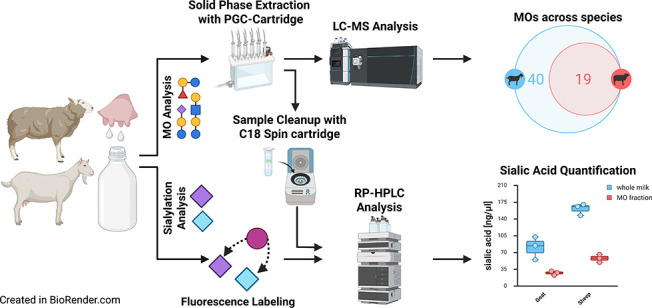

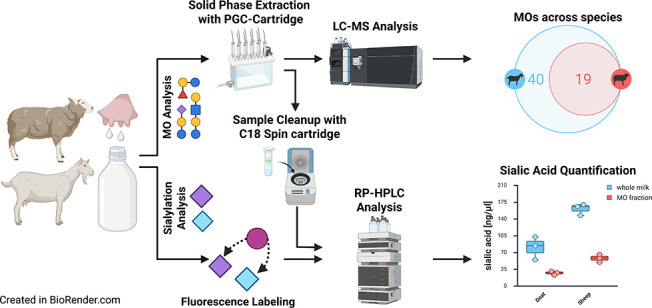

## 1 Introduction

Milk oligosaccharides (MOs) are among the most important milk components, modulating a variety of physiological processes in the offspring. For example, MOs play an important role in immune system maturation and modulate immune cell activity.^1,2^ Furthermore, MOs promote a healthy gut microbiome, since they are substrate for many beneficial microbes.^3–5^ In addition to their positive physiological effects, MOs can inhibit pathogen invasion due to their structural similarities with glycan motifs of the glycocalyx on respiratory and digestive epithelial cells, which are frequently used as docking sites by pathogens.^1^ MOs can block the binding domain of pathogen receptors, thereby preventing attachment to epithelial cells. This mechanism has been observed with several different pathogens.^1,6–10^ By inhibiting pathogens from attaching, MOs can help to prevent or counteract infection, reducing the need for antibiotics and other therapeutics.^1,7,11,12^

Various monosaccharides are used for the biosynthesis of MOs, including glucose (Glc), galactose (Gal), *N*-acetylglucosamine (GlcNAc), fucose (Fuc), and the sialic acids *N*-glycolylneuraminic acid (Neu5Gc) and *N*-acetylneuraminic acid (Neu5Ac).^11^ The majority of mammals use exclusively lactose as an initial core structure, a disaccharide of a Glc and a Gal residue.^13,14^ However, in the milk of several mammals, such as cows and water buffaloes, also a lactosamine core consisting of GlcNAc and Gal was described.^15–19^ Remarkably, the enzymatic machinery of mammary epithelial cells is able to synthesize numerous linear and branched carbohydrate structures and more than 200 different MOs have been identified.^20^ It has been suggested that a high heterogeneity of MOs is an advantage, as increasing the structural diversity of MOs would enhance their capacity to block epithelial cell binding against a broader range of pathogens.^19^

While a lot of positive functions of MOs are mediated by sialylated components, it is important to keep in mind that humans belong to a group of mammals that cannot synthesize Neu5Gc. Since Neu5Gc is not an endogenous biomolecule, it induces the release of anti-Neu5Gc antibodies.^21^ This happens because, human sialyltransferases can add Neu5Gc to the nascent glycans in the Golgi apparatus, if ingested through food^22^ and the resulting Neu5Gc containing glycoconjugates are recognized by anti-Neu5Gc antibodies, which seems to trigger inflammation and even cancer.^23,24^ For this reason, it is important to investigate the sialic acid compositions in animal products used for human consumption.

However, compared to humans and dairy cows, less is known about the presence of bioactive MOs and the sialylation in milk of other farm animals that are frequently used for milk production. In dairy cows in particular, it has been shown that there can be significant differences in MO composition depending on the breed in addition to the used analytical approaches.^25–28^ Unlike dairy cows, relatively few breeds of sheep and goats have been intensively studied, even though they also play a key role in the dairy industry. For example, only one study provides detailed information about the MO composition of milk from East Friesian dairy sheep, to the best of our knowledge.^29^ However, East Friesian sheep are a frequently used dairy breed known for their high milk production. Therefore, we characterized the MO compositions in milk from East Friesian milk sheep using different analytical approaches and also quantitated the general sialic acid concentration in the milk as well as the specifically MO-bound sialic acids. In parallel, we investigated the MOs of West African dwarf goats, which are used for both meat and milk production. To the best of our knowledge, there is currently no information available on the composition of the MOs in this breed of goat. In addition, the data obtained was compared in detail with that from other studies describing MOs in goats and sheep.

## 2 Materials and Methods

### 2.1 Samples

Mature milk from West African dwarf goats was collected from animals bred in the Research Institute for Farm Animal Biology (FBN) in Dummerstorf, Germany. East Friesian milk (mature milk) samples were shared by the Schafsscheune Vietschow (Vietschow, Germany). For each species, 3 animals were analyzed individually. One limitation of the study is that the age, day of lactation, and number of lactations of the animals studied are unknown, which could potentially influence the composition of the milk in terms of MOs. This must be taken into account when interpreting the data. The milk samples were frozen and stored at −20 °C.

### 2.2 Milk Oligosaccharide Extraction

In order to extract the MOs, the milk was first skimmed via double centrifugation at 13,000 rpm for 30 min at 4 °C. Thirty μL of skimmed milk were diluted by addition of 270 μL ddH_2_O and further purified with a solid phase extraction using a porous graphitized carbon (PGC) cartridge (25 mg PGC material, HyperSep Hypercarb SPE Cartridges, ThermoFisher Scientific) as described in detail in Isernhagen et al. according to the protocol of Blank et al.^15,30^

### 2.3 LC–MS­(/MS) Analysis of Extracted Milk Oligosaccharides

The PGC-extracted MOs were resolved in 80% ACN. A ThermoFisher Vanquish UHPLC equipped with an AccuCore-150-Amide-HILIC column was used for the separation of the MOs, as previously described.^15^ The detection occurred using mass spectrometry (MS) with the positive ionization mode being used for the neutral MOs and the negative ionization mode being used for the sialylated MOs by a Q Exactive plus Orbitrap MS (ThermoFisher, mass accuracy <3 ppm root-mean-square) directly coupled to the UHPLC system. Sialyllactose standards, 3́-sialyllactose (3́-SL) and 6́-sialyllactose (6́-SL) (Cayman Chemical Company, Ann Arbor, Michigan), were used to test the LC–MS settings for each run. According to existing literature, we collected *m*/*z* ratios of 131 known MOs to analyze the corresponding extracted ion chromatograms (EIC) in the FreeStyle Software (Thermo Fisher Scientific). In addition to the MS, we examined the MS^2^ spectra to verify and annotate the fragments according to the fragmentation results of the GlycoWorkbench 2 software.^31^ The MOs for the spectrum annotation were visualized using BioRender.com. The number of individuals, in which the specific isomer was identified, is also given in Table 1.

**Table 1:** Detected MOs in the Milk of West African Dwarf Goats and East Friesian Sheep^a^-^b^ ^c^ ^d^

ID	comp	name	category	MW	goat	sheep
1a	1_1_0_0_1	3́-Neu5Gc-lactosamine (NGLN)	acidic	690.233	3/3	3/3
1b	1_1_0_0_1	6́-Neu5Gc-lactosamine (NGLN)	acidic	690.233	3/3	3/3
2a	1_1_0_1_0	3́-Sialyllactosamine (SLN)	acidic	674.238	3/3	0/3
2b	1_1_0_1_0	6́-Sialyllactosamine (SLN)	acidic	674.238	2/3	0/3
3a	2_0_0_0_1	3́-Neu5Gc-lactose (NGL)	acidic	649.207	3/3	3/3
3b	2_0_0_0_1	6́-Neu5Gc-lactose (NGL)	acidic	649.207	3/3	3/3
4L	2_0_0_0_2	Di-Neu5Gc-lactose (DNGL) lactonized	acidic	938.287	3/3	3/3
4	2_0_0_0_2	Di-Neu5Gc-lactose (DNGL)	acidic	956.297	3/3	3/3
5a	2_0_0_1_0	3́-Sialyllactose (SL)	acidic	633.212	3/3	3/3
5b	2_0_0_1_0	6́-Sialyllactose (SL)	acidic	633.212	3/3	3/3
6L	2_0_0_1_1	Disialyllactose (DSL) heterogeneous lactonized	acidic	922.292	3/3	0/3
6	2_0_0_1_1	Disialyllactose (DSL) heterogeneous	acidic	940.302	3/3	0/3
7	2_0_0_2_0	Disialyllactose (DSL)	acidic	924.307	3/3	0/3
7L	2_0_0_2_0	Disialyllactose (DSL) lactonized	acidic	906.297	3/3	0/3
8	2_0_1_0_0	Fucosyllactose (FL)	fucosylated	488.174	3/3	3/3
9a	2_1_0_0_0		neutral	545.196	3/3	0/3
9b	2_1_0_0_0		neutral	545.196	3/3	0/3
9c	2_1_0_0_0		neutral	545.196	3/3	0/3
10a	2_1_0_1_0		acidic	836.291	3/3	0/3
10b	2_1_0_1_0		acidic	836.291	3/3	0/3
10c	2_1_0_1_0		acidic	836.291	3/3	0/3
11	2_1_1_0_0		fucosylated	691.254	2/3	1/3
12a	3_0_0_0_0	Galactosyllactose (GL)	neutral	504.169	3/3	0/3
12b	3_0_0_0_0	Galactosyllactose (GL)	neutral	504.169	3/3	3/3
13a	3_0_0_0_1	Neu5Gc-galactosyllactose (NG-GL)	acidic	811.259	3/3	3/3
13b	3_0_0_0_1	Neu5Gc-galactosyllactose (NG-GL)	acidic	811.259	3/3	3/3
14	3_0_0_0_2	Neu5Gc-Disialyl-triose	acidic	1118.350	3/3	2/3
15a	3_0_0_1_0	Sialyl-galactosyllactose (S-GL)	acidic	795.265	3/3	3/3
15b	3_0_0_1_0	Sialyl-galactosyllactose (S-GL)	acidic	795.265	3/3	0/3
15c	3_0_0_1_0	Sialyl-galactosyllactose (S-GL)	acidic	795.265	3/3	0/3
16	3_0_0_1_1	Disialyl-triose heterogeneous	acidic	1102.355	3/3	0/3
17	3_0_0_2_0	Disialyl-triose	acidic	1086.360	3/3	0/3
18a	3_1_0_0_0	Lacto-N-(neo)tetraose (LN(n)T)	neutral	707.248	1/3	3/3
18b	3_1_0_0_0	Lacto-N-(neo)tetraose (LN(n)T)	neutral	707.248	3/3	0/3
18c	3_1_0_0_0	Lacto-N-(neo)tetraose (LN(n)T)	neutral	707.248	3/3	0/3
19	3_3_0_0_0		neutral	1113.407	3/3	1/3
20	4_1_0_0_0		neutral	869.301	2/3	0/3
21	4_2_0_0_0	Lacto-N-(neo)hexaose (LN(n)H)	neutral	1072.381	3/3	3/3
22	4_2_0_0_1	Neu5Gc-Lacto-N-(neo)hexaose (NG-LN(n)H)	acidic	1379.471	3/3	2/3
23	4_2_0_1_0	Sialyl-Lacto-N-(neo)hexaose (S-LN(n)H)	acidic	1363.476	3/3	0/3

a MOs were given a specific identification code (ID) and the composition (Comp) is listed as Hex_HexNAc_Fuc_Neu5Ac_Neu5Gc.

b The ID consist of a number indicating the monosaccharide building blocks and a lowercase letter to distinguish isomers if necessary.

c An uppercase L was used to identify a lactonized form of the composition.

d The molecular weight (MW) is listed and number of the MO-positive individuals (out of 3) is given for each species.

### 2.4 Quantification of Sialic Acids

For the quantification of the oligosaccharide-bound sialic acids, the remaining lipids and proteins were removed by purifying the PGC-extracted MOs using C18 spin columns (ThermoFisher Scientific) according to the manufacturer protocol. To this end, the PGC-extracted samples were dissolved in the equilibration solution (0.5% trifluoroacetic acid (TFA) in 5% acetonitrile (ACN, Merck, Darmstadt, Germany) and loaded onto the spin column, while the flow-through was collected. To ensure complete binding of lipids and proteins, the flow-through was loaded onto the column a second time. Finally, the column was washed two times with the equilibration solution. The flow-through contained the purified MOs and was dried in a vacuum condensator and stored at −20 °C until further processing.

For the sialic acid quantification of Neu5Ac and Neu5Gc in the MO fraction and in whole milk, a 1,2-diamino-4,5-methylenedioxybenzene (DMB)-based method was performed.^32^ Subsequently, samples were hydrolyzed and derivatized with DMB.^33^ Hydrolysis was performed in 0.2 N TFA for 4 h at 80 °C. The fluorescence labeling occurred in 80 μL DMB reaction buffer (48.8 μg DMB in 9 mM sodium hydrosulfite, 0.5 M β-mercaptoethanol and 20 mM TFA) at 55 °C for 2 h and was stopped with 20 μL 0.2 N sodium hydroxide. The fluorescently labeled sialic acids were separated using a Shimadzu HPLC system (Shimadzu, Duisburg. Germany) with a C18 column (Superspher 100 RP-18 decapped LiChroCART 250-2, Sigma-Aldrich). The detection was performed with an excitation wavelength of 372 nm and an emission wavelength of 456 nm. A calibration line was conducted using commercially available standards of Neu5Ac (Carbosynth Limited, Compton, UK) and Neu5Gc (Sigma-Aldrich, Taufkirchen, Germany) (Figure S1). We used ketodeoxynonulosonic acid (KDN; Sigma-Aldrich) as an internal standard.

### 2.5 Statistical Analysis

For the statistical analysis and data visualization the “Graphs” function from BioRender.com was used, which utilizes different R packages. In this study, all the results were analyzed with Bonferroni’s multiple comparison test, and the significance levels are indicated as follows: *: *p* < 0.05, **: *p* < 0.01, ***: *p* < 0.001, ****: *p* < 0.0001. For the literature comparison of the MOs, an upset-plot was generated using the upsetR-package in R.

## 3 Results and Discussion

### 3.1 MO Compositions in Goat and Sheep Milk

For the identification of MO compositions in milk from West African dwarf goats and East Friesian sheep we used an UHPLC system equipped with an HILIC column coupled to a tandem MS system. For an optimal ionization of neutral and fucosylated MOs, the positive ionization mode was used where we detected the [M + H]^+^ and [M + Na]^+^ adducts. For sialylated MOs the ionization process shows better results in the negative ionization mode and the deprotonated ions [M-H]^−^ and [M-2H]^2–^ were analyzed. For verification and to possibly get additional information, such as the glycosidic linkages, we performed MS/MS analysis as exemplary shown for 2 neutral and 5 sialylated MOs in Figure 1.

**Figure 1:**
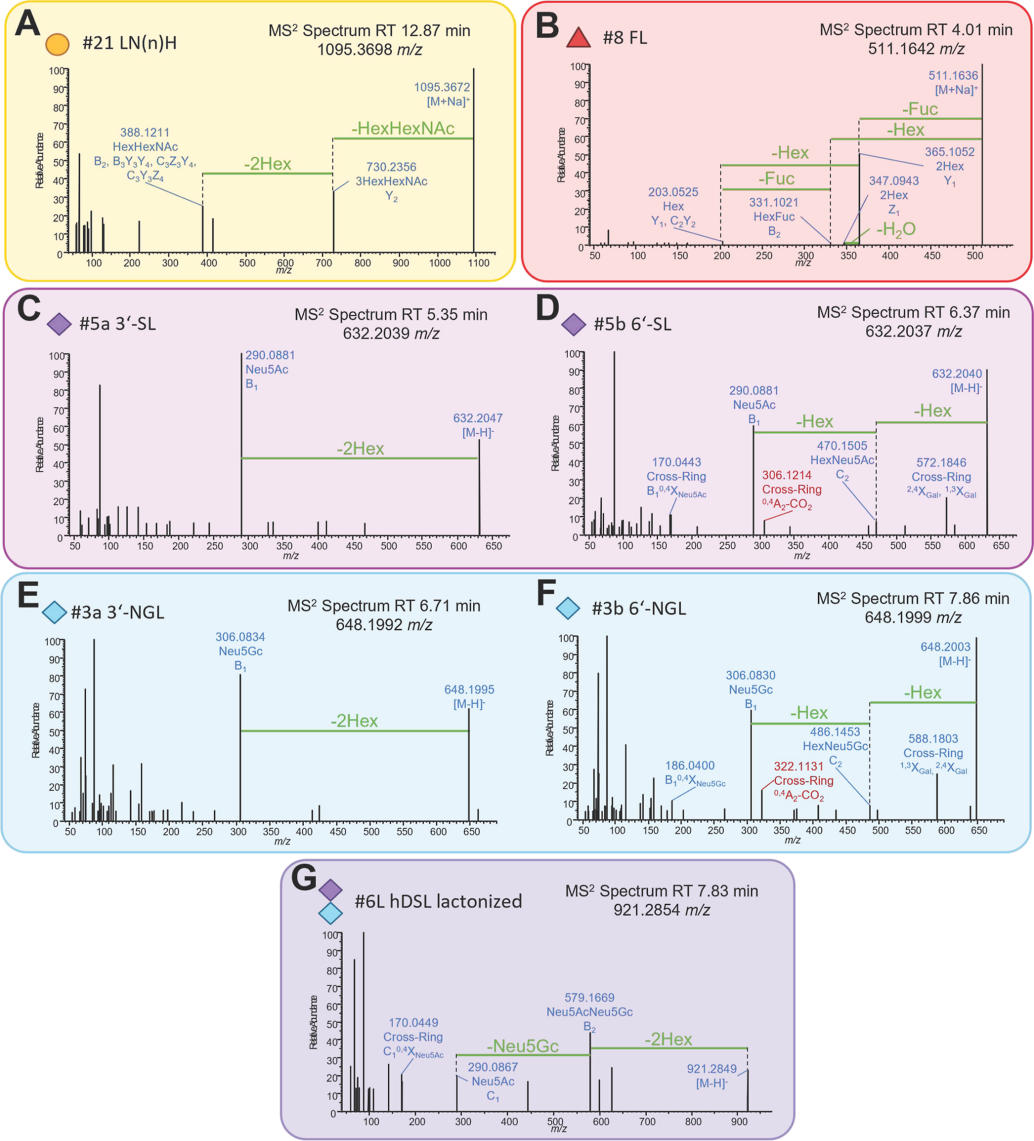
MS/MS spectra for (**A**) lacto-*N*-(neo)­hexaose (LN­(n)­H; #21); (**B**) fucosyllactose (FL, #8); (**C**) 3́-sialyllactose (3́-SL; #5a); (**D**) 6́-sialyllactose (6́-SL; #5b); (**E**) 3́-Neu5Gc-lactose (3́-NGL; #3a); (**F**) 6́-Neu5Gc-lactose (6́-NGL; #3b) and (**G**) heterogeneous DSL (hDSL; #6L) in its lactonized form to show the Neu5Ac-Neu5Gc-fragment. MS/MS spectra of all MOs are shown in the Figure S2. The different MO categories were visualized with a symbol in the upper left corner and with a transparent background color: yellow + circle: neutral nonfucosylated; red + triangle: neutral fucosylated; purple diamond and pastel violet background: Neu5Ac-sialylated; light blue diamond and pastel blue background: Neu5Gc-sialylated; purple and blue diamonds and pastel purple background: heterogeneous sialylation with Neu5Ac and Neu5Gc. Created in BioRender. Isernhagen, L. (2026) https://BioRender.com/py0kauv.

A total of 40 MOs were identified in West African dwarf goat milk and 19 of these MOs were also detected in the East Friesian sheep samples (Figure 2 and Table 1). Three of the MOs in goats and four of the MOs in sheep were not found in the milk of all animals (Table 1). When comparing the different MOs across the analyzed species, clear differences emerge in the ratio of MO categories: neutral nonfucosylated, neutral fucosylated, Neu5Ac-sialylated, Neu5Gc-sialylated, and Neu5Ac-Neu5Gc-sialylated (Figure 2C). While 11 of 40 (28%) of the identified West African dwarf goat MOs were neutral and nonfucosylated, 4 out of 19 (21%) East Friesian sheep MOs were neutral and nonfucosylated, namely an isomer of galactosyllactose (GL; 3_0_0_0_0; Hex_HexNAc_Fuc_Neu5Ac_Neu5Gc; #12b), one isomer of lacto-N-tetraose (LNT; 3_1_0_0_0; #18a), the composition 3_3_0_0_0 (#19) and lacto-N-hexaose (LNH; 4_2_0_0_0; #21). Additional neutral, nonfucosylated MOs were identified in West African dwarf goat milk samples. These included further isomers of the aforementioned sheep MOs, such as GL and LNT. Furthermore, we identified 3 isomers of the composition 2_1_0_0_0 (#9a-c) and the composition 4_1_0_0_0 (#20). Looking at the MOs LNT and LNH, a clear distinction between the different isomers was not possible during this study due to the lack of linkage-specific fragments. Therefore, both isomers could also be present in the lacto-N-neo-tetraose/-hexaose linkage (LNnT/LNnH).

**Figure 2:**
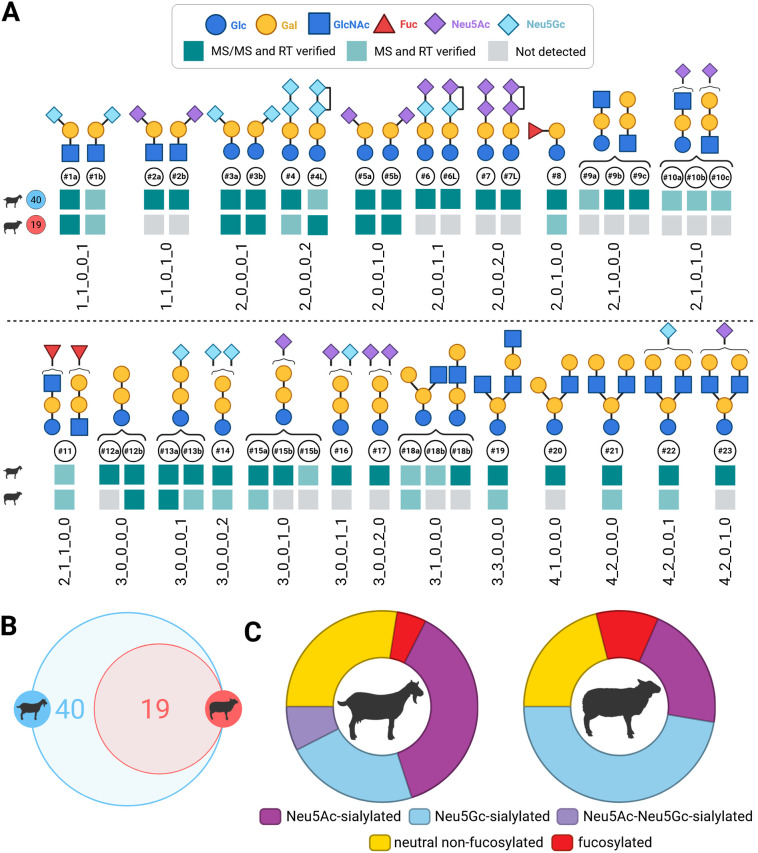
Milk oligosaccharide distribution across both species. (**A**) The detected MO compositions were visualized and the type of detection was indicated by the color of the corresponding square. For some MOs, the specific linkages and order of monosaccharides were identified through the fragmentation pattern. For others, the proposed structures were derived from different literature sources.^39,40^ (**B**) Venn-Diagramm (**C**) Donut Charts showing the number of observed MOs in each category in relation to each other for each species. Created in BioRender. Isernhagen, L. (https://BioRender.com/ecb5qx2).

In addition, we identified only 2 fucosylated neutral compositions, fucosyllactose (FL; 2_0_1_0_0; #8) and the composition 2_1_1_0_0 (#11), which were present in both analyzed species (Figure 2A). Therefore, fucosylation appears to play a minor role in the milk of these farm animals. This has also been demonstrated in bovine and buffalo milk,^15,19,34^ which differs greatly from human breast milk. The majority of human MOs are fucosylated.^35^

The most abundant MO category in the milk of West African dwarf goats and East Friesian sheep was the sialylated group. Examining the type of sialylation reveals further differences in this group of MOs. The majority of the detected MOs in East Friesian sheep milk were sialylated with Neu5Gc (9 out of 13 sialylated MOs). This is supported by other studies showing that most ovine MOs are sialylated with Neu5Gc.^17,29,34,36,37^ While all the Neu5Gc-monosialylated MOs detected in this study were present in both species, the ratio of sialylated MOs in the milk of West African dwarf goats was more evenly distributed. This milk contained 15 Neu5Ac- and 9 Neu5Gc-monosialylated structures, as well as three heterogeneous disialylated structures. This finding is consistent with the results of a 2021 study investigating the distribution of various categories among Dutch Saanen (Melkgeit) goats.^38^ The heterogeneous double sialylation with both, Neu5Ac and Neu5Gc, was only present in West African dwarf goat milk, where heterogeneous disialyllactose (hDSL; 2_0_0_1_1; #6) and its lactonized form (#6L), as well as heterogeneous disialyl-triose (3_0_0_1_1; #16) were detected. This was not occurring in the analyzed samples of East Friesian sheep. Interestingly, disialyllactose (DSL, 2_0_0_2_0, #7), which is one of the most abundant MOs in Holstein-Friesian cows and water buffaloes,^15,19^ was not detected in the East Friesian sheep milk.

While goats and sheep MOs share the same neutral backbone, the sialic acid used to terminate their compositions often differs. The sialylated MOs detected across both species in this study ranged from 3 monosaccharides, for example 3́-Neu5Gc-lactosamine (NGLN; 1_1_0_0_1; #1a), to 7 building blocks with the compositions 4_2_0_0_1 (#22) and 4_2_0_1_0 (#23). To distinguish between different sialic acid linkages, we used commercially available 3́- and 6́-sialyllactose (3́- and 6́-SL; 2_0_0_1_0; #5a and #5b). In addition, the linkage was verified by a specific MS^2^ fragment, which can only occur in the 6́-sialic acid linkage as shown in Figure 1D,F.^41^ This enabled a retention time assumption for other sialylated MOs with 3 building blocks like NGLN (1_1_0_0_1; #1a and #1b), sialyllactosamine (SLN; 1_1_0_1_0; #2a and #2b) and Neu5Gc-lactose (NGL; 2_0_0_0_1; #3a and #3b). A determination of other sialic acid linkages for MOs with more than 3 building blocks was not possible, since the increasing size of MOs comes along with an increase of further factors, which influence the retention time.

In comparison to previous studies, several differences were observed in the outlined study. The first description of goat MOs was published in 1988 by Chaturvedi et al. They isolated three goat MOs, namely 2_1_0_0_0, 3_1_0_0_0, and 4_1_0_0_0 ^42^ and a few years later two isomers for the fucosylated composition 3_1_1_0_0.^43^ Another group also described FL (2_0_1_0_0) and 3 isomers for a triose composition (3_0_0_0_0).^44^ The first sialylated goat MOs were described in 1997, when Urashima et al. detected 6́-SLN (1_1_0_1_0), 6́-NGL (2_0_0_0_1) and 3́- and 6́-SL (2_0_0_0_1).^45^ In the same year, Viverge et al. also detected 3́- and 6́-Sialyl-galactosyllactose (S-GL; 3_0_0_0_1).^46^ In 2006, Martinez-Ferez newly described 12 additional MOs, including disialyllactose (DSL; 2_0_0_0_2) and hDSL (2_0_0_1_1) as well as other disialylated and neutral compositions, but detected no fucosylated MOs in caprine milk.^47^ All of these MOs were also detected a few years later by Meyrand et al., while also detecting 11 new MOs including five fucosylated MOs in addition to FL (2_0_1_0_0).^48^ A total of 142 MOs were described in 20 publications about goat milk. Excluding publications that described fewer than 20 MOs,^38,42–46,49–53^ nine publications remain for a general overview of the MO spectrum of goat milk. These publications identified 141 different goat MOs.^16,29,34,36,47,48,54–56^ Remarkably, only 54 goat MOs were described in more than two references. During our analysis, we verified 40 MOs in goat milk, 4 of which were previously described by only 1 study and 33 of the MOs described by more than 2 publications. We were able to verify the presence of all 6 MOs described by all of the 9 publications and the vast majority of the MOs described in at least 5 studies. The 40 MOs detected in West African dwarf goats include 3 lactonized isomers of disialylated MOs.

One possible explanation for the discrepancies between studies analyzing goat’s milk is that some publications investigated colostrum, whereas in other studies, including our study, mature milk was analyzed (Figure 3 and Table S1). Since it was previously described that most MOs decrease or even disappear during lactation,^55^ this may explain why some of the less frequently observed structures were not detected. Furthermore, the breed, the age and number of lactations of the animals studied, in addition to the analytical strategy could be a contributing factor to the observed differences.

**Figure 3:**
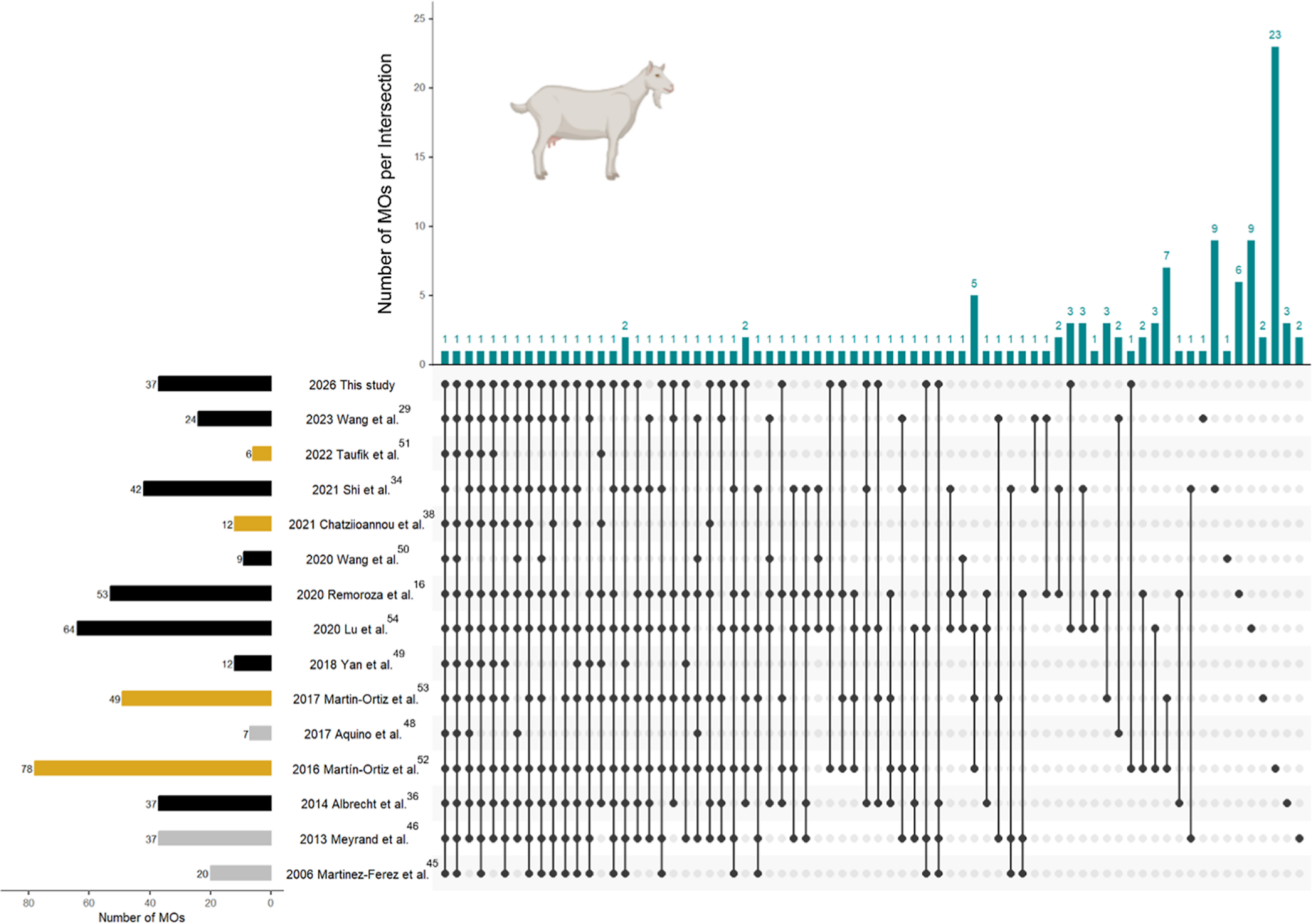
Literature comparison of MOs in goat milk. The upset-plot was generated using the upsetR-package in R. In the bottom-left part the number of MOs per publication is visualized. The color of the bar indicates the use of mature milk (black), colostrum (golden) or an unknown milk type (gray). The first line represents the results of this study. In the lower right part, the studies with common MOs are indicated with the number of MOs found in that specific combination of references visualized as a column graph in the upper part of the plot. The lower left part indicates the number of MOs found in each study. A literature comparison is not possible to a sufficient extent for the lactonization, as few, if any, publications search for the lactonized *m*/*z* ratio. Therefore, lactonized MOs were excluded. For better readability, this figure only includes references with at least 5 MOs detected. The extended version can be found in the Figure S3. Modified in BioRender. Isernhagen, L. (2026) https://BioRender.com/7zsgacp.

The same applies to sheep milk. The first study of sheep MOs was performed in 1989, where the 3 neutral MOs 3́- and 6́-GL (3_0_0_0_0) and a newly discovered galactosyllactose with α-glycosidic configuration (Galα-3Galβ1-4Glc) were found.^57^ In a later study, the five sialylated ovine MOs 3́-SL (2_0_0_1_0), 6́-NGL (2_0_0_0_1) and three forms of 3́-NGL (2_0_0_0_1) including two lactonized forms were detected.^58^ Albrecht et al. found 35 ovine MOs in colostrum.^36^ They described three fucosylated structures, namely 3-fucosyllactosamine (3-FLN; 1_1_1_0_0) and 2́- and 3-FL (2_0_1_0_0). Another study found 32 MOs in mature sheep milk with 5 fucosylated structures, including the newly proposed fucosylated compositions 2_0_1_1_0, 2_0_1_0_1, 2_0_2_0_0 and 2_2_1_0_0. The authors described higher fucosylation, as well as double fucosylation. Two of the fucosylated MOs were also sialylated.^34^ One of the most recent studies on sheep MOs, from 2023, detected only FL as a fucosylated structure.^37^ Another study, however, described both FL and difucosyllactose.^29^ We were also able to identify FL (#8) but none of the other fucosylated MOs described by Albrecht et al. and Shi et al. In contrast, we detected a fucosylated sheep MO with the composition 2_1_1_0_0 (#11).

Looking at the number of MOs detected in the 8 publications about sheep MOs, it becomes evident that 3 have focused their analysis on specific MOs.^51,57,58^ To the best of our knowledge, only 5 publications so far have described the MO distribution in sheep milk in detail detecting in sum 51 sheep MOs.^17,29,34,36,37^ Comparing those studies, the heterogeneous description of the MO distribution in sheep becomes evident (Figure 4). Only 9 of 51 were found in all five studies, two in four, seven in three, 13 in two, and the remaining 20 were found in only one study. While we did not identify 35 of those MOs (including 11 isomers of identified MOs from this study), the majority were only described in less than 3 of the 5 studies. In total we were able to support the presence of 16 of those 51 MOs in our analysis. Of the ovine MOs detected in all five references, the majority were also found in this study. However, hDSL (2_0_0_1_1), DSL (2_0_0_2_0) and 2_1_0_0_0 were only found in goat samples, not sheep milk. In addition, we detected three newly proposed sheep MOs in our samples. These include an additional isomer for Neu5Gc-GL (#13), where we detected 2 distinct retention times, as well as the lactonized form of Di-Neu5Gc-lactose (DNGL; 2_0_0_0_2; #4L). In addition, we detected a further fucosylated MO in this study (2_1_1_0_0; #11). In sum, we detected 19 MOs in sheep milk with a high proportion of Neu5Gc-containing MOs. Although the overall proportion of the various MO categories aligns with our findings in this study, we could only validate 16 out of 51 described MOs. As mentioned above, the discrepancies between our findings and those reported in the literature could be due to differences in analytical methods and/or the breeds as well as the type of milk, as all these factors can influence the obtained results.^49,56^ While Albrecht et al. and Wang et al. specifically stated the breed and that the samples were colostrum,^29,36^ others only stated the research institute or farm where the sheep milk samples were obtained, rather than the breed or the specific sampling time point (Table S1). This makes it difficult to compare results, which highlights the importance of providing sample information.

**Figure 4:**
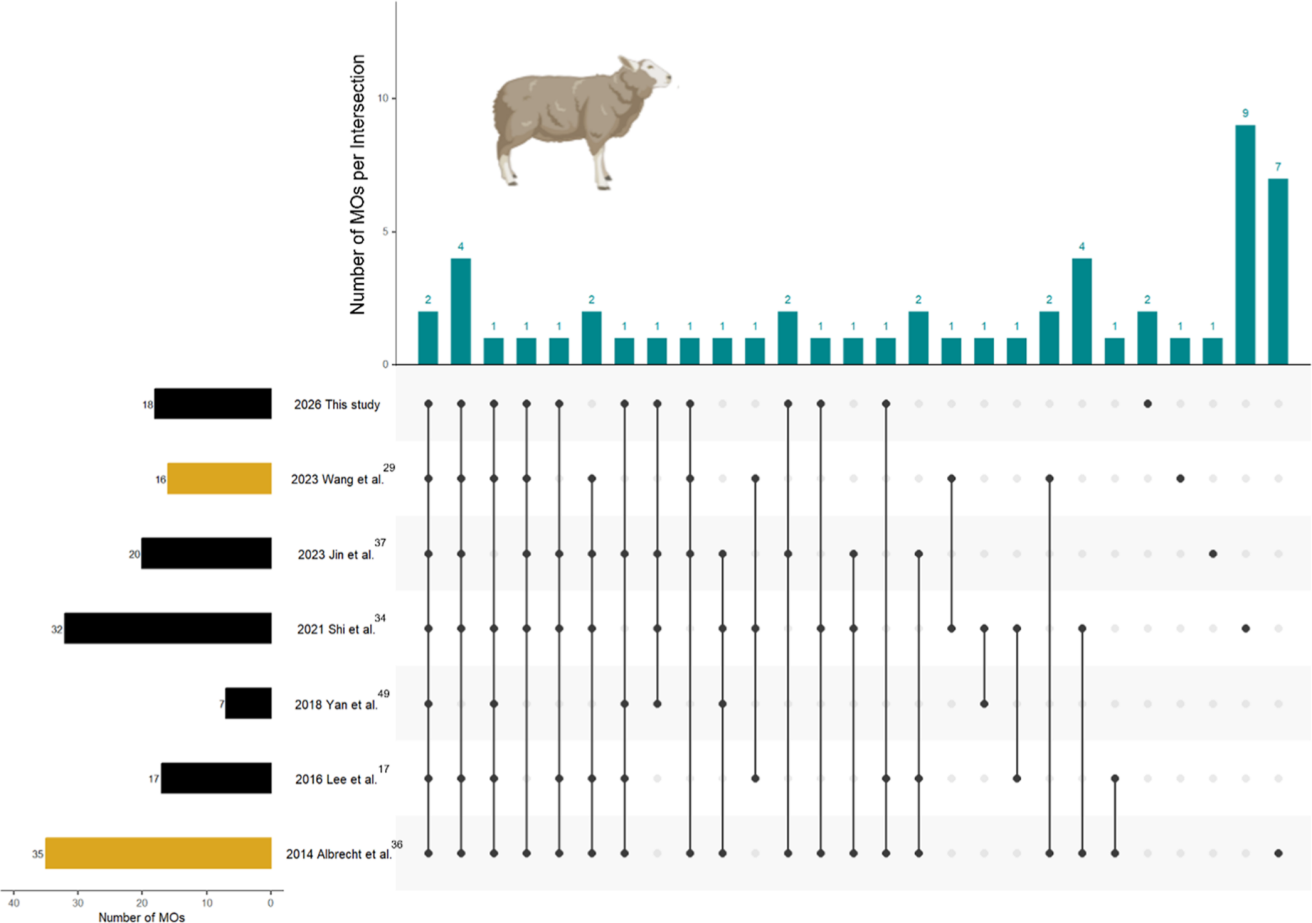
Literature comparison of MOs in sheep milk. The upset-plot was generated using the upsetR-package in R. In the bottom-left part the number of MOs per publication is visualized. The color of the bar indicates the use of mature milk (black) or colostrum (golden). The first line represents the results of this study. In the lower right part, the studies with common MOs are indicated with the number of MOs found in that specific combination of references visualized as a column graph in the upper part of the plot. The lower left part indicates the number of MOs found in each study. A literature comparison is not possible to a sufficient extent for the lactonization, as few, if any, publications search for the lactonized *m*/*z* ratio. Therefore, lactonized MOs were excluded. For better readability, this figure only includes references with at least 5 MOs detected. The extended version can be found in Figure S3. Modified in BioRender. Isernhagen, L. (2026) https://BioRender.com/ylp5kso.

### 3.2 Sialic Acid Quantitation

Because of the proinflammatory capacity of Neu5Gc in humans and the high number of Neu5Gc-containing MOs in goat and sheep milk in this study, we performed a detailed analysis of the sialylation status of the whole milk as well as the MO fraction. To quantify Neu5Gc and Neu5Ac, sialic acids were released under acidic conditions and then labeled with a fluorescent dye for analysis by HPLC (Figure 5A). The concentration of MO-bound Neu5Ac was lowest in sheep milk (2.6 ng/μL), while in goat milk 38.7 ng/μL were detected (Figure 5B). The results for Neu5Gc were reversed, with East Friesian sheep’s milk having a higher concentration (58.1 ng/μL), while West African dwarf goat’s milk had a lower concentration (27.3 ng/μL). Thus, over 90% of the MO fraction in sheep was responsible for Neu5Gc, whereas the proportions of Neu5Ac and Neu5Gc in goat’s MOs were almost comparable. Comparable ratios of Neu5Gc and Neu5Ac were detected, when whole milk was analyzed (Figure 5D), which is in line with previous studies.^36,59,60^ The results are in line with the LC–MS analysis of the MOs demonstrating that small ruminants synthesize large amounts of Neu5Gc containing MOs in comparison to large ruminants.^15,19^

**Figure 5:**
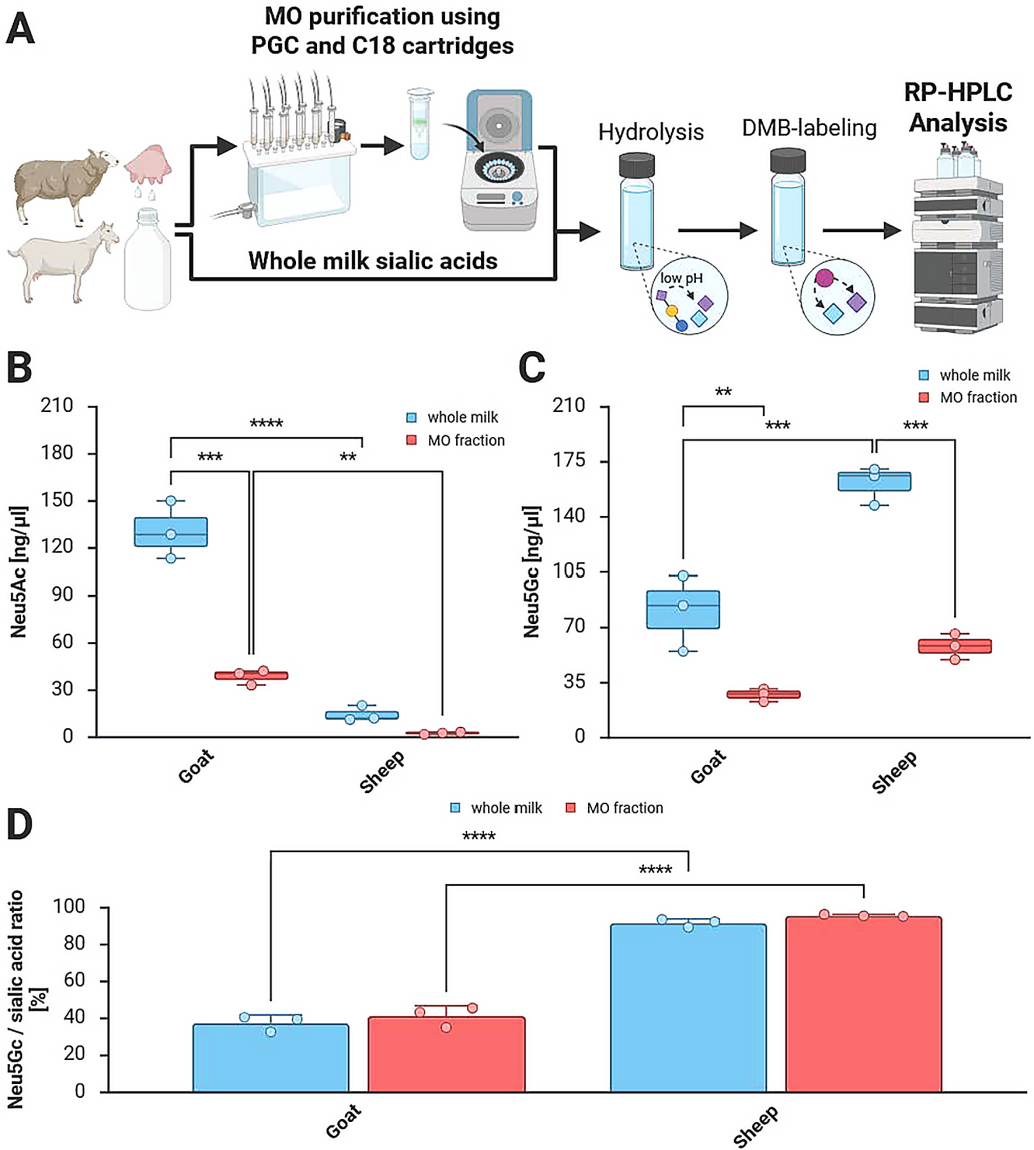
Quantification of sialic acids in West African dwarf goat and East Friesian sheep milk. (**A**) Scheme of the DMB-RP-HPLC strategy for sialic acid quantification, where the sialic acids from the whole milk as well as the PGC-enriched and C18-purified MOs were released by hydrolysis and labeled for subsequent fluorescence detection. Box and whisker plots (median; min to max) showing the (**B**) Neu5Ac and (**C**) Neu5Gc values in the analyzed milk samples for each species (*n* = 3 animals per species). (**D**) Ratio of Neu5Gc compared to the sum of Neu5Ac and Neu5Gc (sialic acids). The detailed data can be found in Table S2. Created in BioRender. Isernhagen, L. (2026) https://BioRender.com/17milrf.

## 4 Conclusion

Analyses revealed the presence of numerous bioactive MOs in West African dwarf goats and East Friesian dairy sheep. However, several differences were observed. In West African dwarf goats, comparable ratios of MO structures are elongated with Neu5Gc and Neu5Ac, whereas sialylated MOs in East Friesian sheep contain prevalently Neu5Gc. Remarkably, in large ruminants, such as buffalo and dairy cows, Neu5Gc containing MOs belong to subfractions of MOs and are only present in very small quantities. Moreover, it should be particularly noted that dominant buffalo and bovine Neu5Ac containing MOs, such as SLN or DSL,^15,19^ were detected in East Friesian sheep exclusively in the Neu5Gc containing version. As the conversion of Neu5Ac to Neu5Gc requires additional energy, the high levels of Neu5Gc found in goats and sheep suggest that Neu5Gc plays an essential role in the offspring of small ruminants. An important function of sialylated MOs is to block the glycan-binding receptors of pathogens that are required for them to bind to the glycocalyx of epithelial cells.^61^ This is possible because the structure of many MOs corresponds to glycan motifs of the glycocalyx that pathogens use as docking sites. In this context, it could be that pathogens that infect these animal species primarily use Neu5Gc-containing glycans of the glycocalyx as binding sites. Therefore, it would be advantageous for a higher proportion of MOs to contain Neu5Gc. Another possibility is that Neu5Gc is important for the microbiome in the offspring of small ruminants, since they are important substrates for beneficial microbes. However, neither possibility is yet known, so further research in this area is needed to test the hypothesis. In terms of healthy human nutrition, it is important to note that a high proportion of Neu5Gc in food can trigger inflammatory responses. This is because dietary Neu5Gc from goat’s or sheep’s milk is incorporated into the glycocalyx of endothelial cells and other cell types, which are then targeted by anti-Neu5Gc antibodies. This pathological mechanism is discussed to trigger inflammation and cancer. For this reason, some scientific studies recommend reducing the consumption of foods containing Neu5Gc.^62,63^

## Supplementary Material











## Notes

Figure S1: Additional Information on the method section of the sialic acid quantification (PDF)
Figure S2: MS spectra of MOs found in West African dwarf goats and East Friesian sheep (PDF)
Figure S3: Extended version of the literature comparison visualization for goat and sheep MOs (PDF)
Table S1: Literature comparison for goat and sheep MOs (PDF)
Table S2: Sialic acid quantification (PDF)

## Abbreviations

ACN: acetonitrile
DMB: 1,2-diamino-4,5-methylenedioxybenzene
DNGL: Di-Neu5Gc-lactose
DSL: disialyllactose
EIC: extracted ion chromatogram
FL: fucosyllactose
Fuc: fucose
Gal: galactose
GL: galactosyllactose
Glc: glucose
GlcNAc: *N*-acetylglucosamine
hDSL: heterogeneous disialyllactose
HILIC: hydrophilic interaction liquid chromatography
HPLC: high performance liquid chromatography
LC: liquid chromatography
LNnH: lacto-N-neohexaose
LNH: lacto-N-hexaose
LNnT: lacto-N-neotetraose
LNT: lacto-N-tetraose
MOs: milk oligosaccharides
MS: mass spectrometry
Neu5Ac: *N*-acetylneuraminic acid
Neu5Gc: *N*-glycolylneuraminic acid
NG-GL: Neu5Gc-galactosyllactose
NGL: Neu5Gc-lactose
NGLN: Neu5GC-lactosamine
PGC: porous graphitized carbon
RP-HPLC: reverse phase high performance liquid chromatography
rpm: repetition per minute
RT: room temperature
S-GL: Sialyl-galactosyllactose
SL: Sialyllactose
SLN: Sialyllactosamine
TFA: trifluoroacetic acid
UHPLC: ultra high performance liquid chromatography
