# Supplementary material for: Analysis of the Milk Oligosaccharides Spectrum and Sialylation Status of Milk from West African Dwarf Goat and East Friesian Sheep
Source: ACS Omega. 2026 May 27;11(22):32310–20. doi: 10.1021/acsomega.5c13396 (PMC13261409; doi:10.1021/acsomega.5c13396)
Supplement: Supplementary file 1 [file ao5c13396_si_001.pdf]

Figure S1
Analysis of the Milk Oligosaccharides Spectrum and Sialylation Status of Milk from West African Dwarf Goat and East Friesian Sheep

Lisa Isernhagen[a], Christina E. Galuska[a], Andreas Hoeflich[a] and Sebastian P. Galuska[a, *]

[a]: Research Institute for Farm Animal Biology (FBN), Wilhelm-Stahl-Allee 2, 18196 Dummerstorf, Germany

[*]: Corresponding author: Sebastian P. Galuska, Galuska.sebastian@fbn-dummerstorf.de

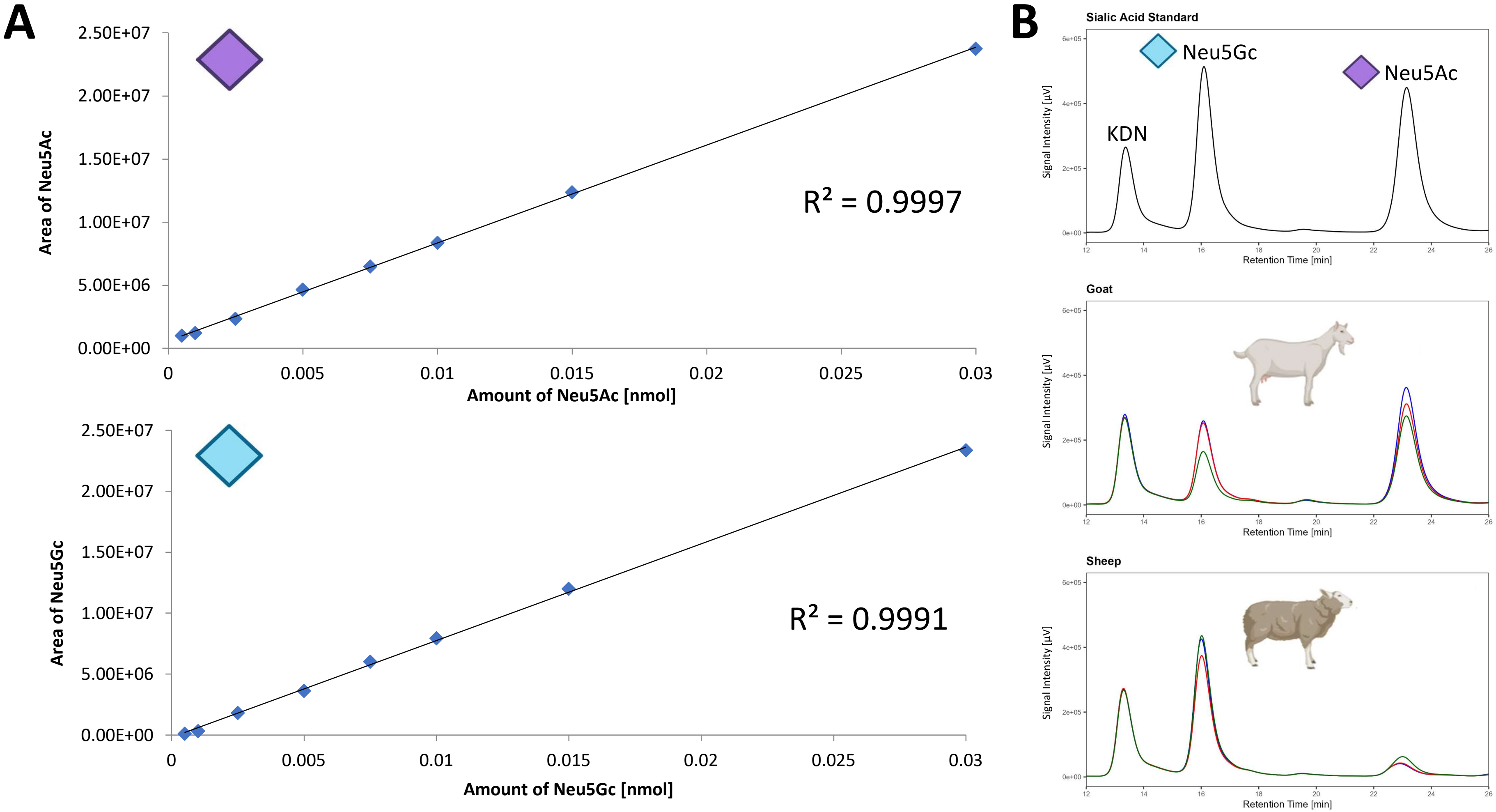

Figure S1: Additional information on the sialic acid quantitation using a DMB-based RP-HPLC protocol. **(A)** Calibration line of a commercially available Neu5Ac and Neu5Gc standard in different concentrations (0.0005 – 0.03 nmol). A linear trend line was added and the regression coefficient R^2 is given. The peak areas are extracted from the LabSolutions Software and the analysis was performed in Microsoft Excel. **(B)** An exemplary partial chromatogram of a sialic acid mixture with the same amount of Neu5Ac and Neu5Gc (0.03 nmol) in addition to the internal standard KDN (0.015 nmol) with annotated peaks. Chromatogram Overlay of the analyzed milk samples of goat and sheep with KDN being the same amount of KDN added as in the standard line in (A). The chromatograms were generated in R. The added pictures of Neu5Ac, Neu5Gc, Goat and Sheep were generated in BioRender.com.
