# Supplementary material for: Analysis of the Milk Oligosaccharides Spectrum and Sialylation Status of Milk from West African Dwarf Goat and East Friesian Sheep
Source: ACS Omega. 2026 May 27;11(22):32310–20. doi: 10.1021/acsomega.5c13396 (PMC13261409; doi:10.1021/acsomega.5c13396)

Figure S2

Analysis of the Milk Oligosaccharides Spectrum and Sialylation Status of Milk from West African Dwarf Goat and East Friesian Sheep

Lisa Isernhagen[a], Christina E. Galuska[a], Andreas Hoeflich[a] and Sebastian P. Galuska[a, *]

[a]: Research Institute for Farm Animal Biology (FBN), Wilhelm-Stahl-Allee 2, 18196 Dummerstorf, Germany

[*]: Corresponding author: Sebastian P. Galuska, Galuska.sebastian@fbn-dummerstorf.de

West African Dwarf Goat

Figure description

- MS spectra of MOs found in West African dwarf goats at the corresponding retention time extracted from the ThermoFisher Scientific Software Freestyle.
- The MO categories are indicated in the upper-left corner by a symbol obtained from BioRender.com and the fragments were computed using GlycoWorkbench 2 (Ceroni et al., 2008).
- The MO structures on page 20 and 21 were visualized with BioRender.com.
- Fragments, which were linkage or order-specific, were highlighted in red.
- yellow circle, neutral-nonfucosylated; red triangle, neutral-fucosylated; purple diamond, Neu5Ac-sialylated; light blue diamond, Neu5Gc-sialylated

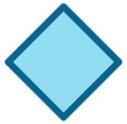

#1a 3‘-Neu5Gc-lactosamine (3‘-NGLN)

MS^2 Spectrum RT 6.12 min
689.2267 *m/z*

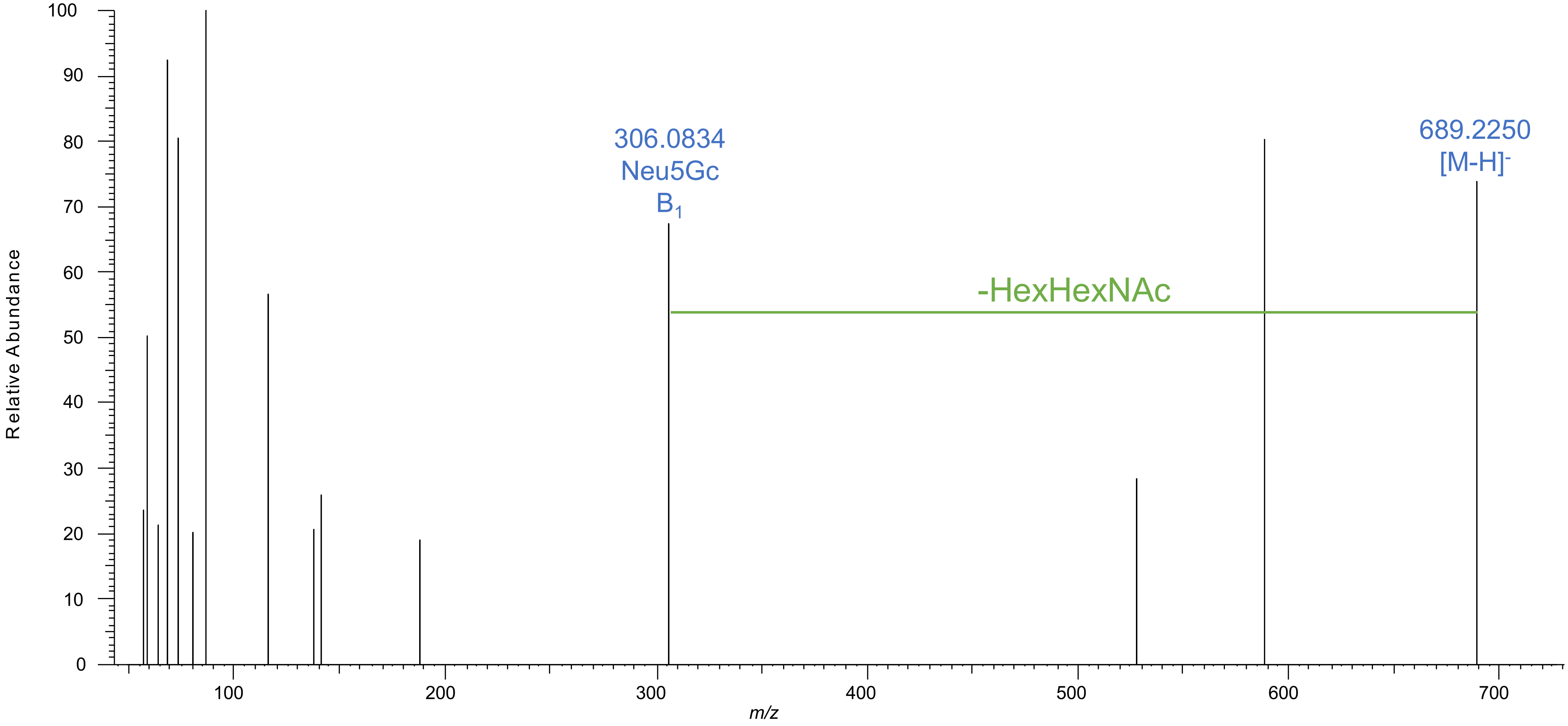

#1b 6‘-Neu5Gc-lactosamine (6‘-NGLN)

MS^2 Spectrum RT 6.53 min
No MS^2 Spectrum,
RT verified with other samples

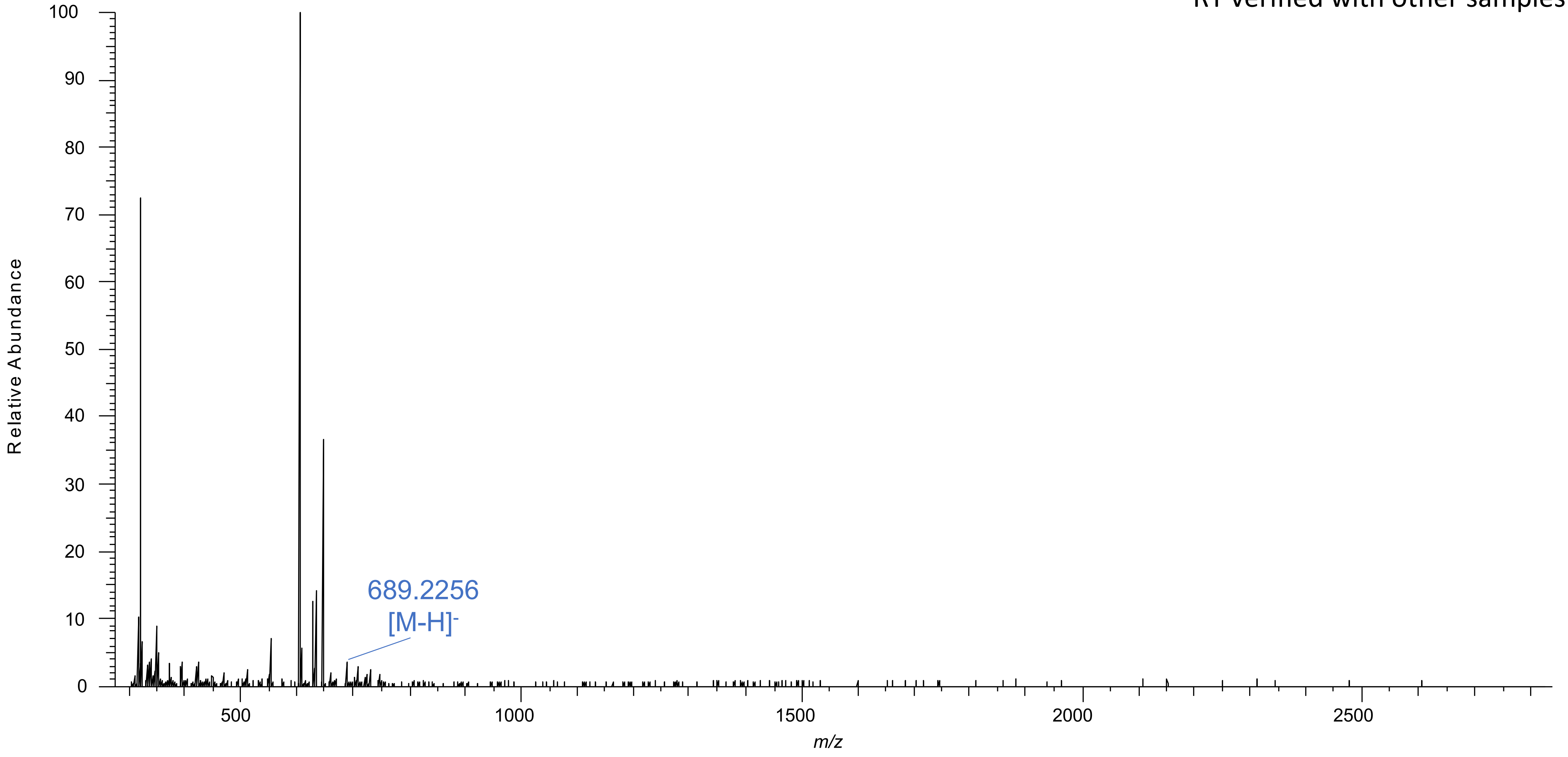

#2a 3‘-Sialyllactosamine (3‘-SLN)

MS^2 Spectrum RT 4.95 min
673.2313 *m/z*

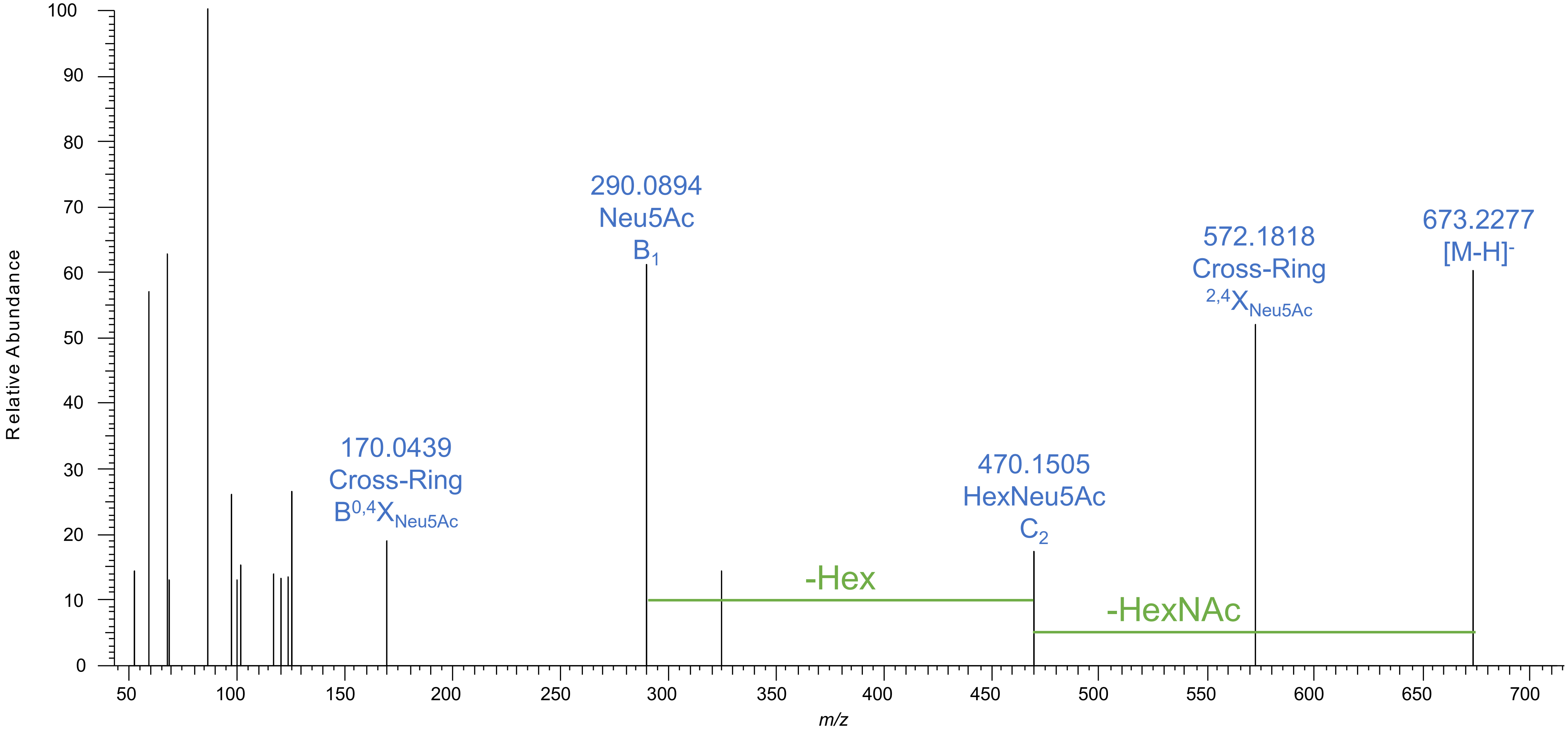

#2b 6‘-Sialyllactosamine (6‘-SLN)

MS^2 Spectrum RT 5.27 min
673.2302 *m/z*

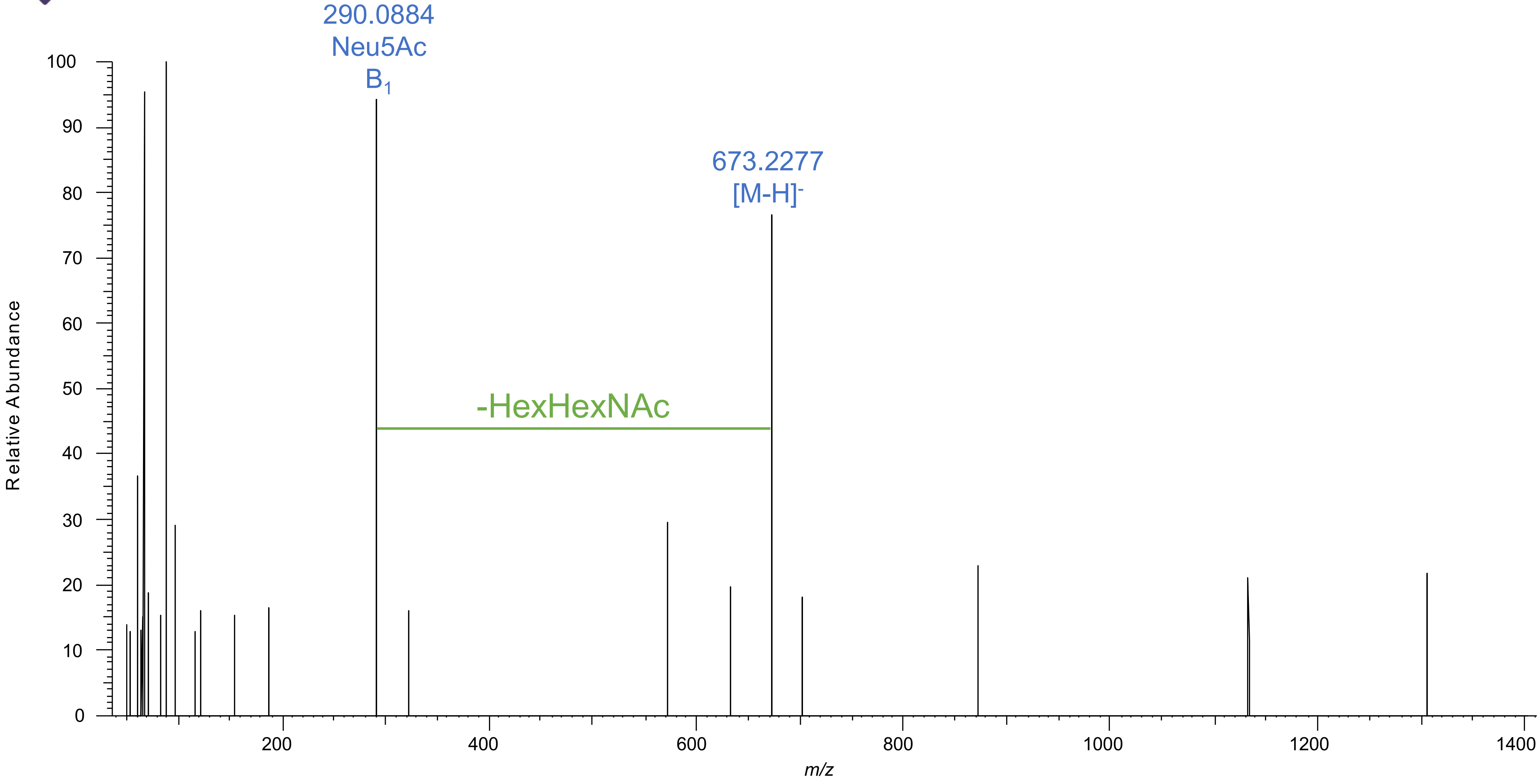

#3a 3‘-Neu5Gc-lactose (3‘-NGL)

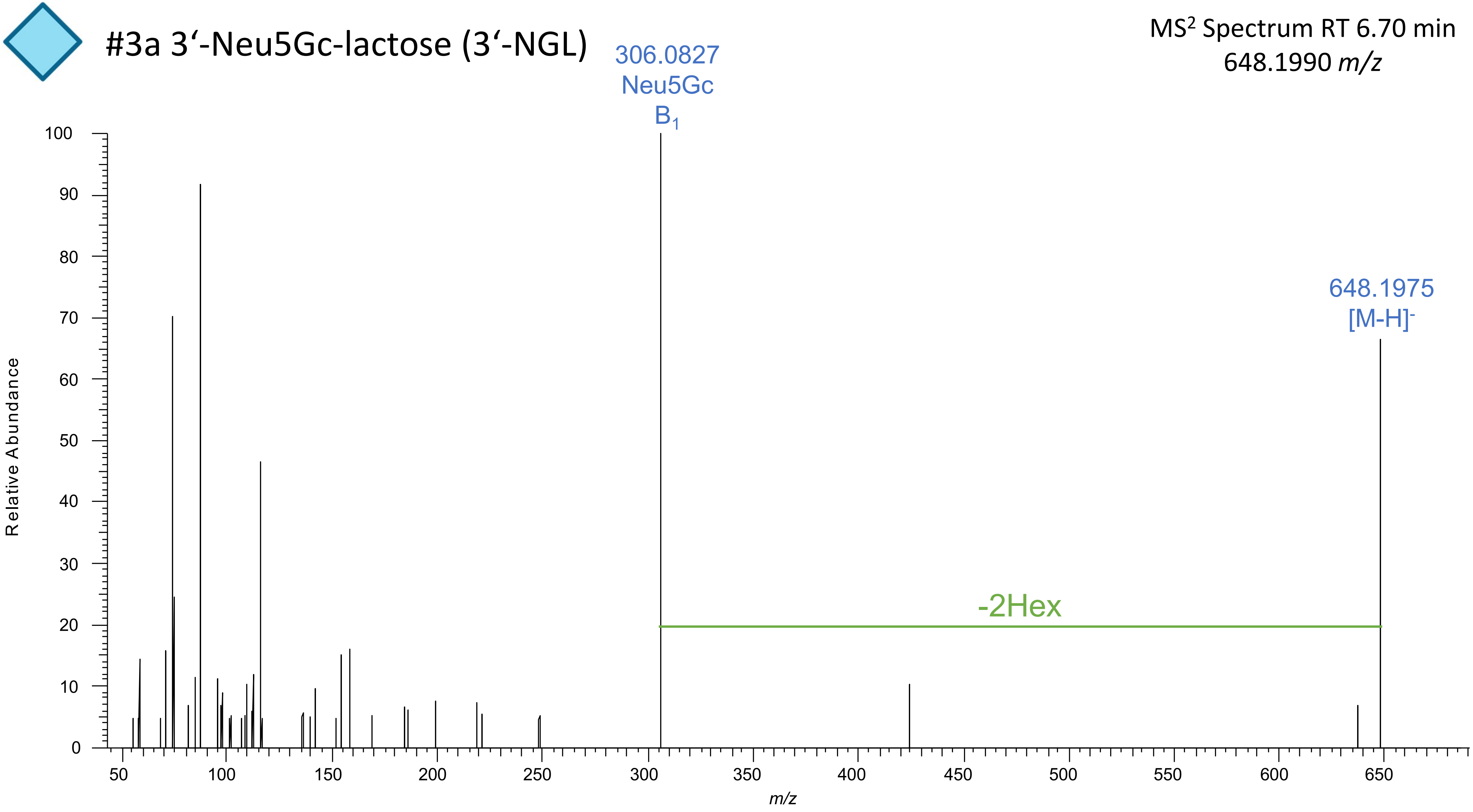

#3b 6‘-Neu5Gc-lactose (6‘-NGL)

MS^2 Spectrum RT 7.86 min
648.1997 *m/z*

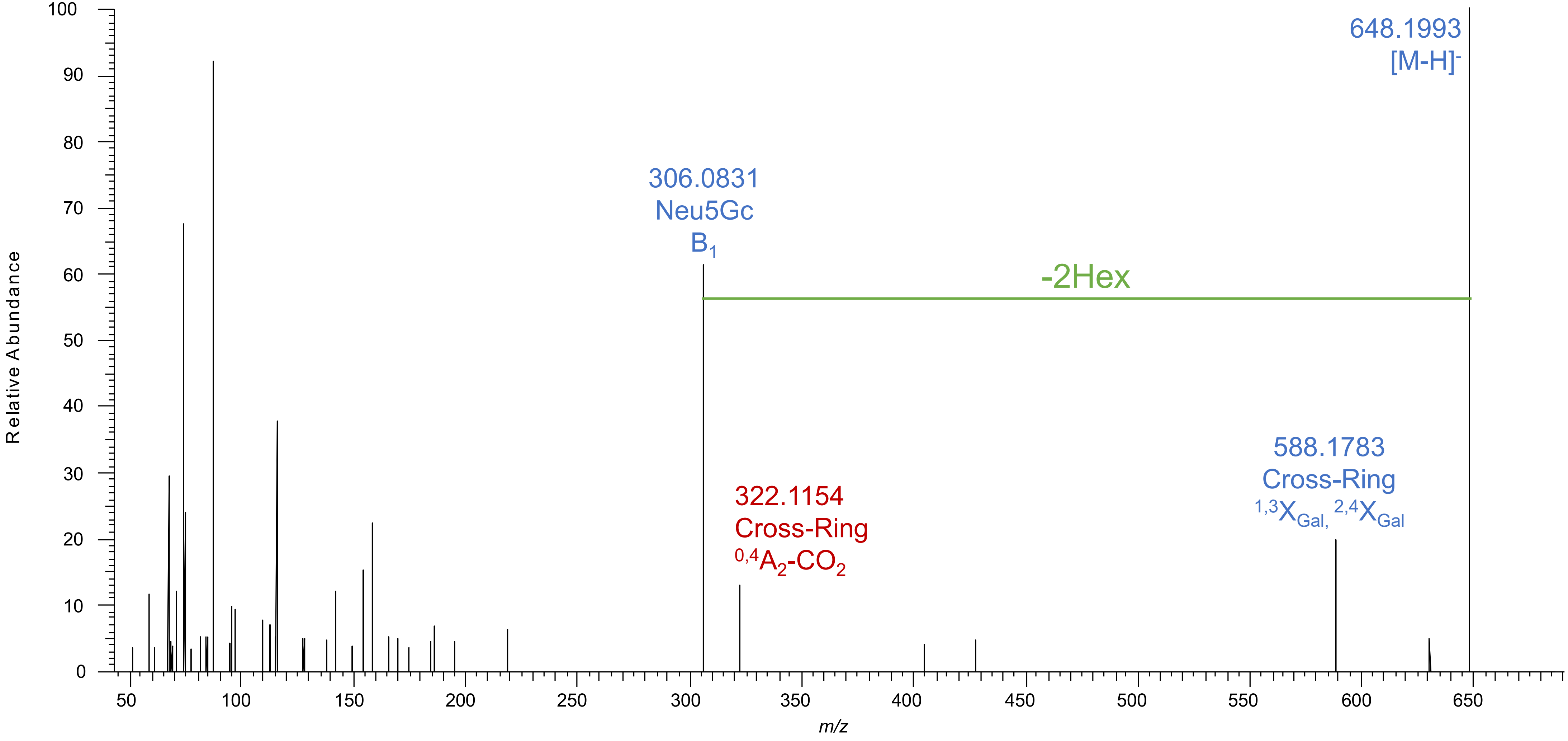

#4 Di-Neu5Gc-lactose (DNGL)

MS^2 Spectrum RT 12.77 min
955.2911 *m/z*

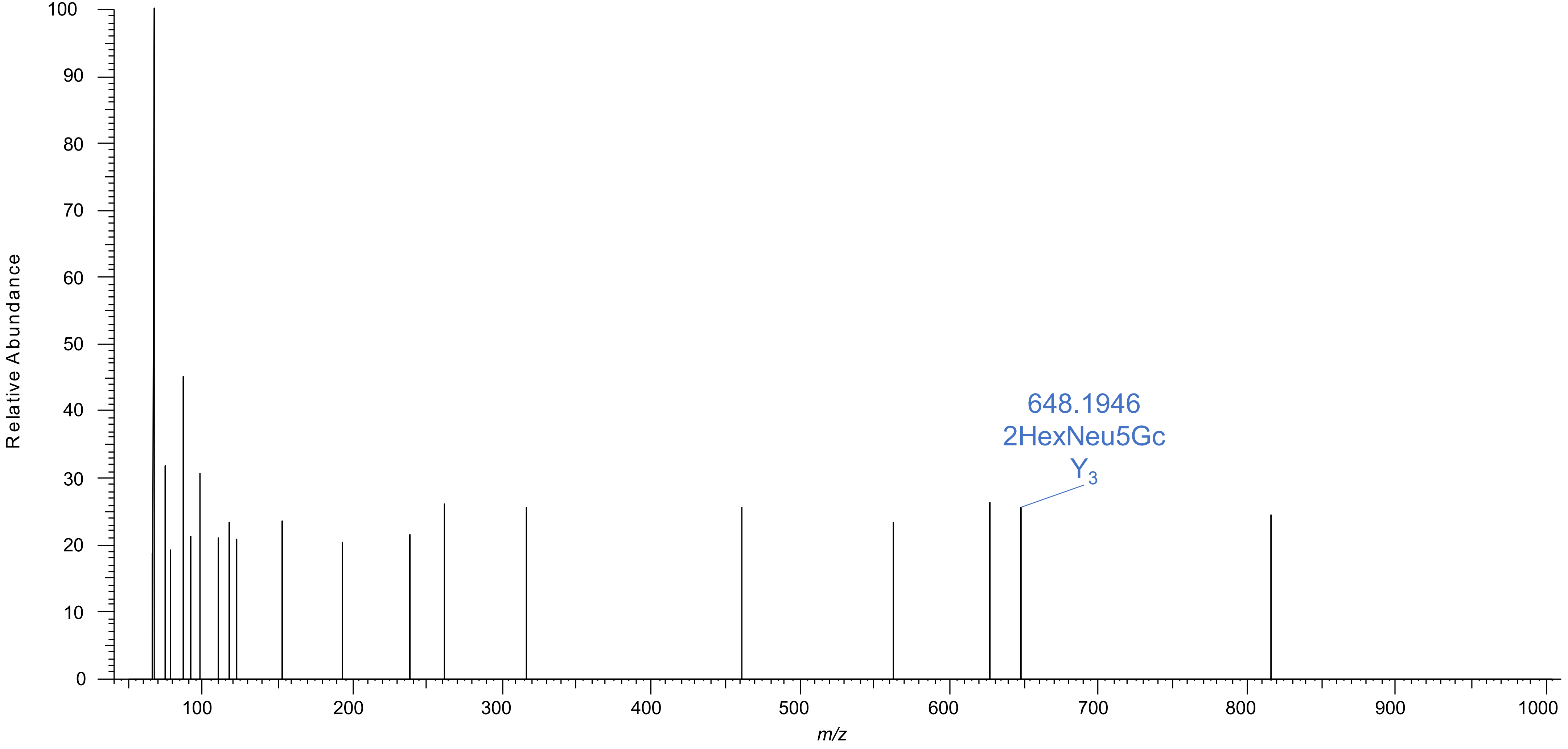

#4L Di-Neu5Gc-lactose (DNGL) lactonized

MS^2 Spectrum RT 9.48 min
No MS^2 Spectrum, see sheep

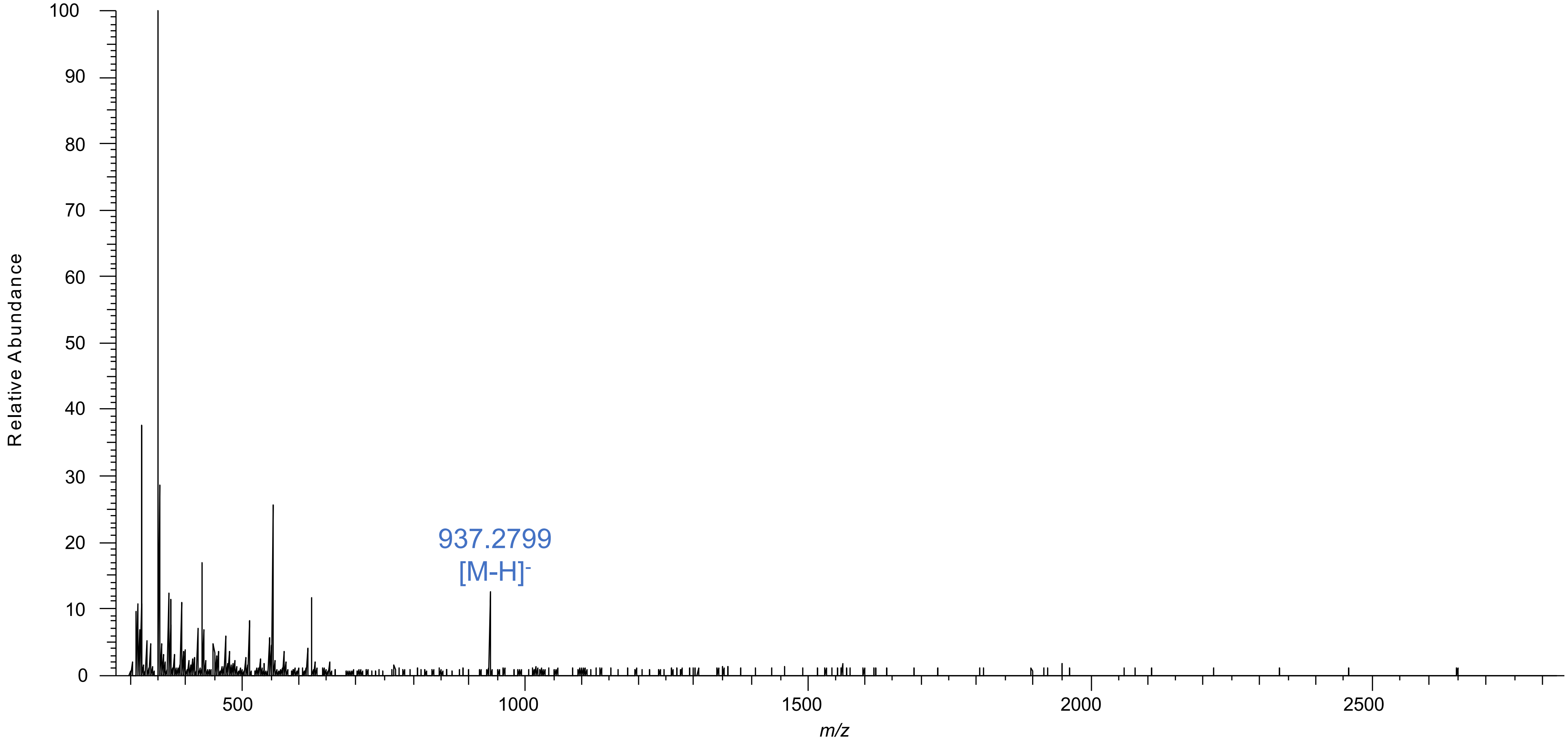

#5a 3‘-Sialyllactose (3‘-SL)

MS^2 Spectrum RT 5.35 min
632.2039 *m/z*

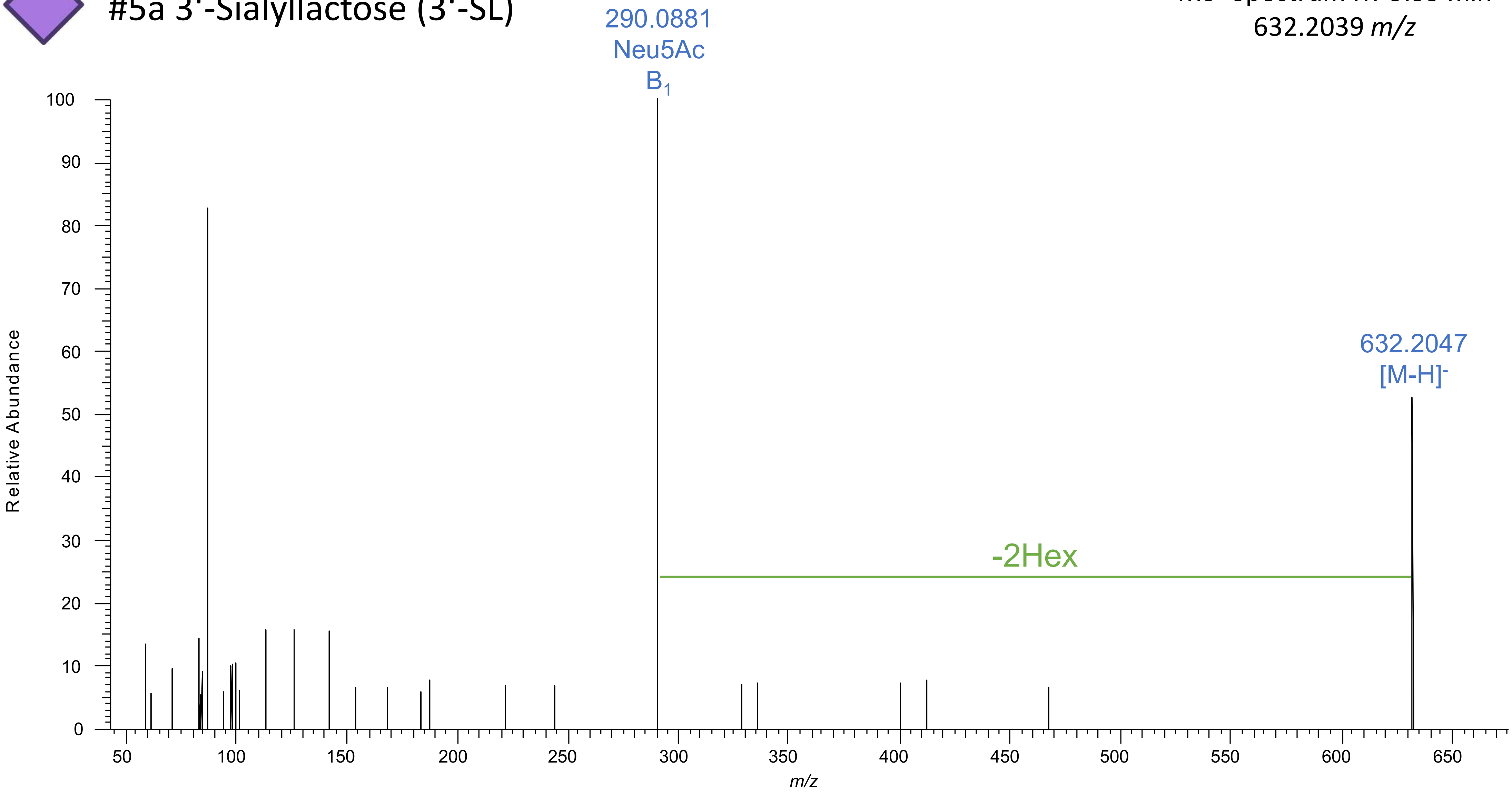

#5b 6‘-Sialyllactose (6‘-SL)

MS^2 Spectrum RT 6.37 min
632.2037 *m/z*

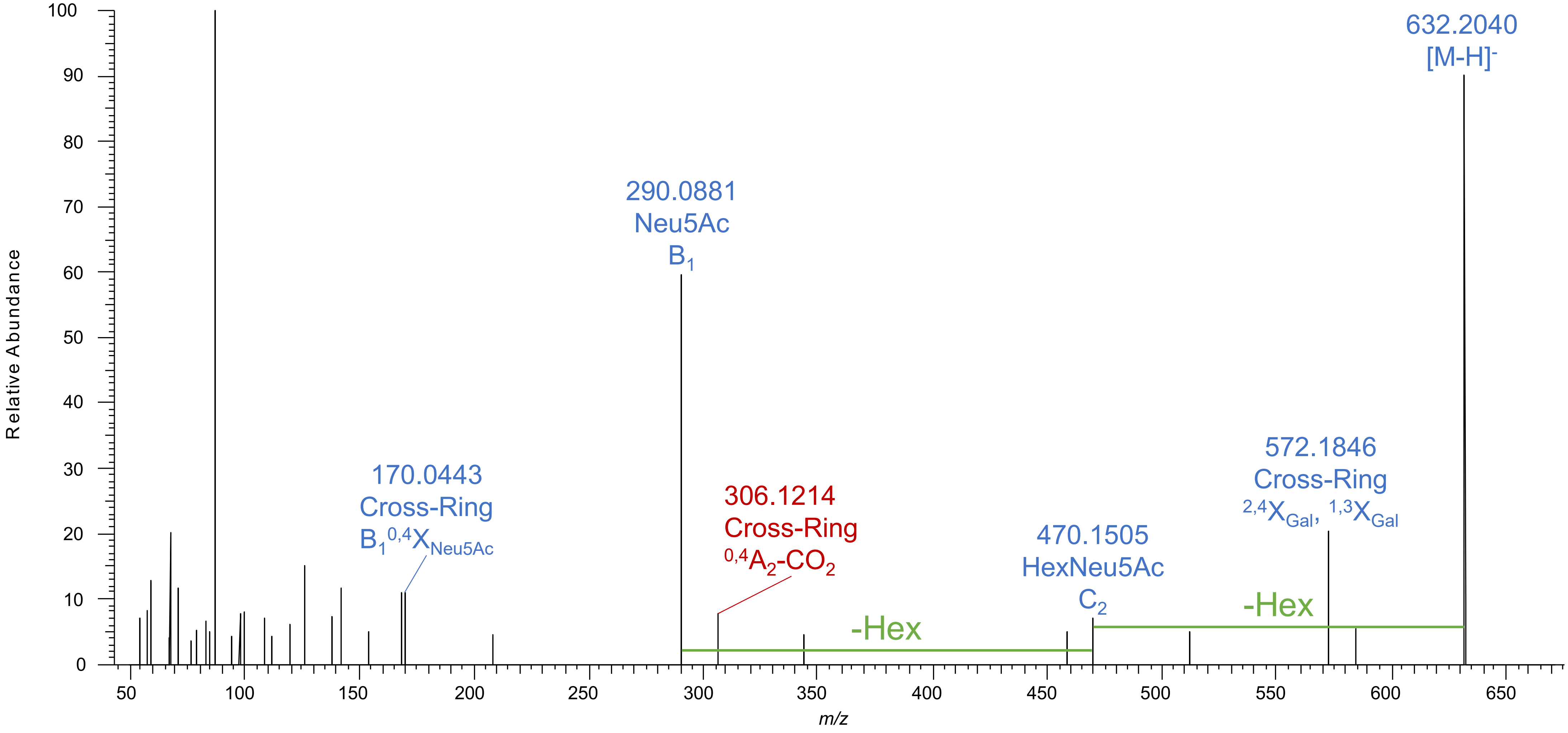

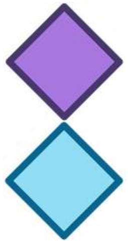

#6 heterogeneous Disialyllactose (hDSL)

proposed order of building blocks: Neu5Ac-Neu5Gc-lactose

MS^2 Spectrum RT 11.07 min
939.2963 *m/z*

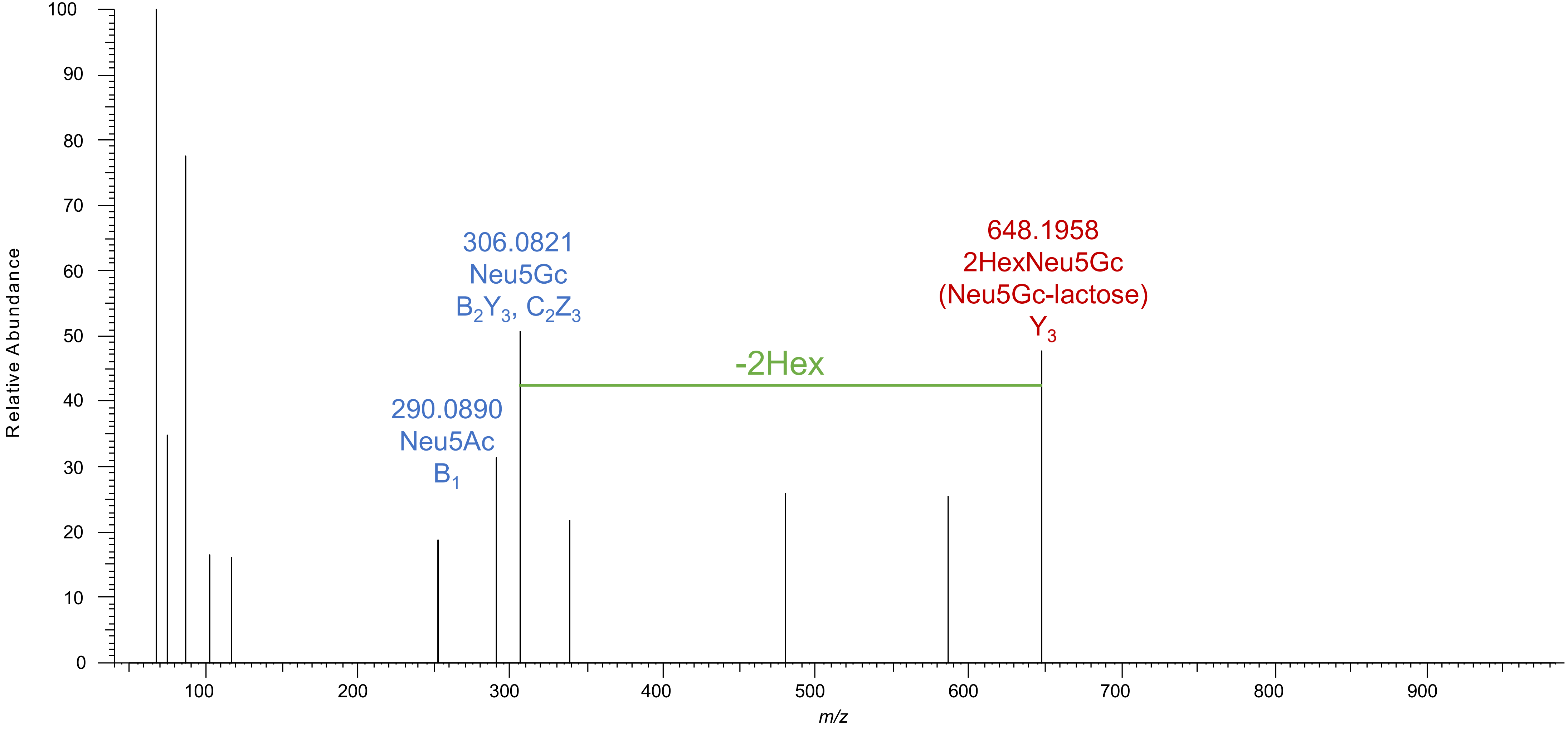

#6L heterogeneous Disialyllactose (hDSL) lactonized

MS^2 Spectrum RT 7.83 min
921.2854 *m/z*

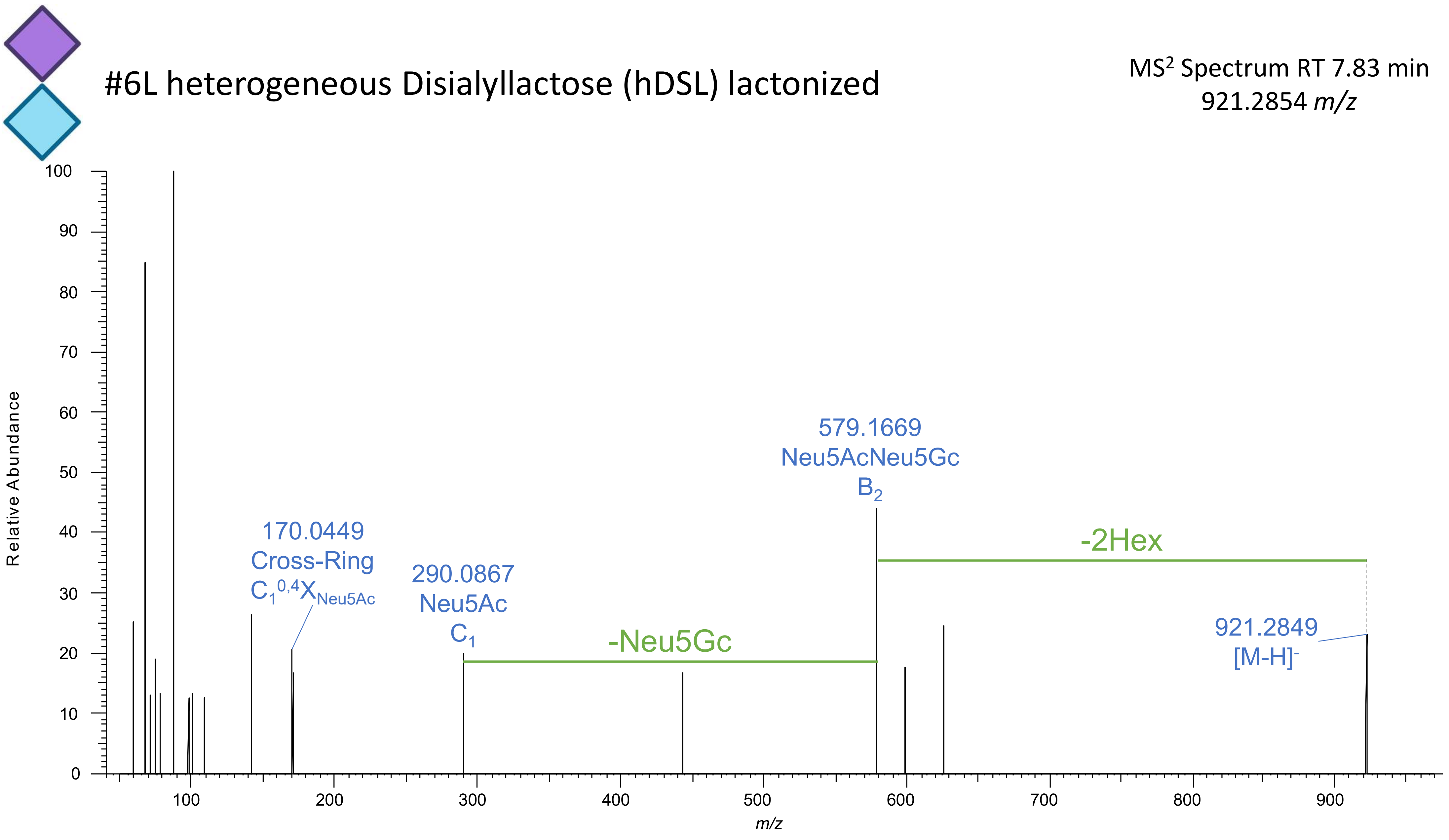

#7 Disialyllactose (DSL)

MS^2 Spectrum RT 10.53 min
923.3015 *m/z*

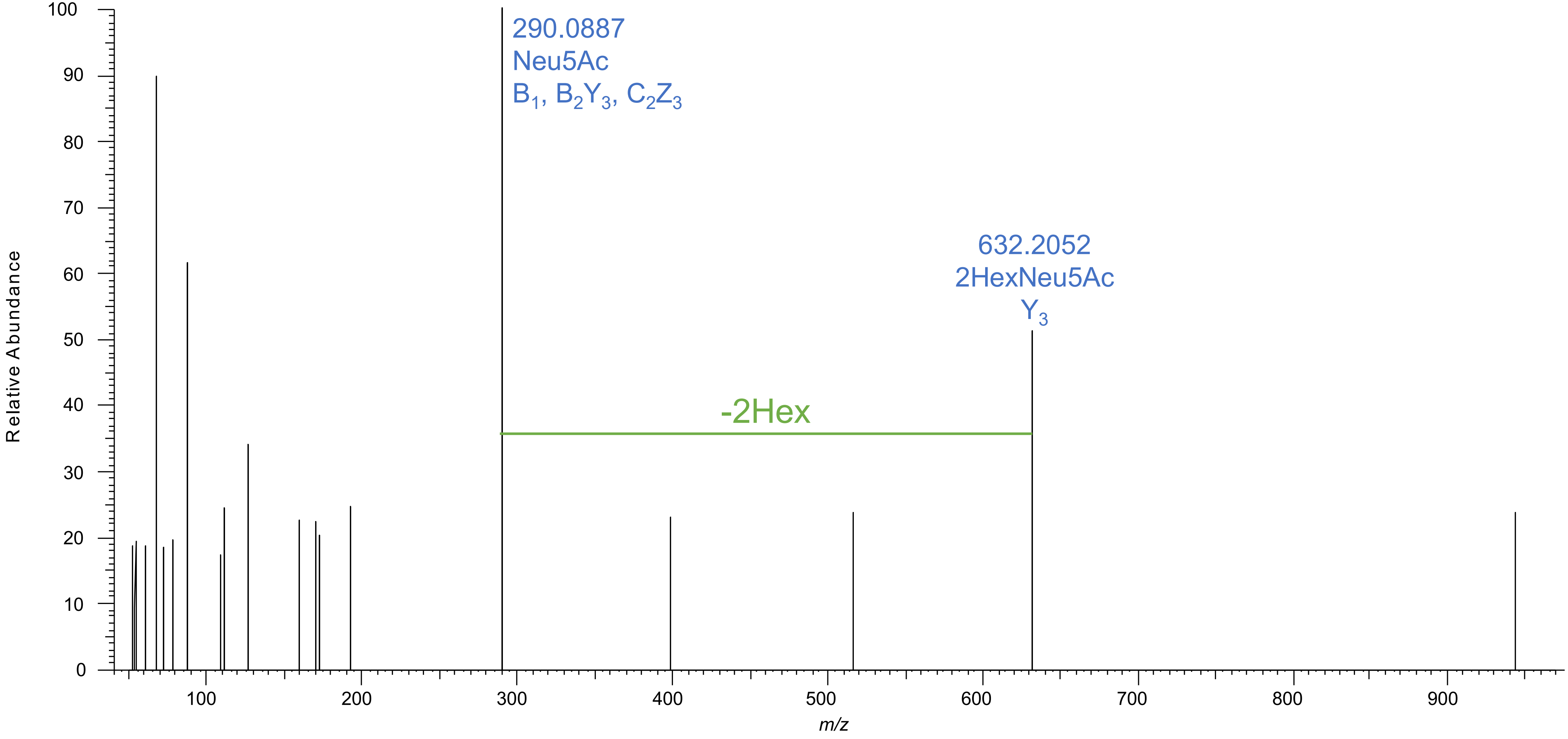

#7L Disialyllactose (DSL) lactonized

MS^2 Spectrum RT 6.68 min
905.2900 *m/z*

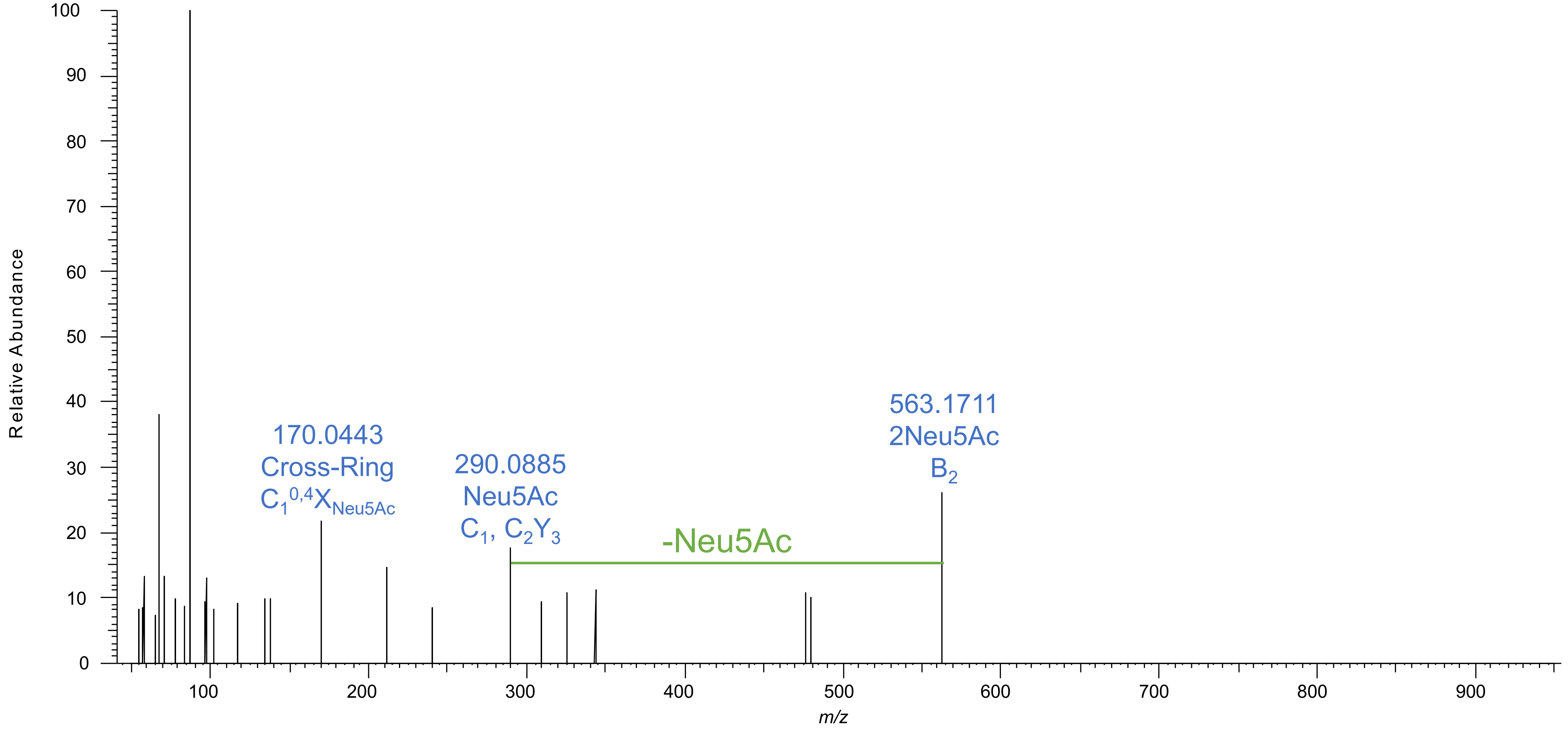

#8 Fucosyllactose (FL)

MS² Spectrum RT 4.01 min
511.1642 *m/z*

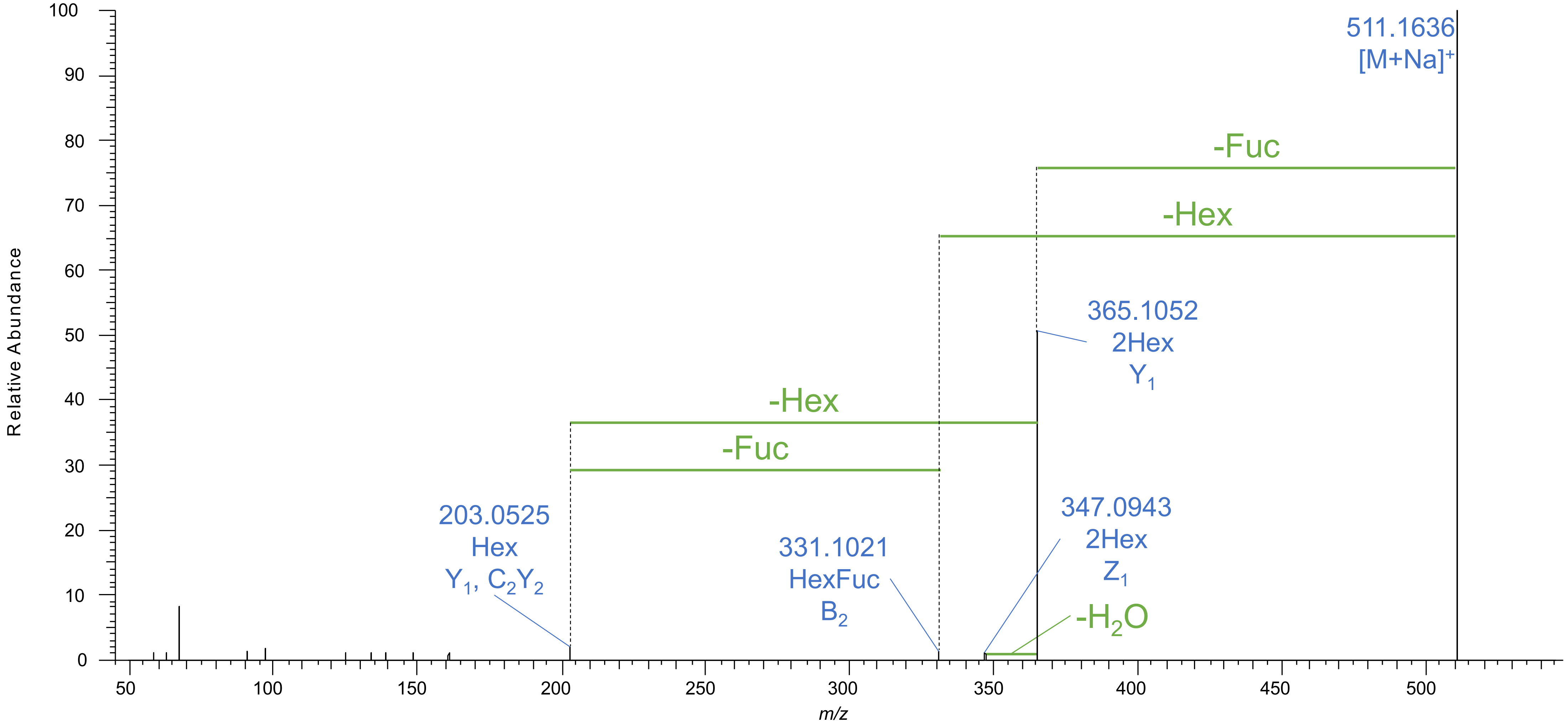

#9a 2_1_0_0_0

MS Spectrum RT: 4.05 min
No MS^2 Spectrum,
RT verified with other samples

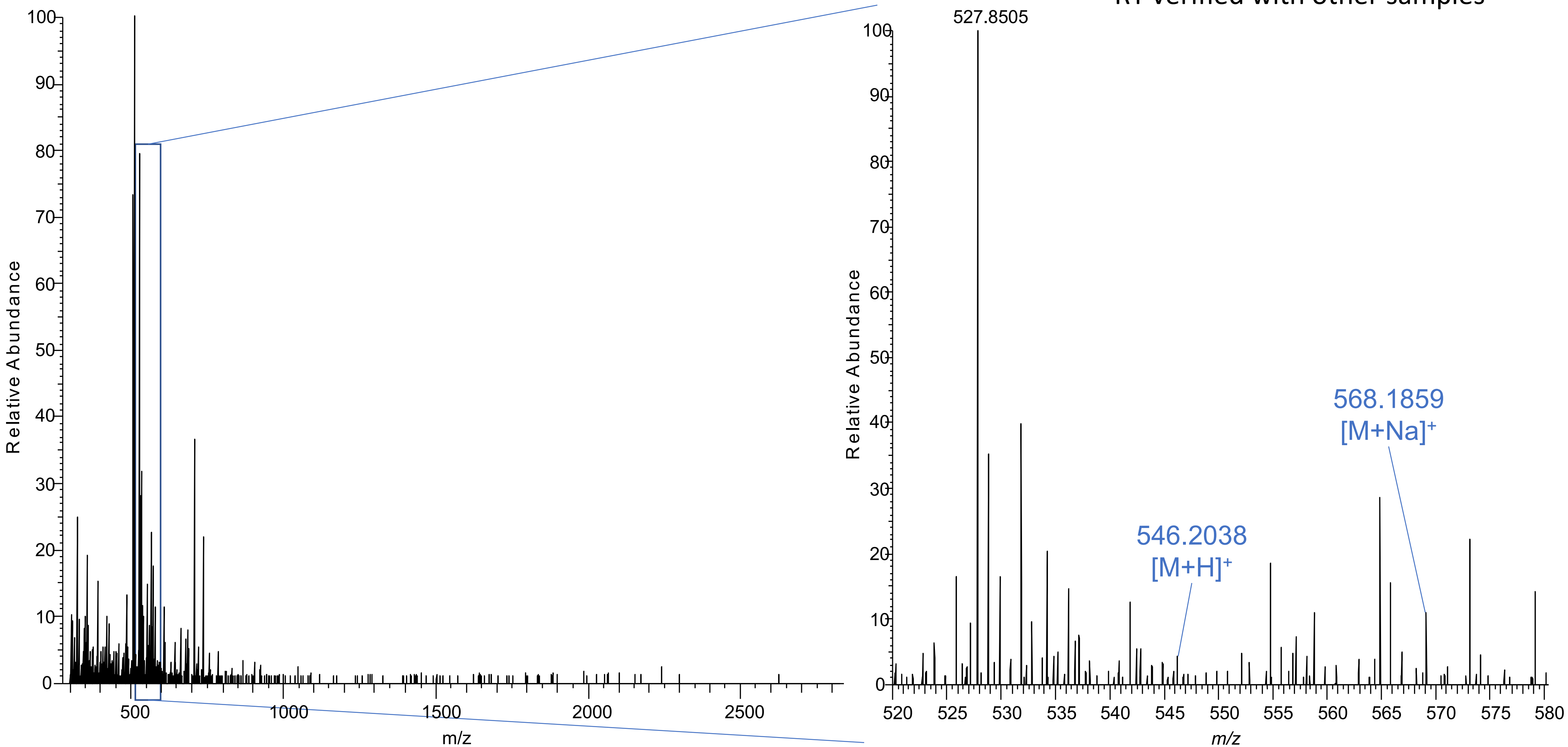

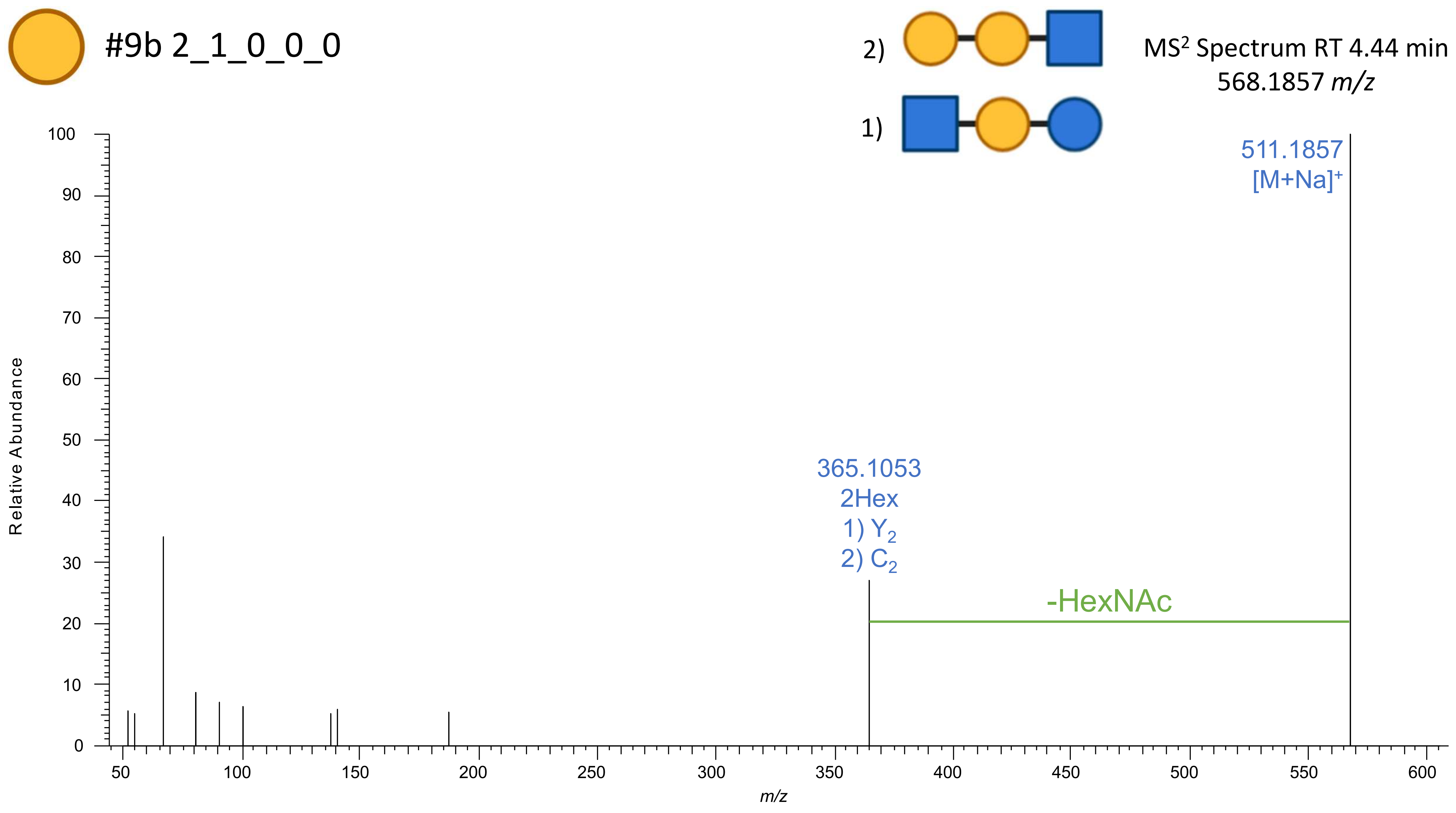
#9b 2_1_0_0_0
2)
1)
MS2 Spectrum RT 4.44 min
568.1857 m/z
511.1857
[M+Na]+
365.1053
2Hex
1) Y2
2) C2
-HexNAc
Relative Abundance
m/z
0
10
20
30
40
50
60
70
80
90
100
50
100
150
200
250
300
350
400
450
500
550
600

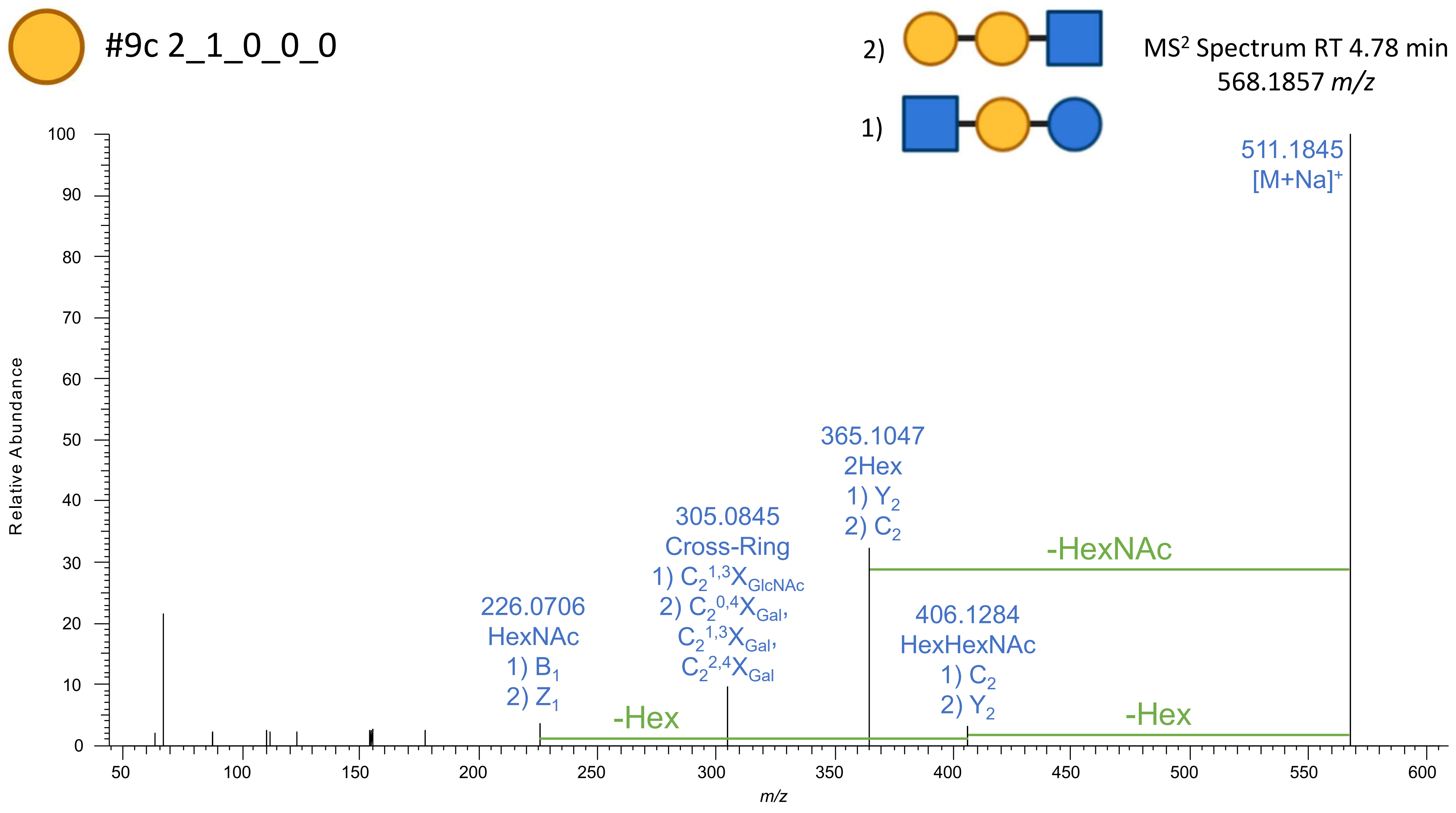

#9c 2_1_0_0_0
2)
1)
MS2 Spectrum RT 4.78 min
568.1857 m/z
511.1845
[M+Na]+
365.1047
2Hex
1) Y2
2) C2
305.0845
Cross-Ring
1) C2 1,3X GlcNAc
2) C2 0,4X Gal,
C2 1,3X Gal,
C2 2,4X Gal
226.0706
HexNAc
1) B1
2) Z1
406.1284
HexHexNAc
1) C2
2) Y2
-HexNAc
-Hex
-Hex
Relative Abundance
m/z

#10a 2_1_0_1_0

MS Spectrum RT: 6.92 min
No MS^2 Spectrum,
RT verified with other samples

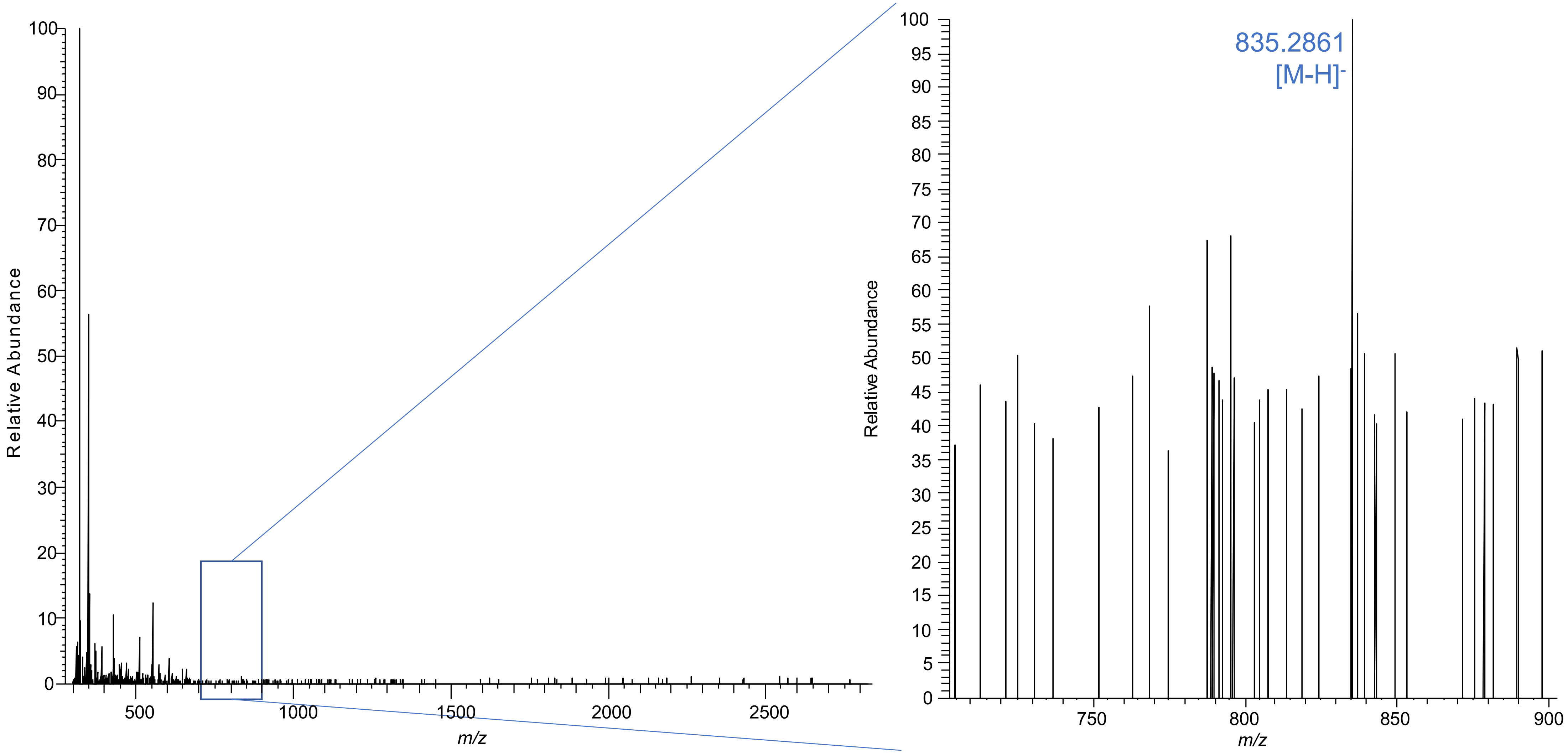

#10b 2_1_0_1_0

MS Spectrum RT: 7.51 min
No MS^2 Spectrum,
RT verified with other samples

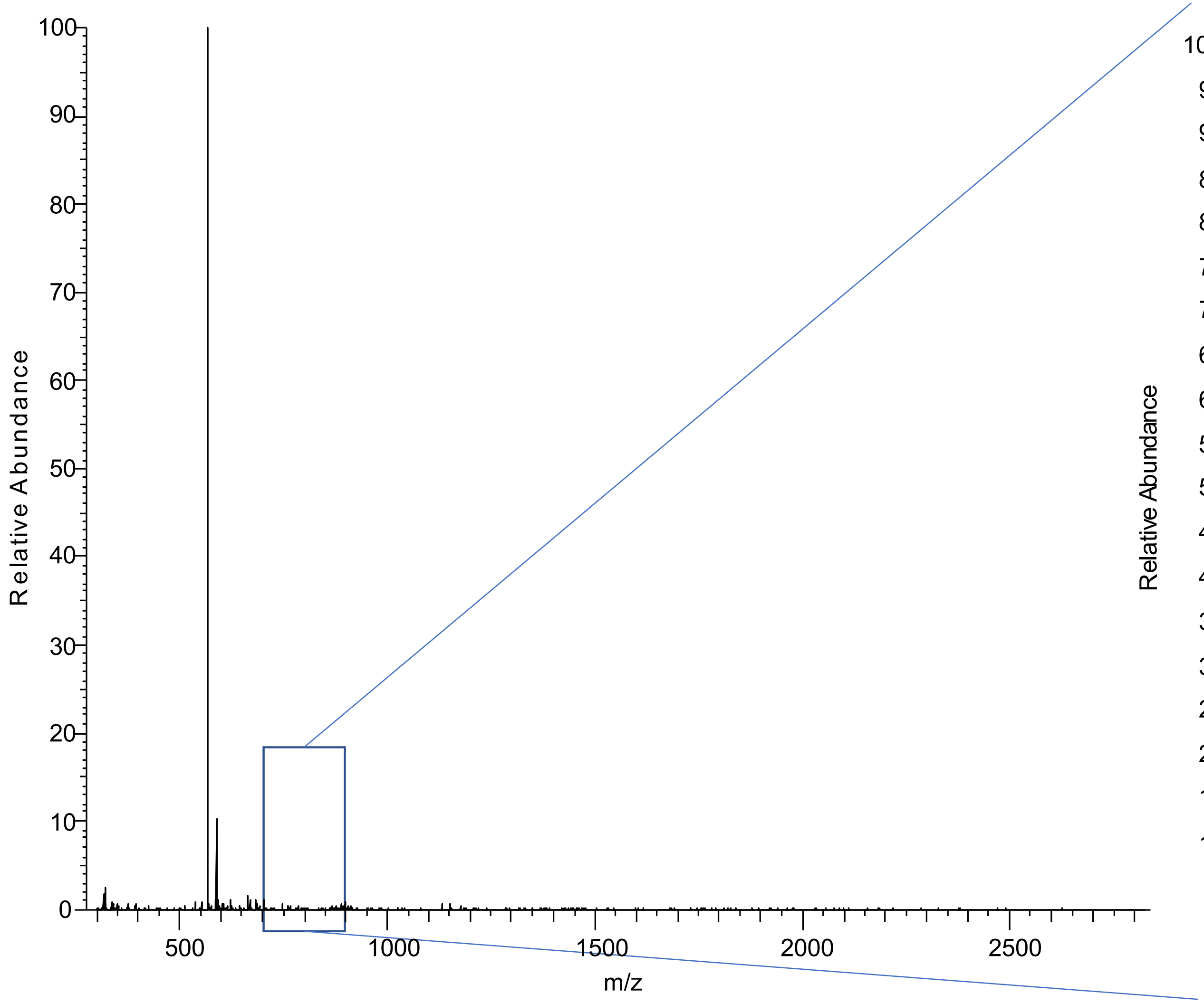

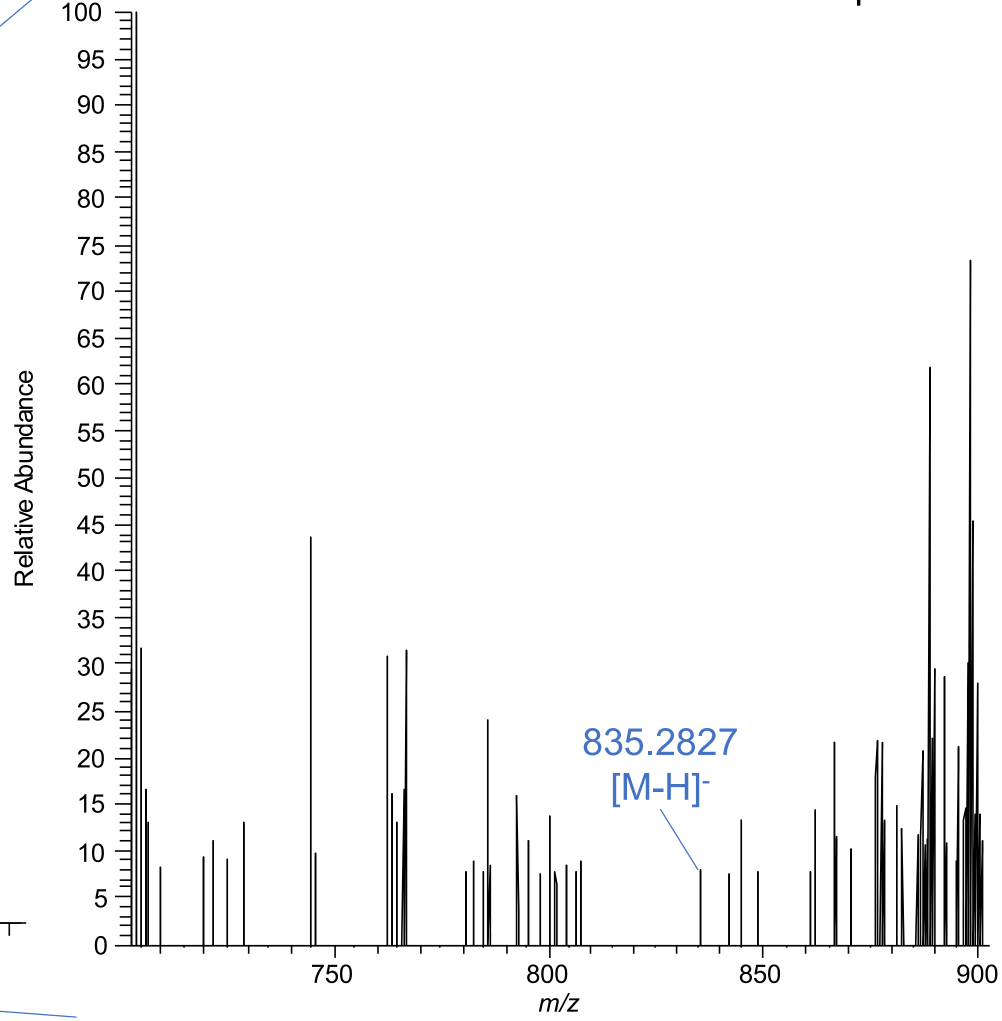

#10c 2_1_0_1_0

MS Spectrum RT: 8.13 min
No MS^2 Spectrum,
RT verified with other samples

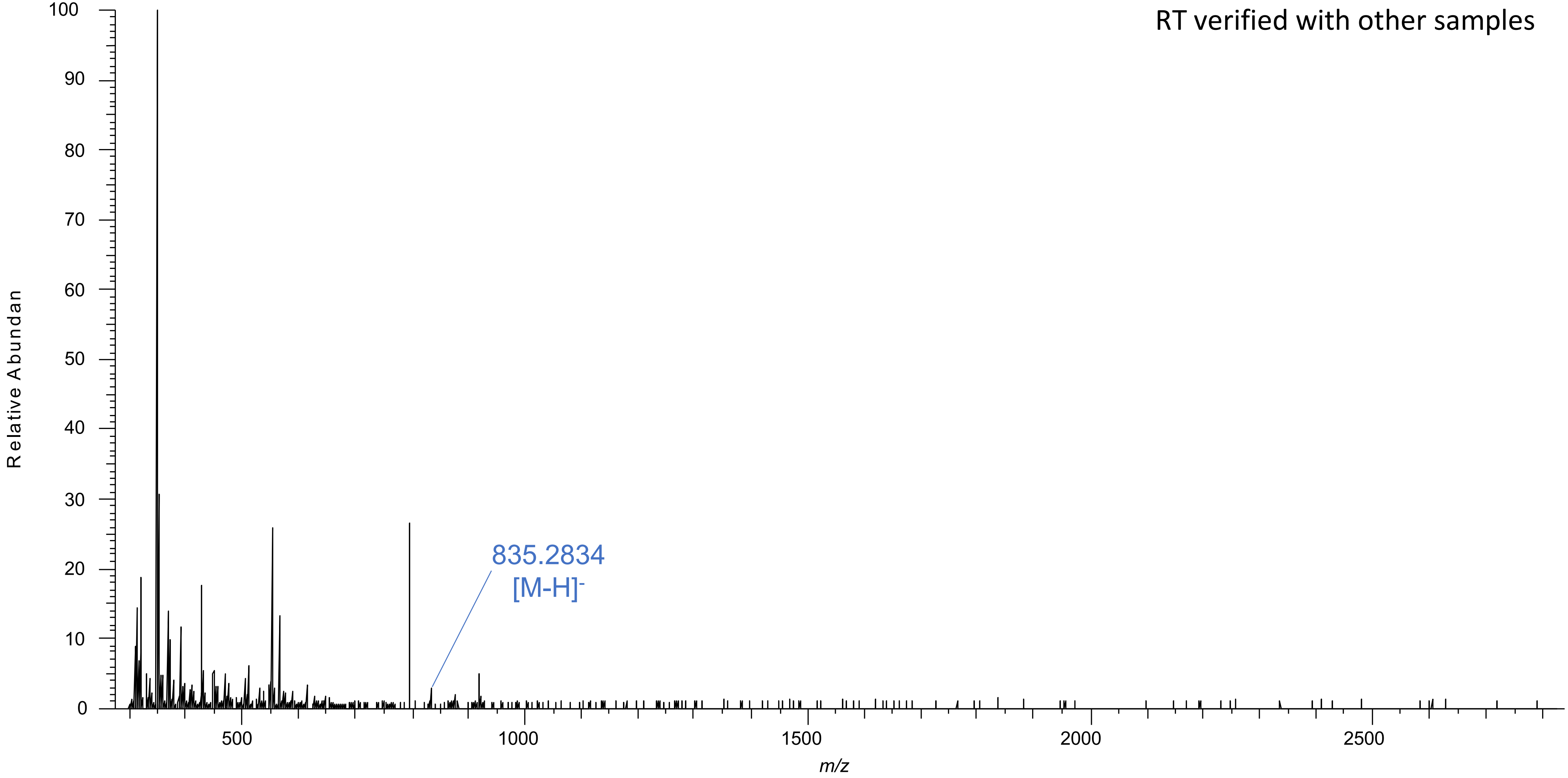

#11 2_1_1_0_0

MS Spectrum RT: 5.72 min
No MS^2 Spectrum,
RT verified with other samples

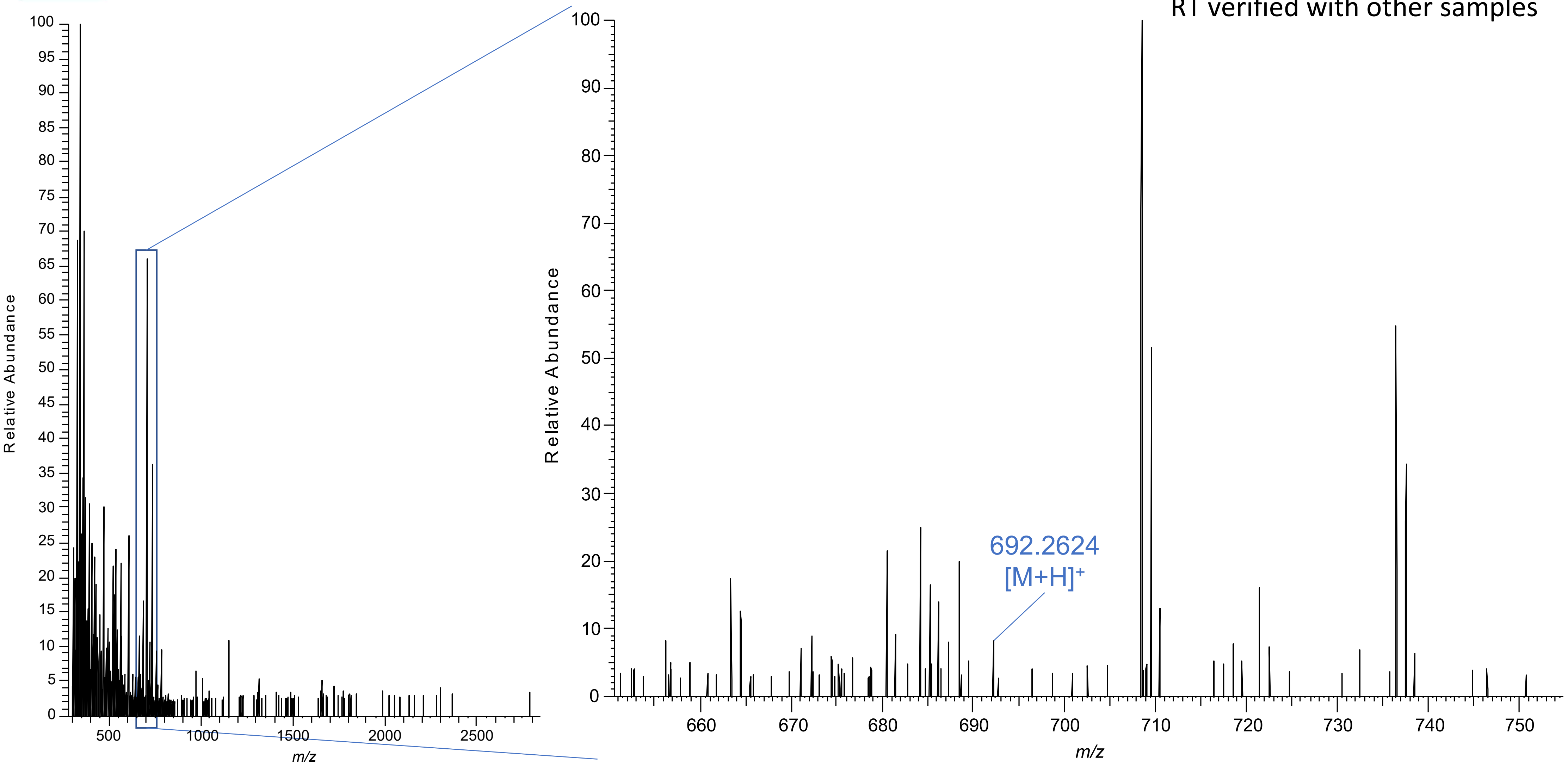

#12a Galactosyllactose (GL)

MS^2 Spectrum RT 5.47 min
527.1583 *m/z*

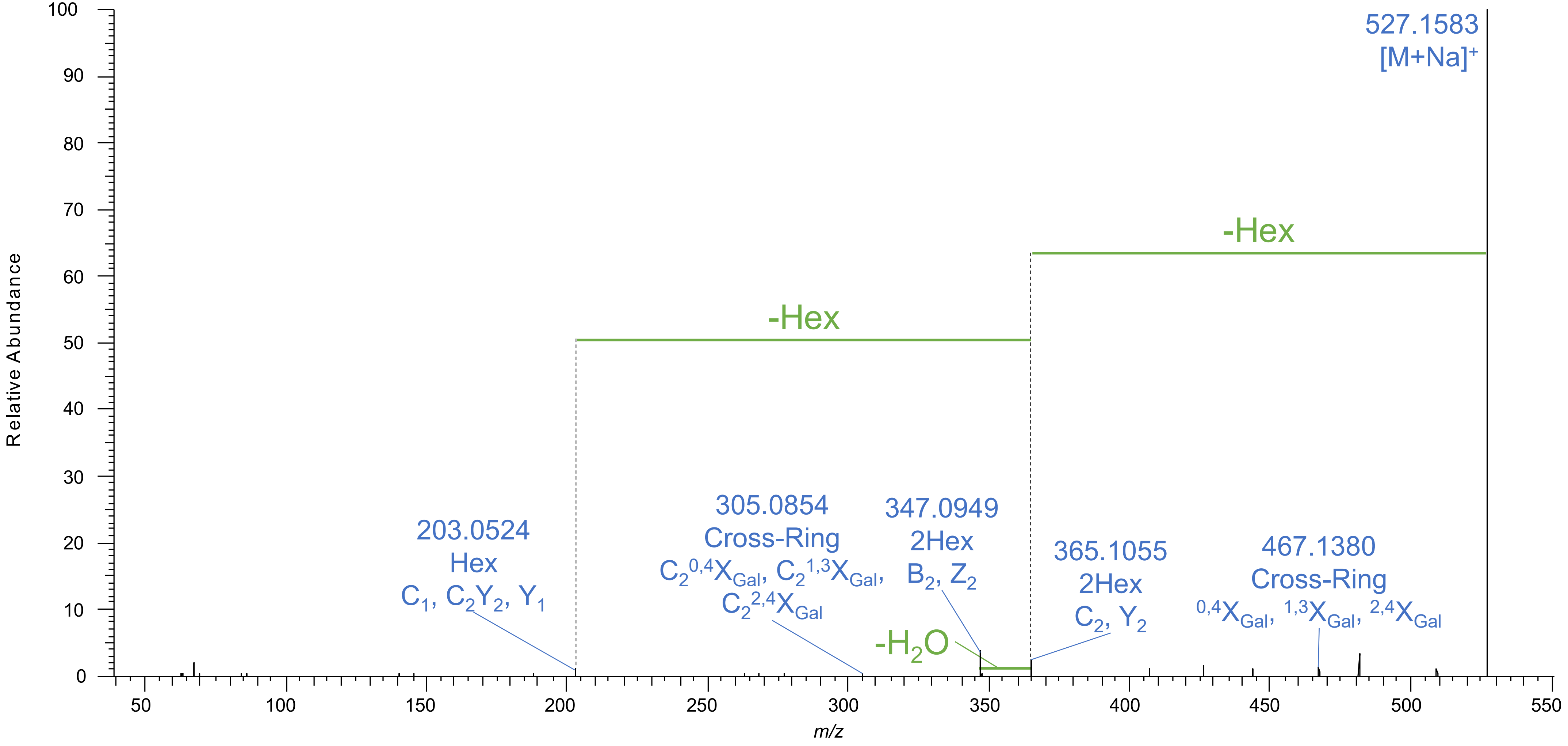

#12b Galactosyllactose (GL)

MS² Spectrum RT 5.7 min89
527.1577 *m/z*

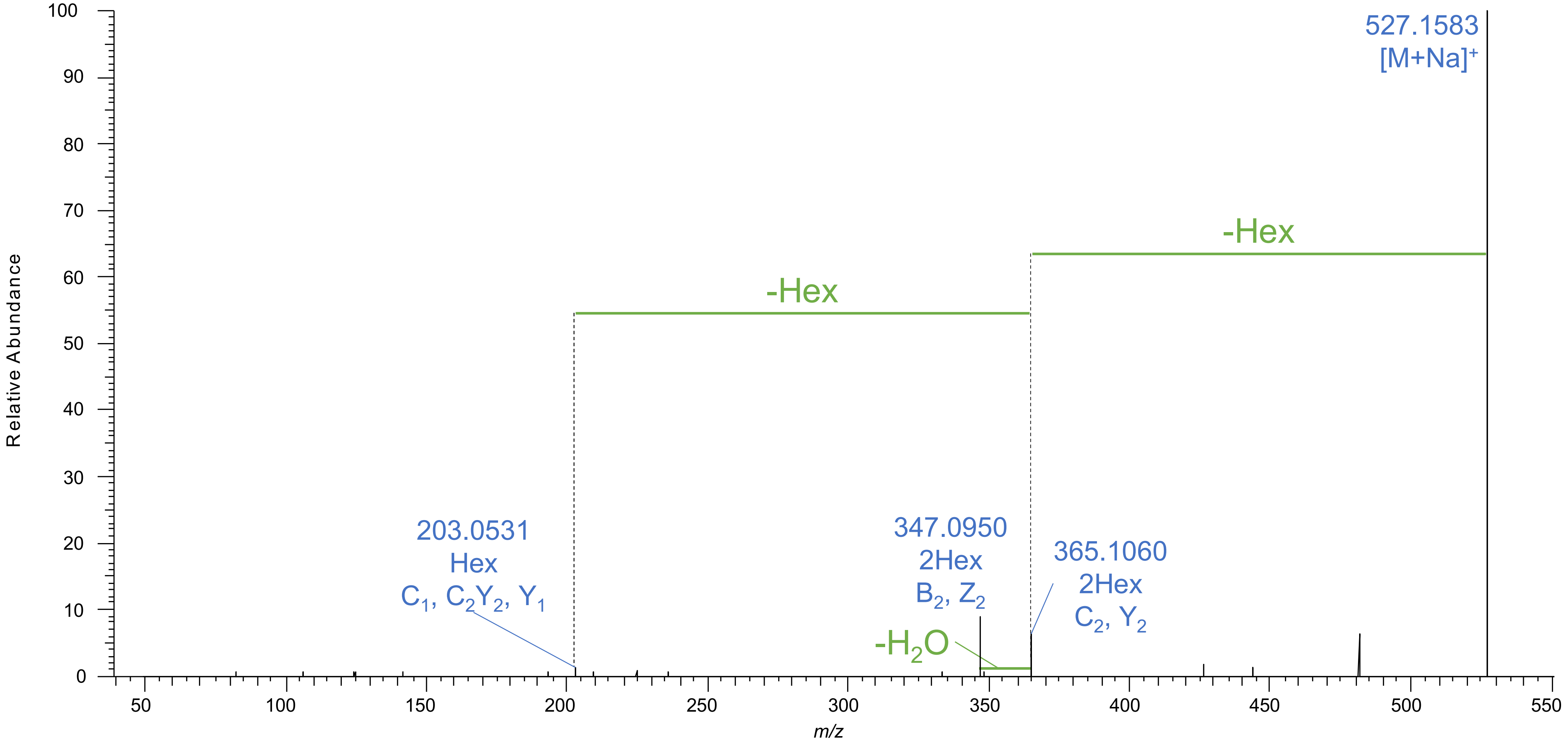

#13a Neu5Gc-GL (NG-GL)

MS^2 Spectrum RT 9.86 min
810.2527 *m/z*

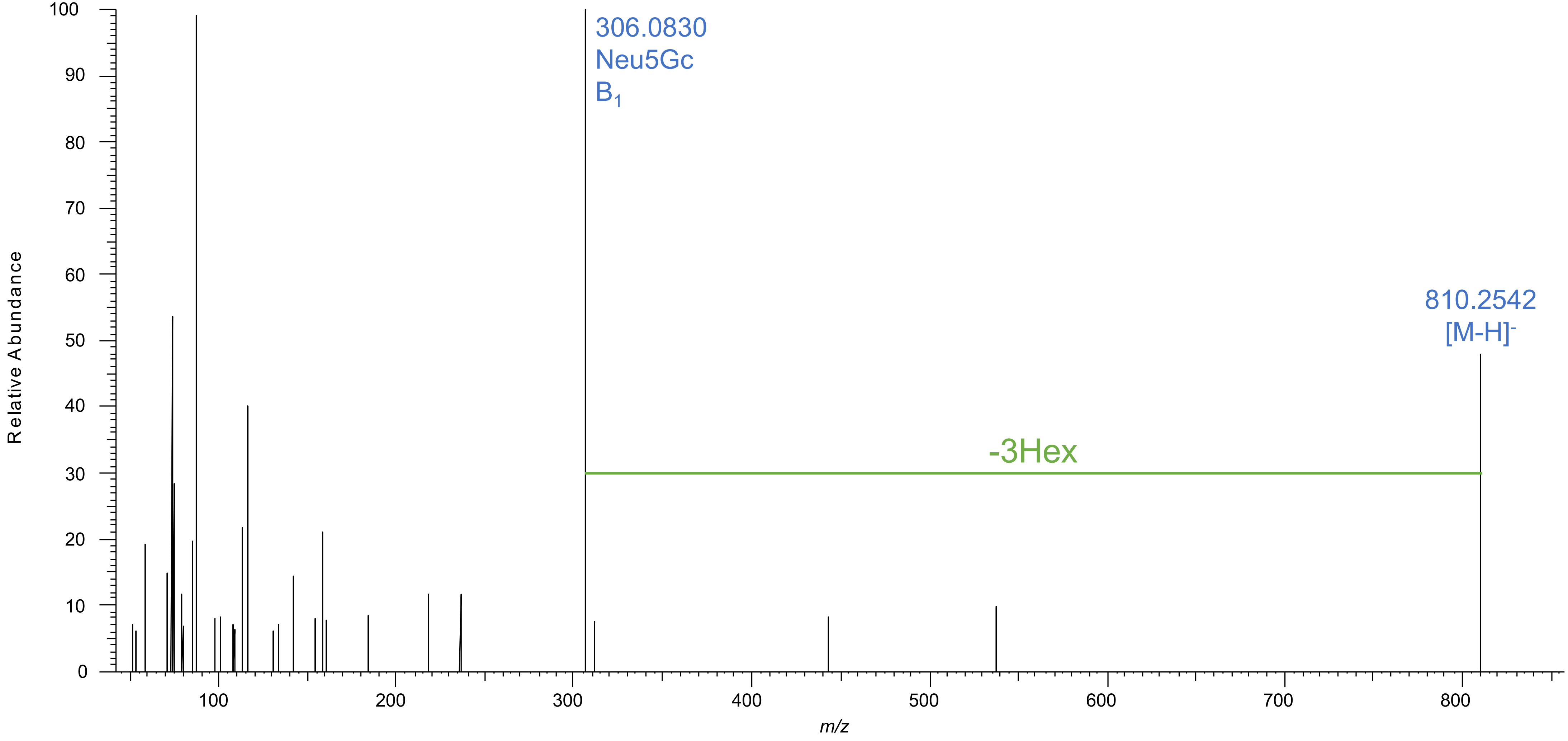

#13b Neu5Gc-GL (NG-GL)

MS^2 Spectrum RT 10.50 min
810.2525 *m/z*

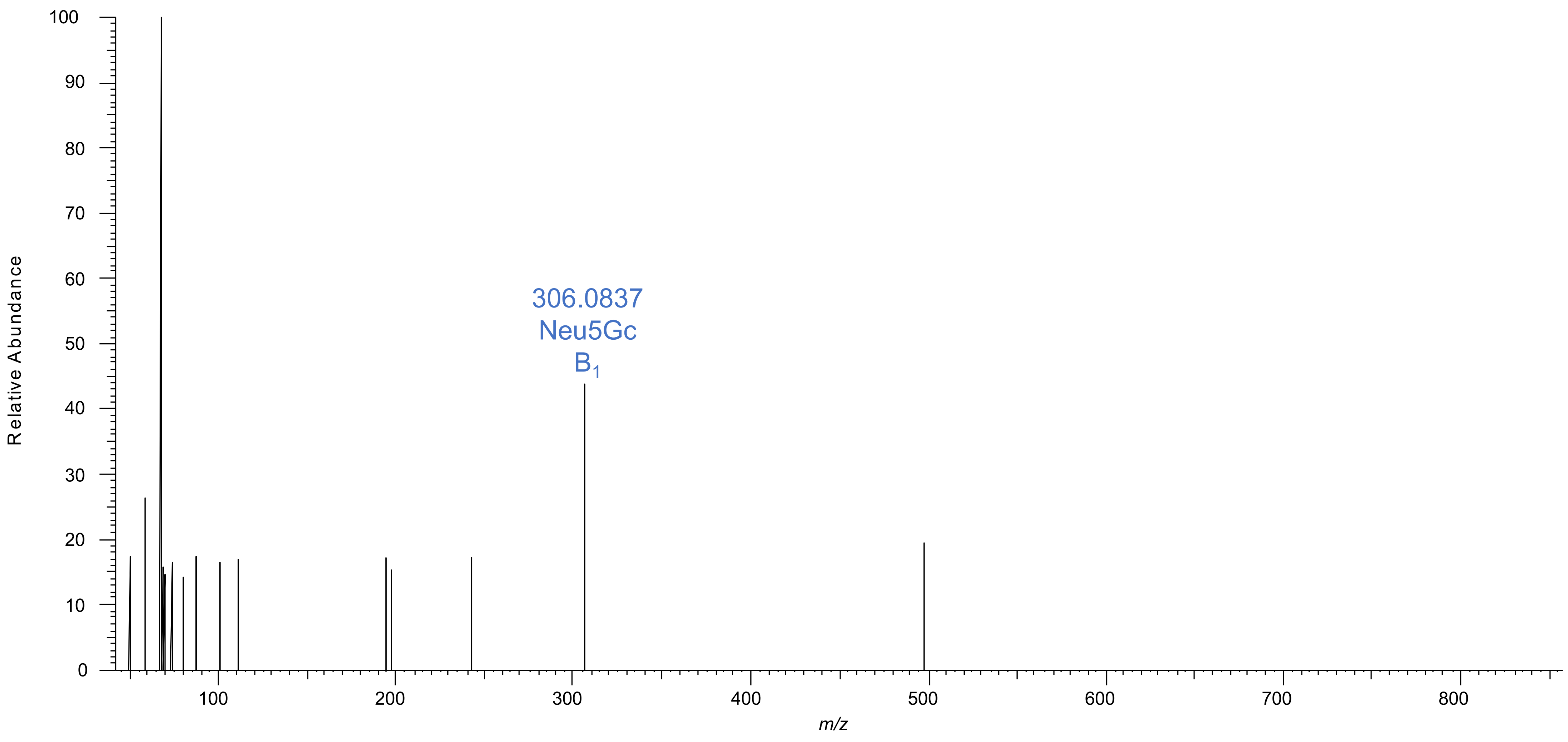

#14 Neu5Gc-Disialyl-triose

MS² Spectrum RT 15.96 min
558.1673 *m/z*

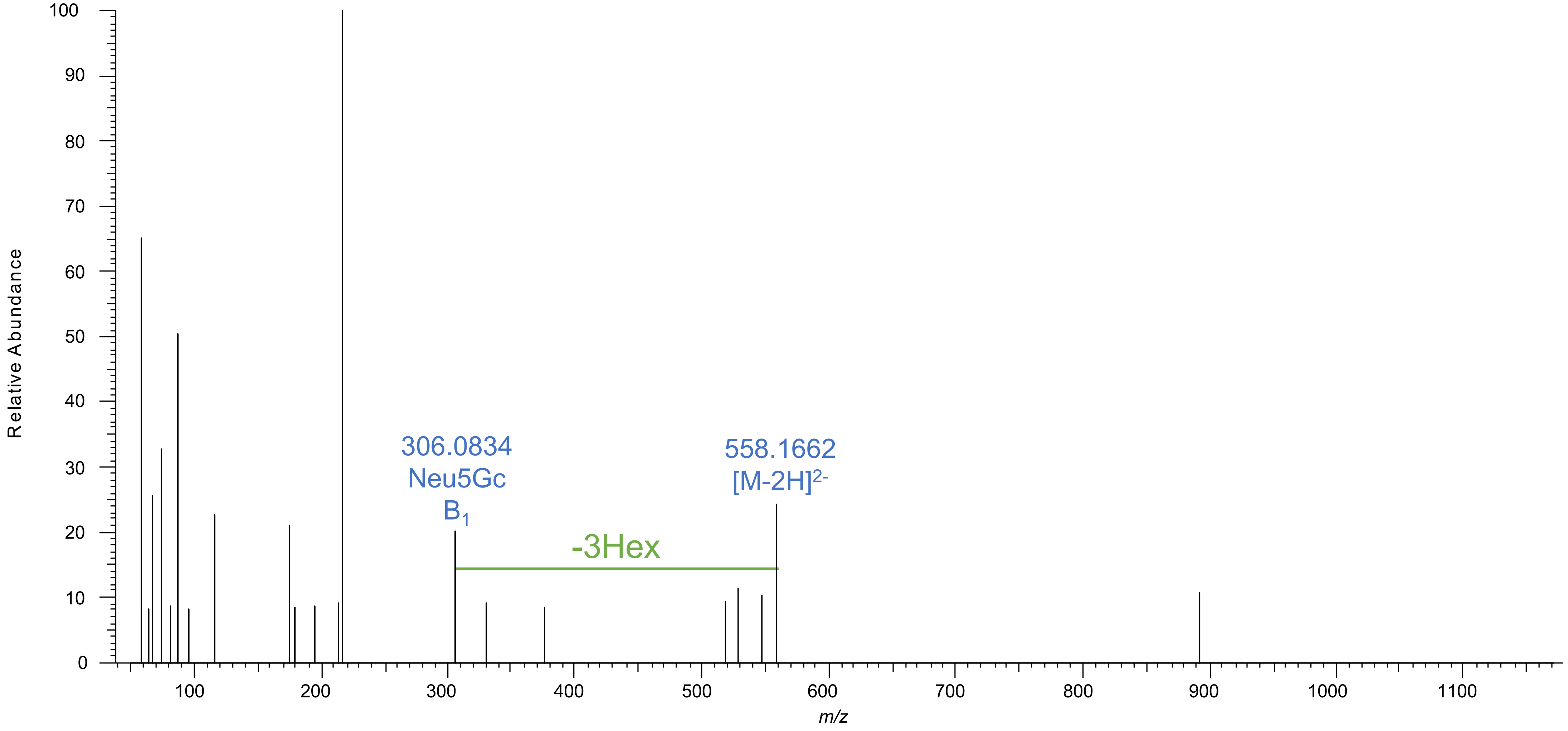

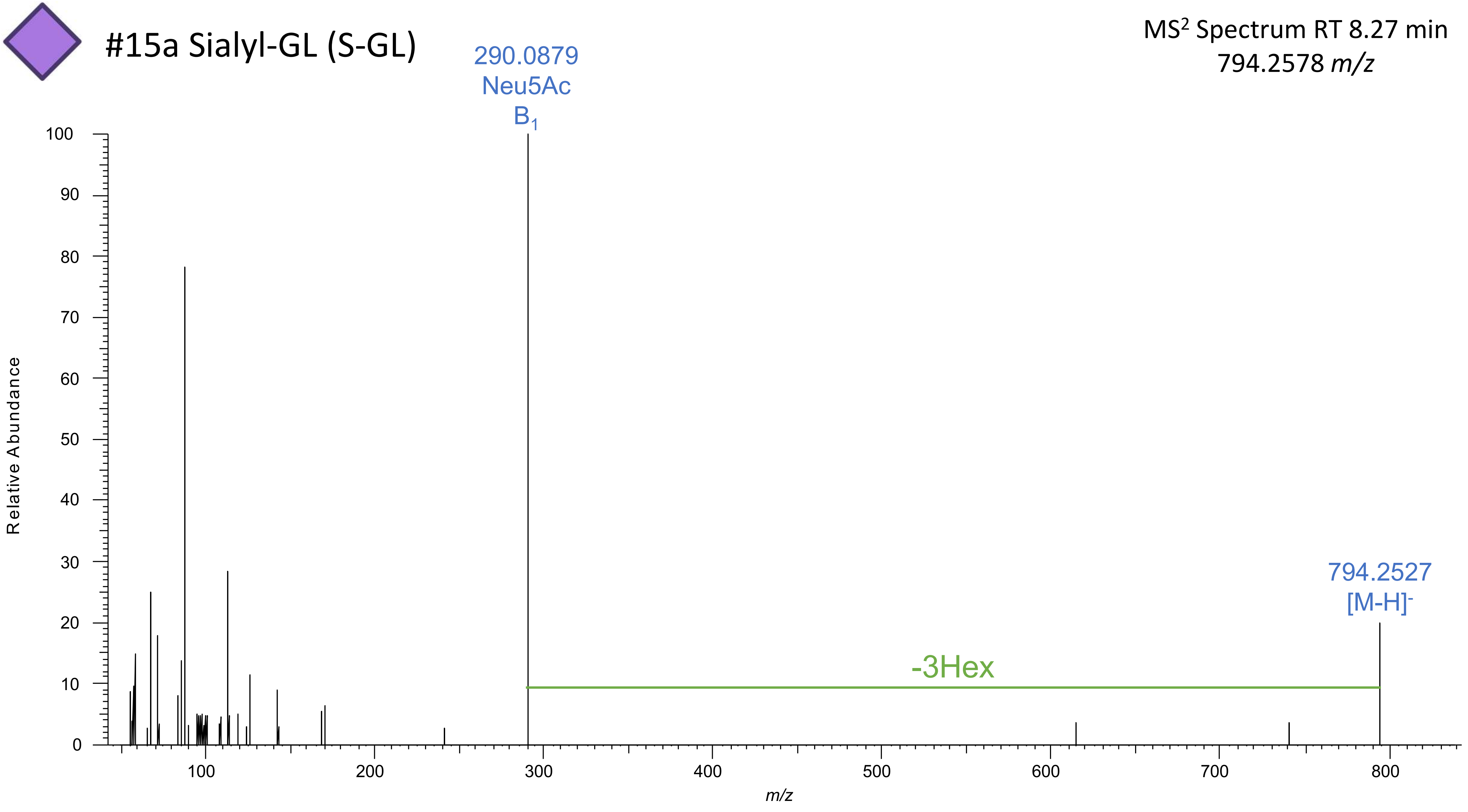
#15a Sialyl-GL (S-GL)
MS² Spectrum RT 8.27 min
794.2578 m/z
290.0879
Neu5Ac
B1
794.2527
[M-H]-
-3Hex
Relative Abundance
m/z
0
10
20
30
40
50
60
70
80
90
100
200
300
400
500
600
700
800

#15b Sialyl-GL (S-GL)

MS^2 Spectrum RT 8.86 min
794.2583 *m/z*

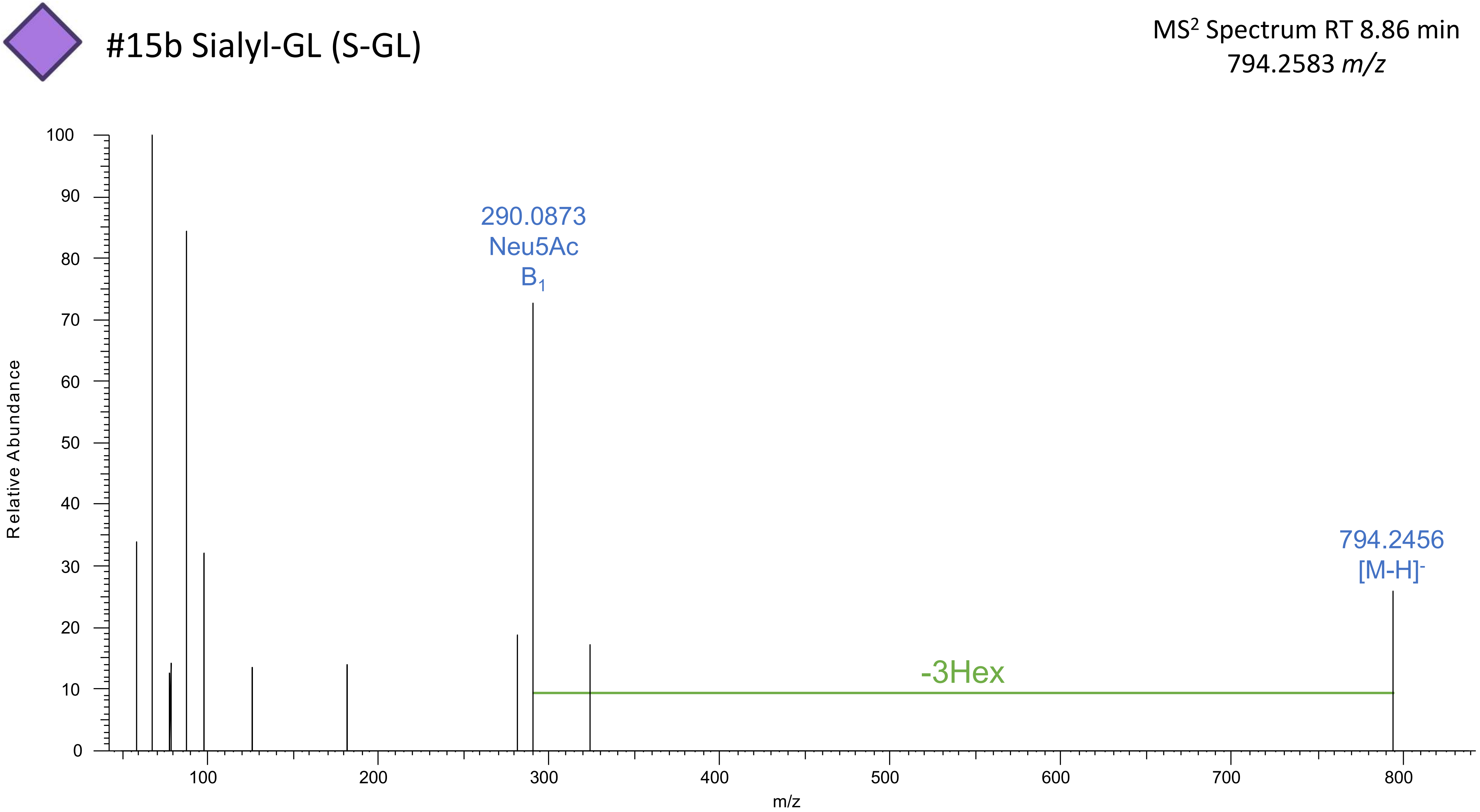

#15c Sialyl-GL (S-GL)

MS Spectrum RT: 9.21 min
No MS^2 Spectrum,
RT verified with other samples

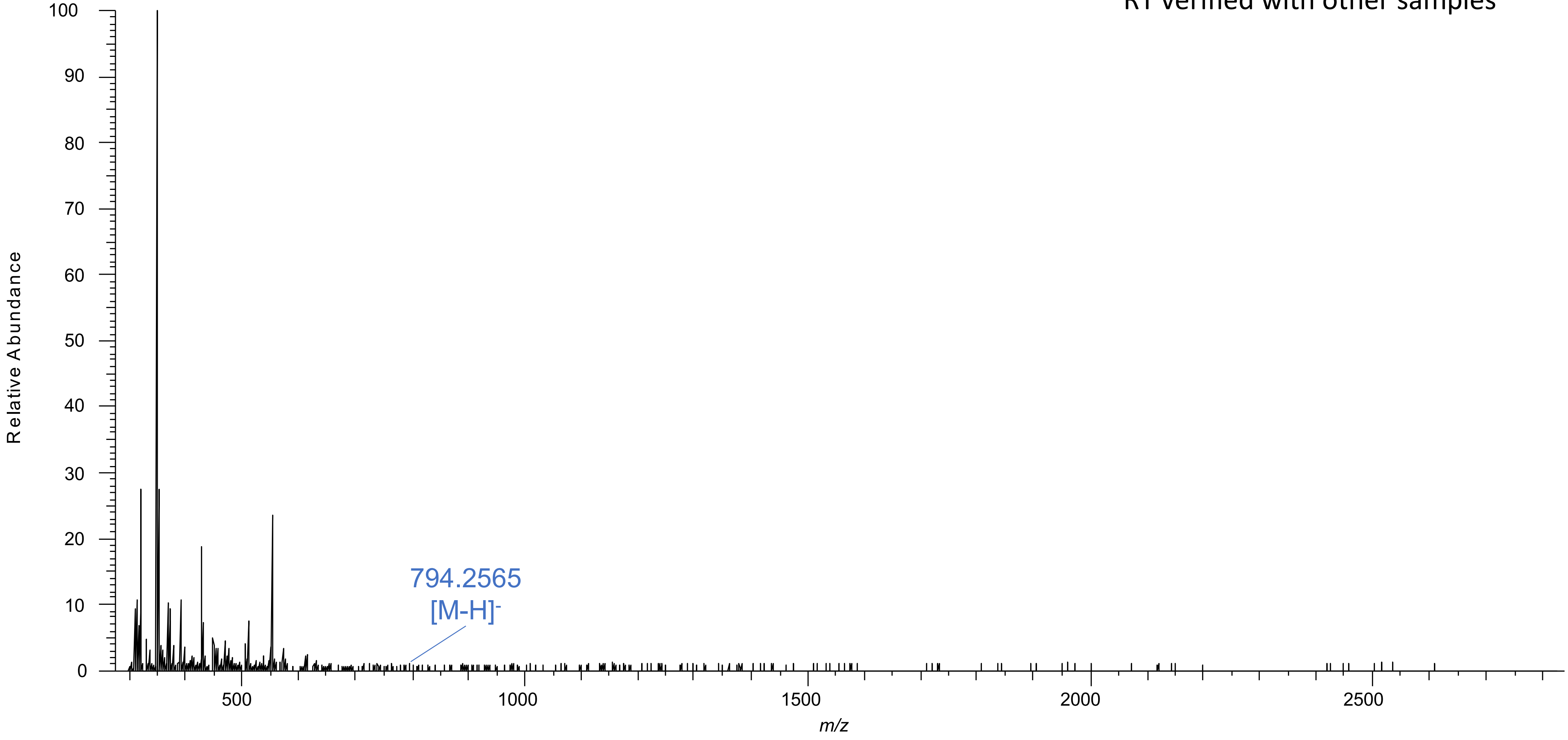

#16 heterogeneous Disialyl-triose

MS2 Spectrum RT 14.31 min
550.1704 *m/z*

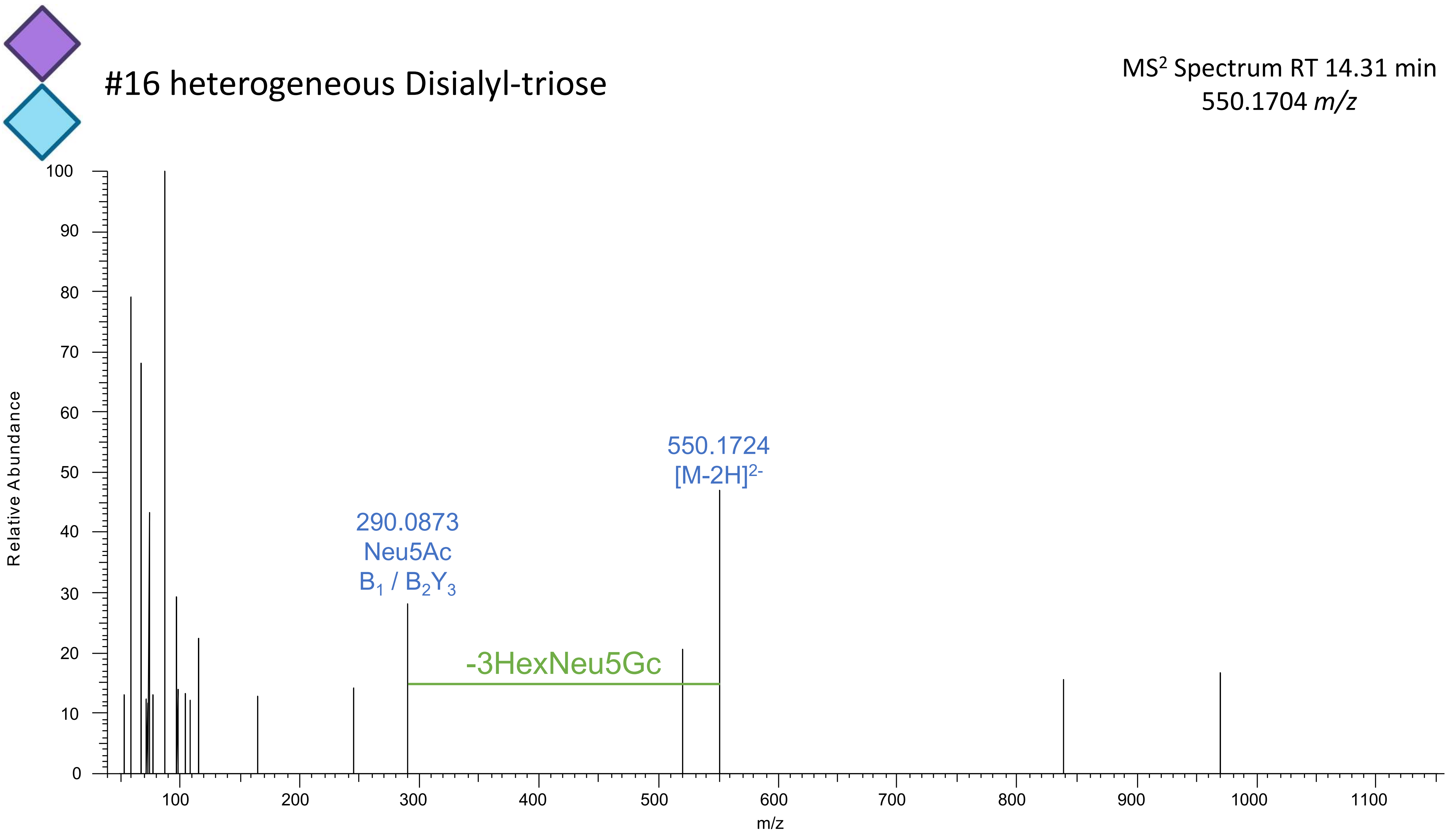

#17 Disialyl-triose

MS^2 Spectrum RT 12.69 min
542.1725 *m/z*

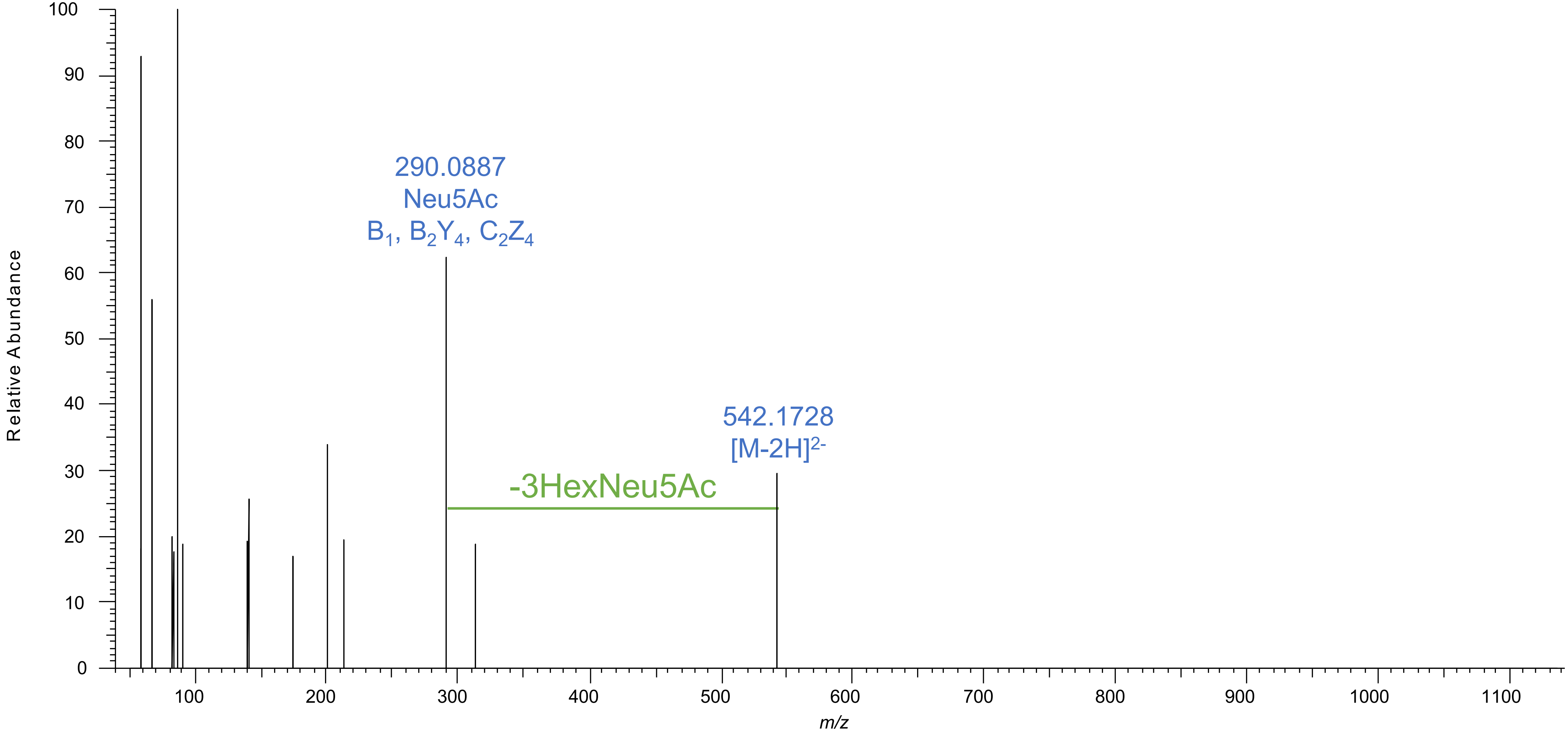

#18a Lacto-N(-neo)-tetraose (LN(n)T)

MS Spectrum RT: 7.28 min
No MS^2 Spectrum,
RT verified with other samples

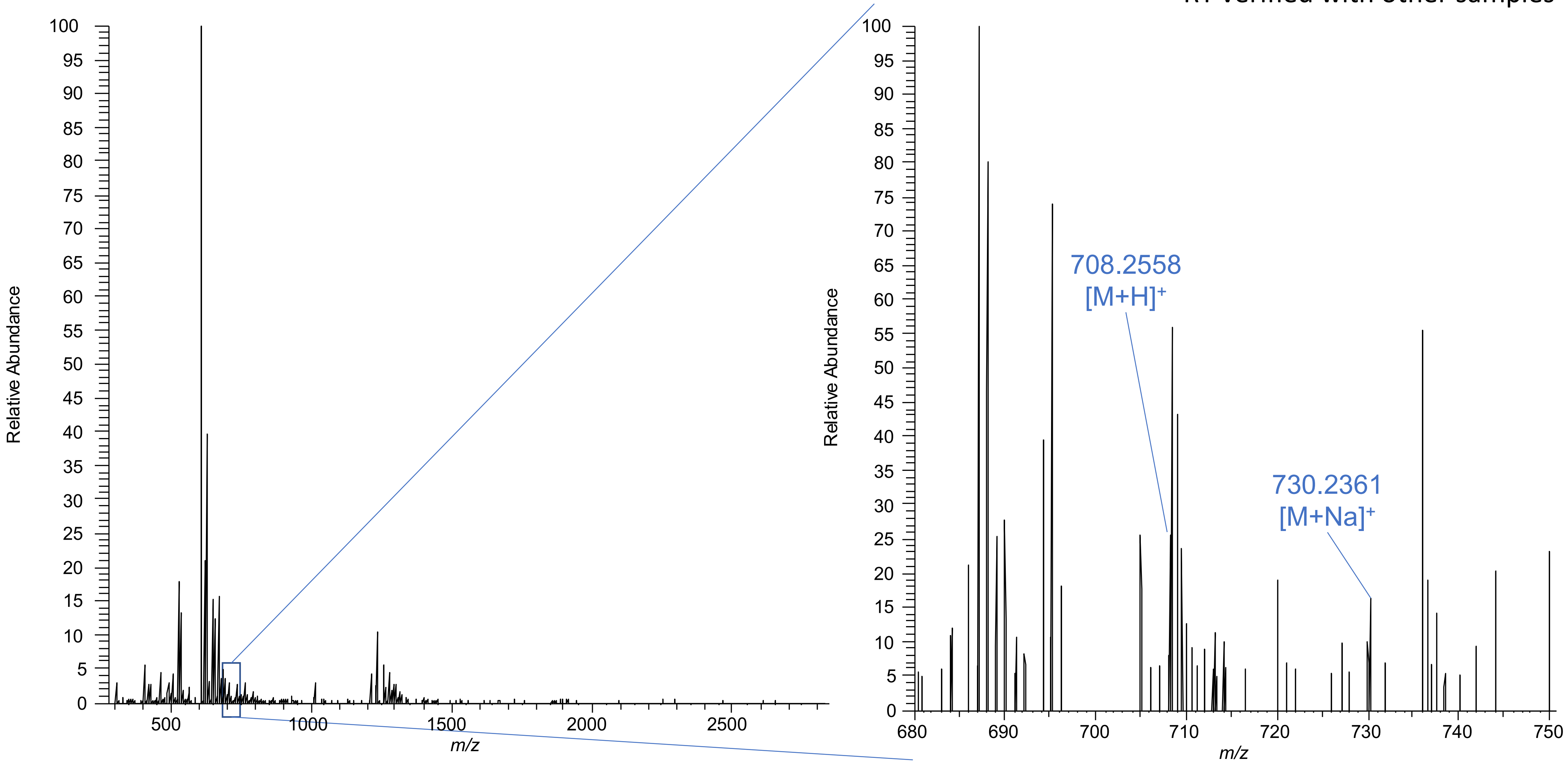

#18b Lacto-N(-neo)-tetraose (LN(n)T)

MS Spectrum RT: 7.43 min
No MS^2 Spectrum,
RT verified with other samples

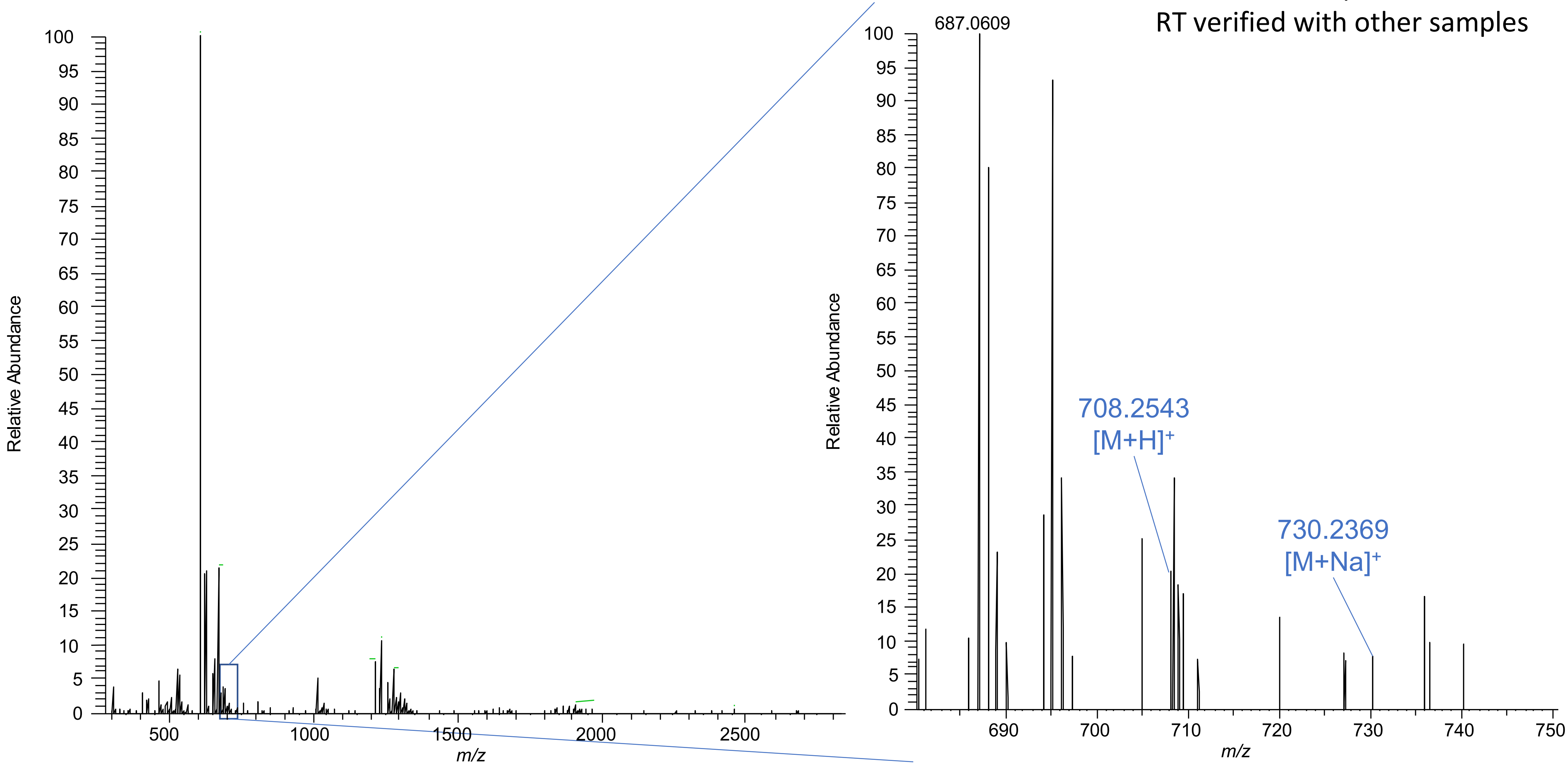

#18c Lacto-N(-neo)-tetraose (LN(n)T)

MS^2 Spectrum RT 7.67 min
730.2377 *m/z*

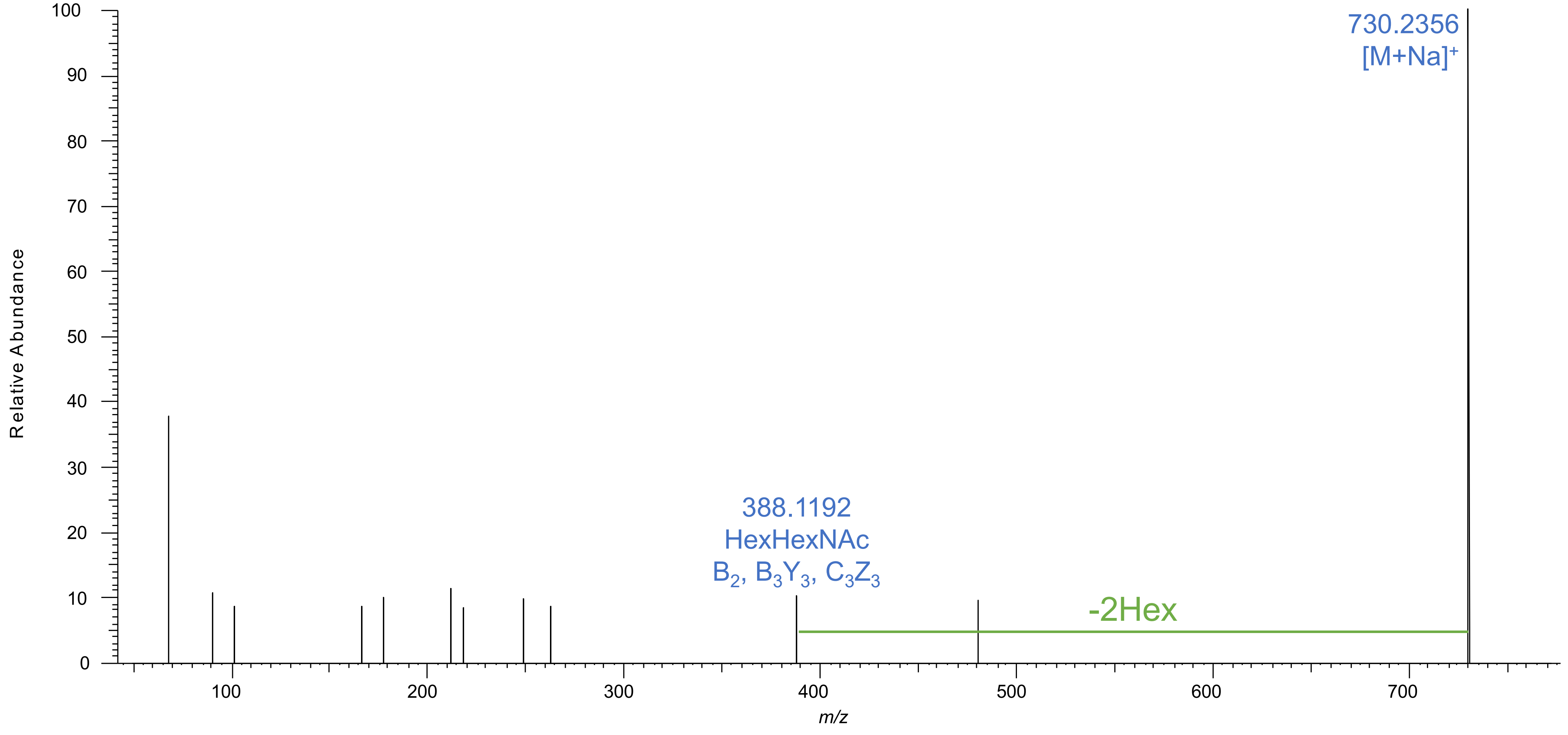

#19 3_3_0_0_0

MS[2] Spectrum RT 11.83 min
1114.4133 *m/z*

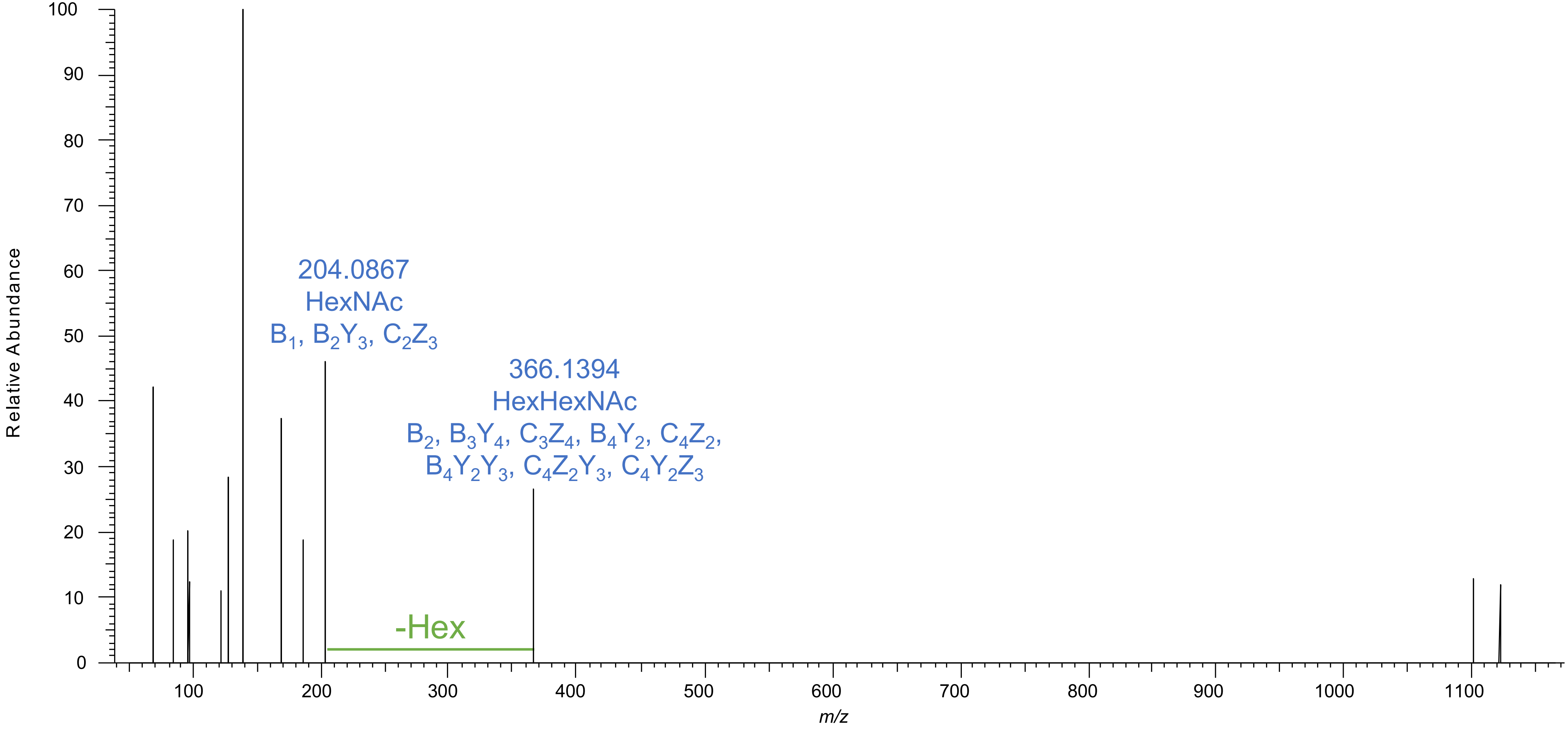

#20 4_1_0_0_0

MS Spectrum RT: 10.96 min
No MS^2 Spectrum,
RT verified with other samples

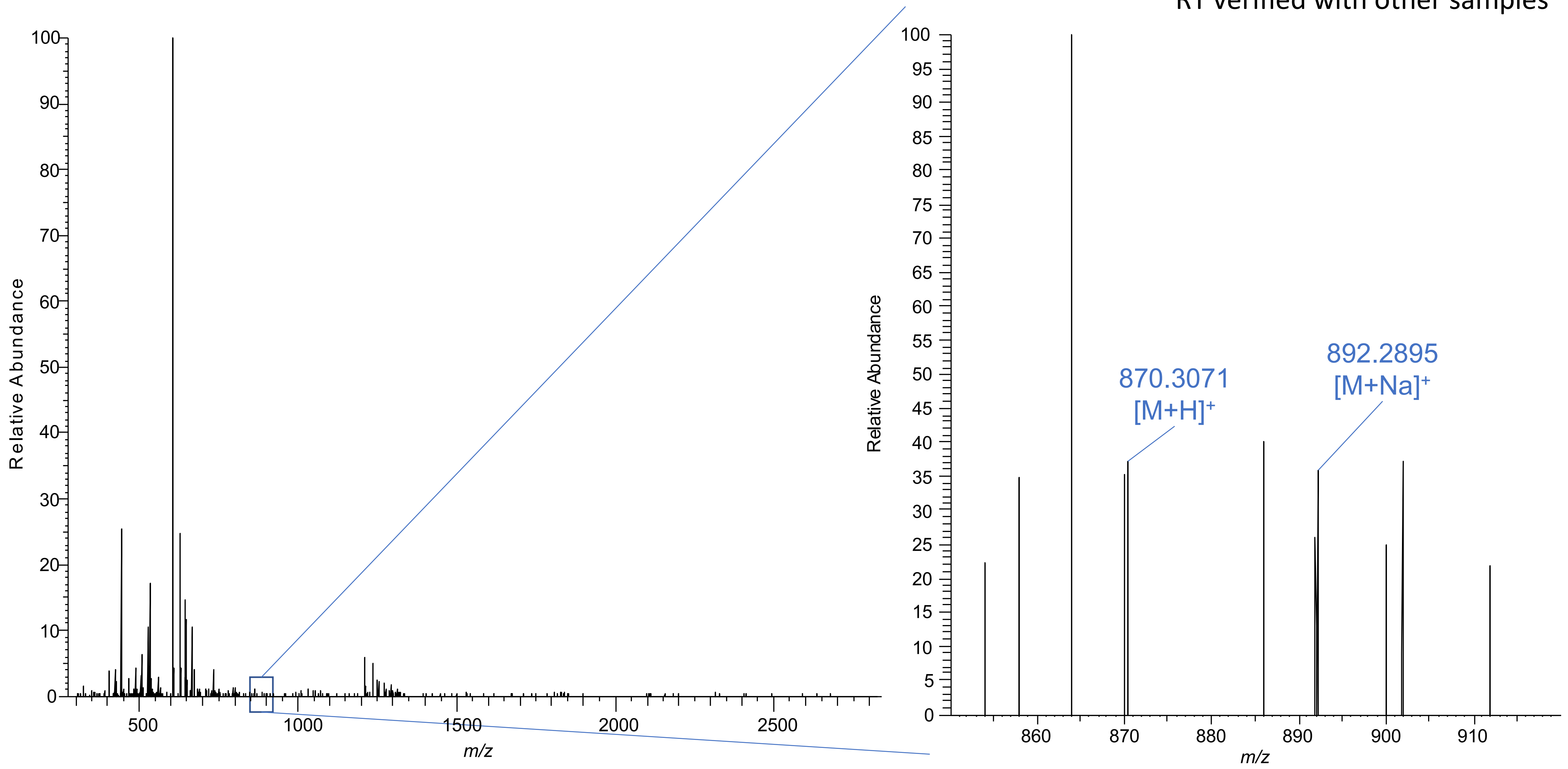

#21 Lacto-N(-neo)-hexaose (LN(n)H)

MS^2 Spectrum RT 12.87 min
1095.3698 *m/z*

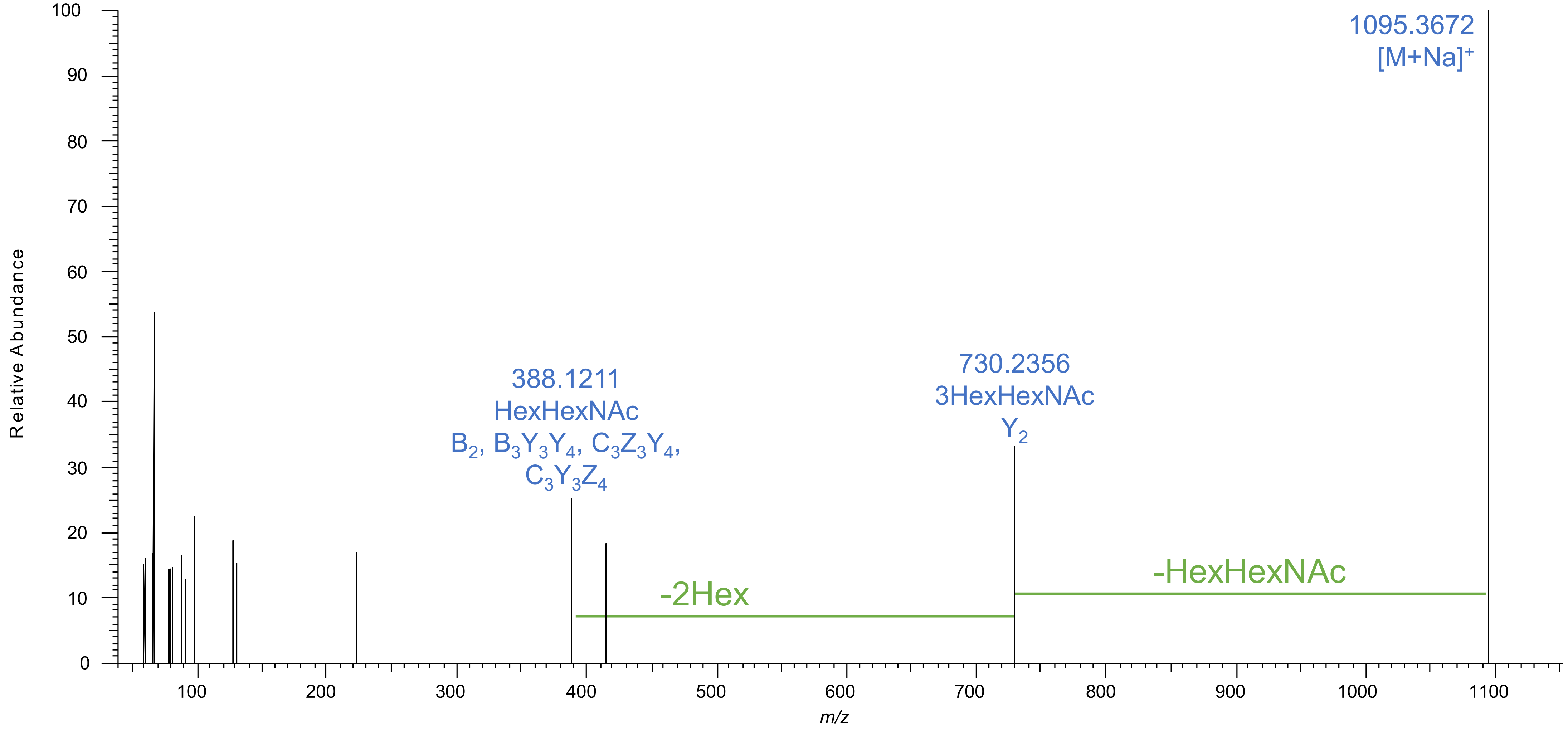

#22 Neu5Gc-LN(n)H (NG-LN(n)H)

MS Spectrum RT: 17.95 min
No MS^2 Spectrum,
RT verified with other samples

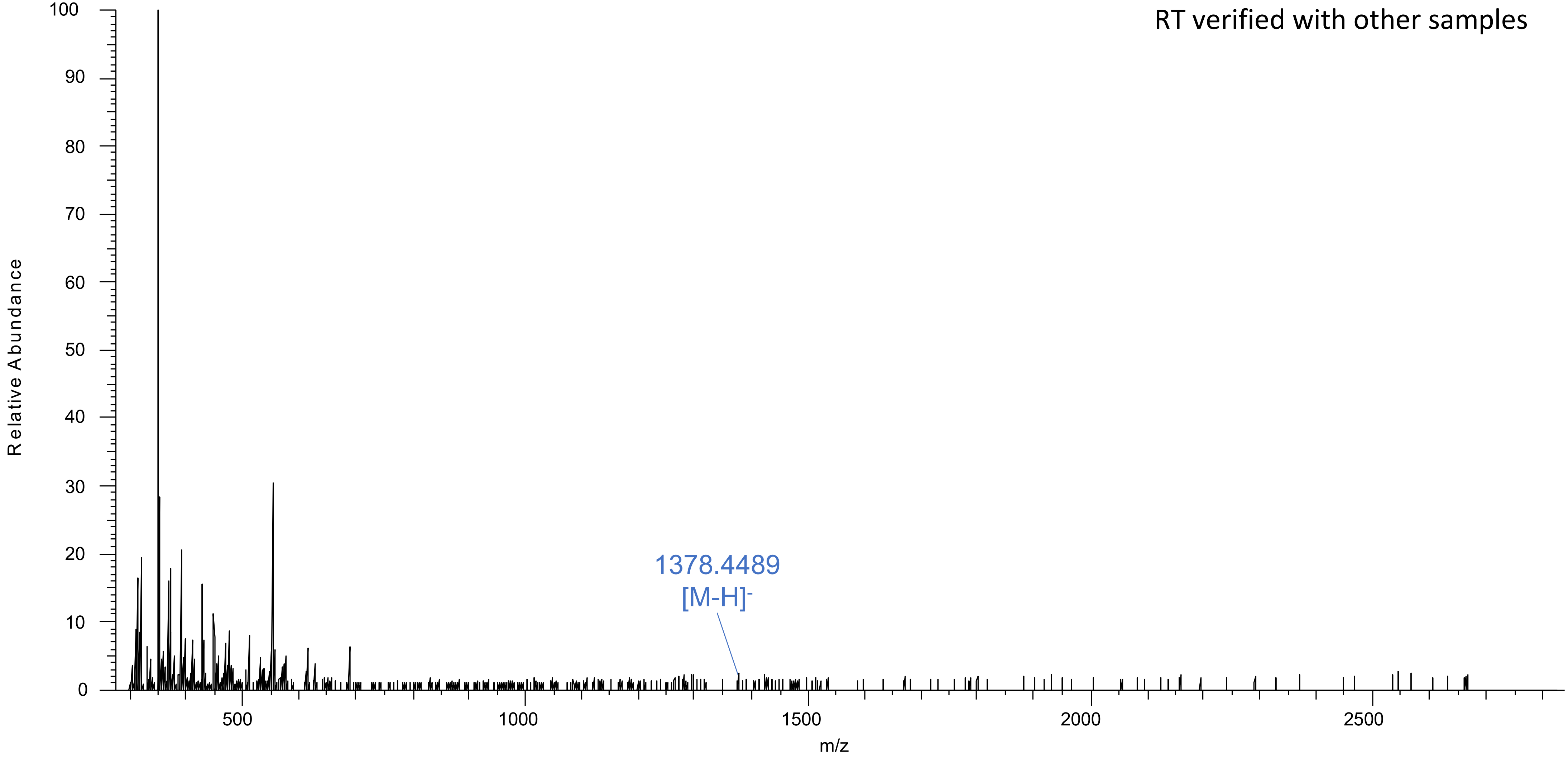

#23 Sialyl-LN(n)H (S-LN(n)H)

MS Spectrum RT: 16.47 min
No MS^2 Spectrum,
RT verified with other samples

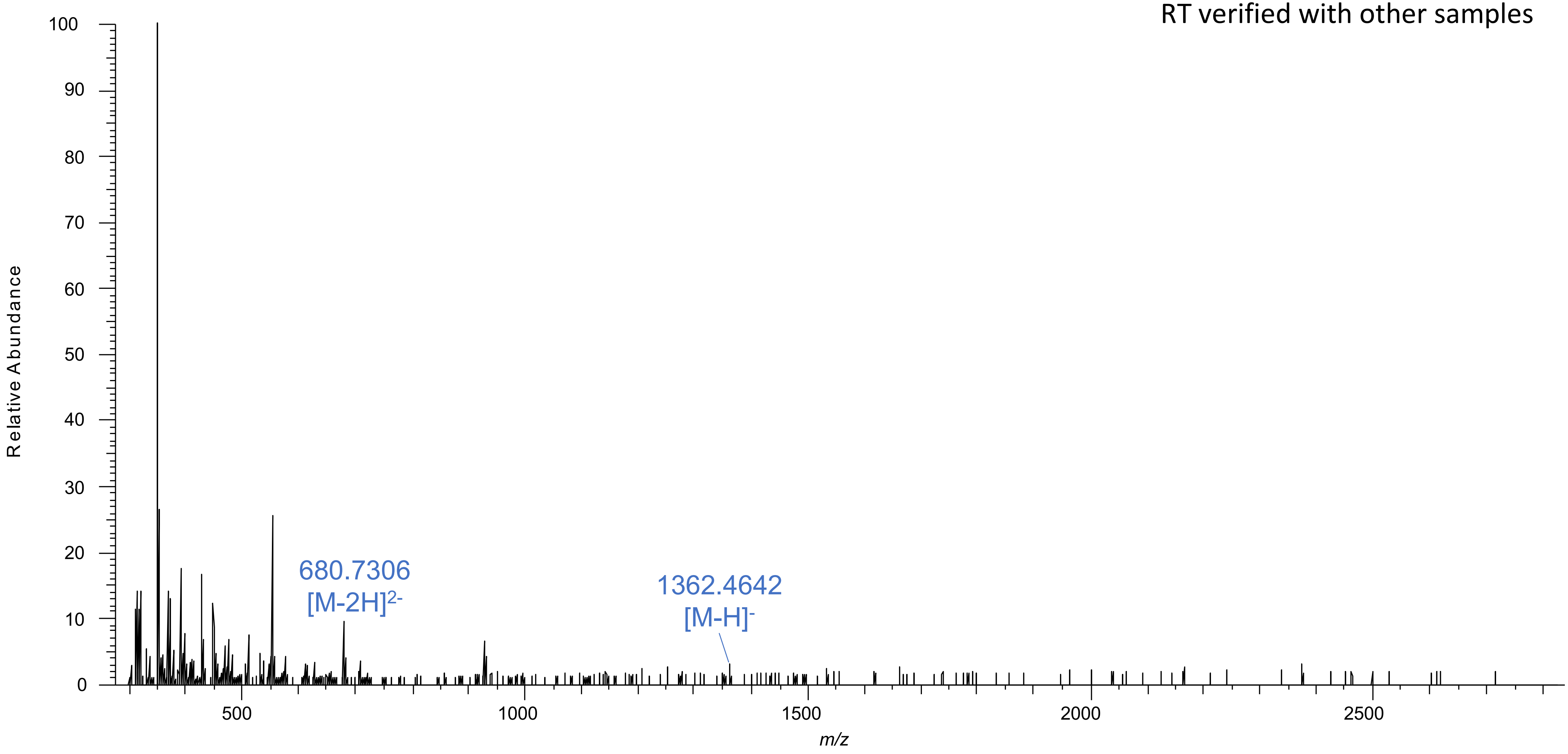

East Friesian Sheep

Figure description

- MS spectra of MOs found in East Friesian sheep at the corresponding retention time extracted from the ThermoFisher Scientific Software Freestyle.
- The MO categories are indicated in the upper-left corner by a symbol obtained from BioRender.com and the fragments were computed using GlycoWorkbench 2 (Ceroni et al., 2008).
- Fragments, which were linkage or order-specific, were highlighted in red.
- yellow circle, neutral-nonfucosylated; red triangle, neutral-fucosylated; purple diamond, Neu5Ac-sialylated; light blue diamond, Neu5Gc-sialylated

#1a 3‘-Neu5Gc-lactosamine (3‘-NGLN)

MS[2] Spectrum RT 6.05 min
689.2260 *m/z*

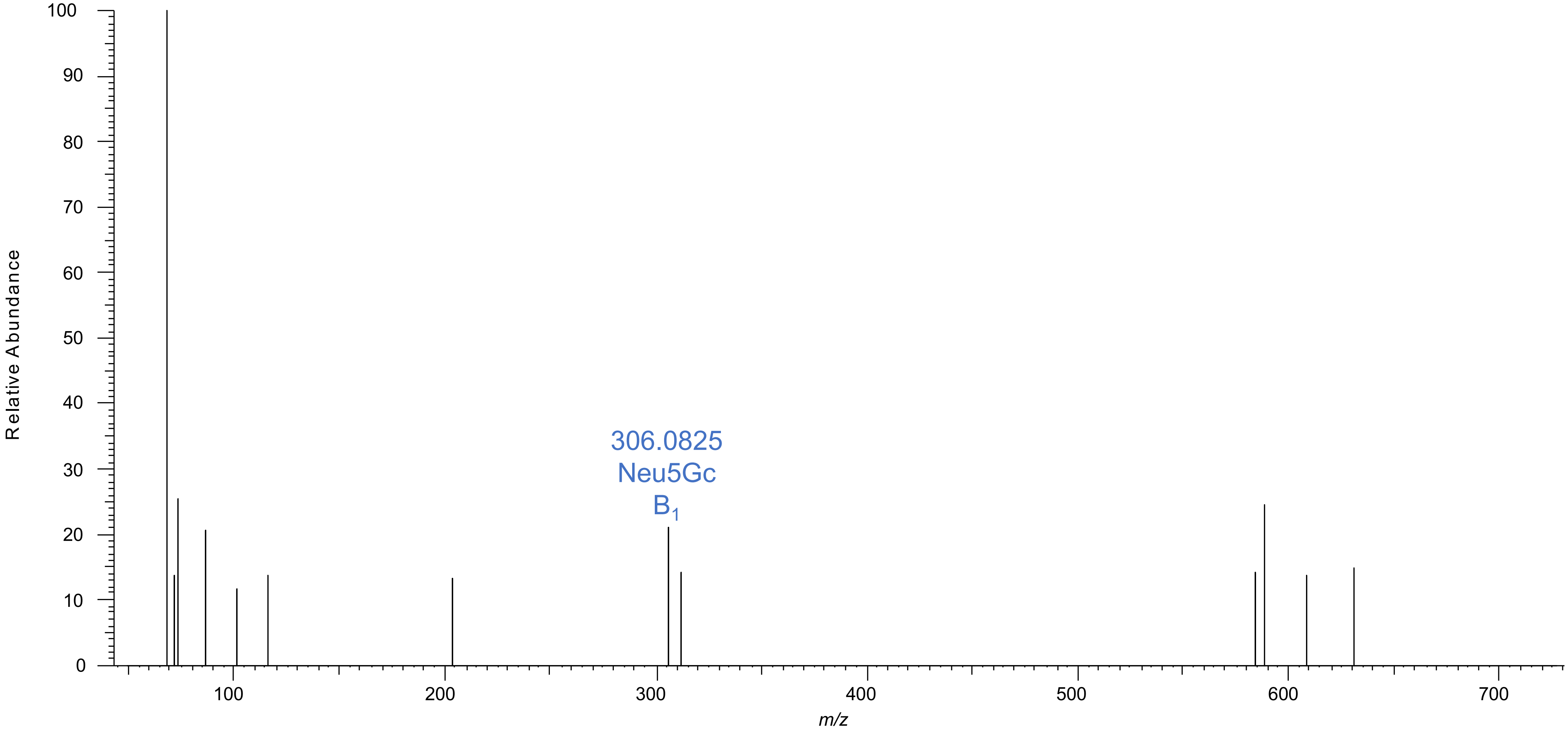

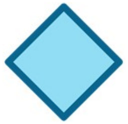

#1b 6‘-Neu5Gc-lactosamine (6‘-NGLN)

MS[2] Spectrum RT 6.56 min
No MS[2] Spectrum,
RT verified with other samples

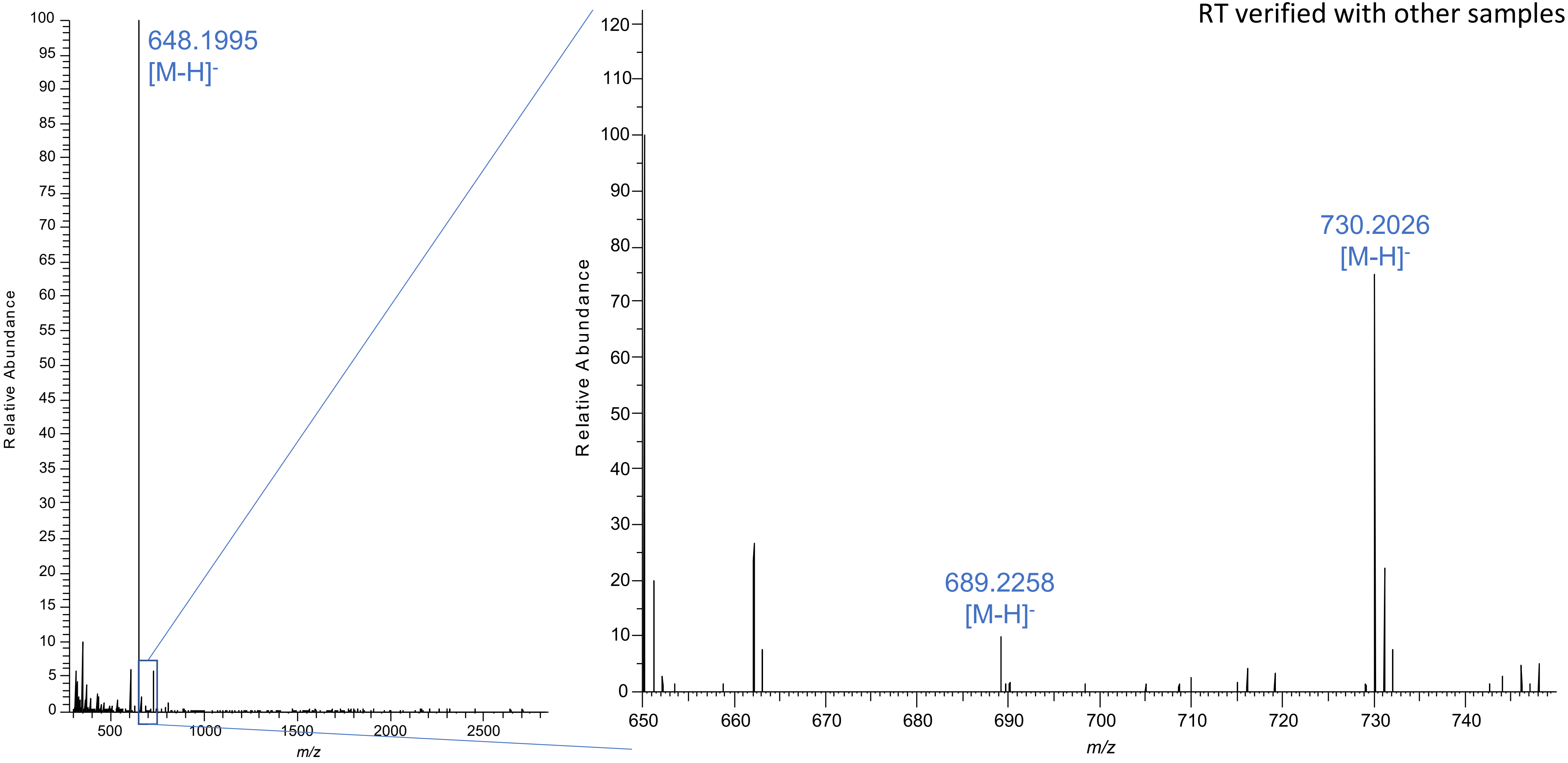

#3a 3‘-Neu5Gc-lactose (3‘-NGL)

MS^2 Spectrum RT 6.71 min
648.1992 *m/z*

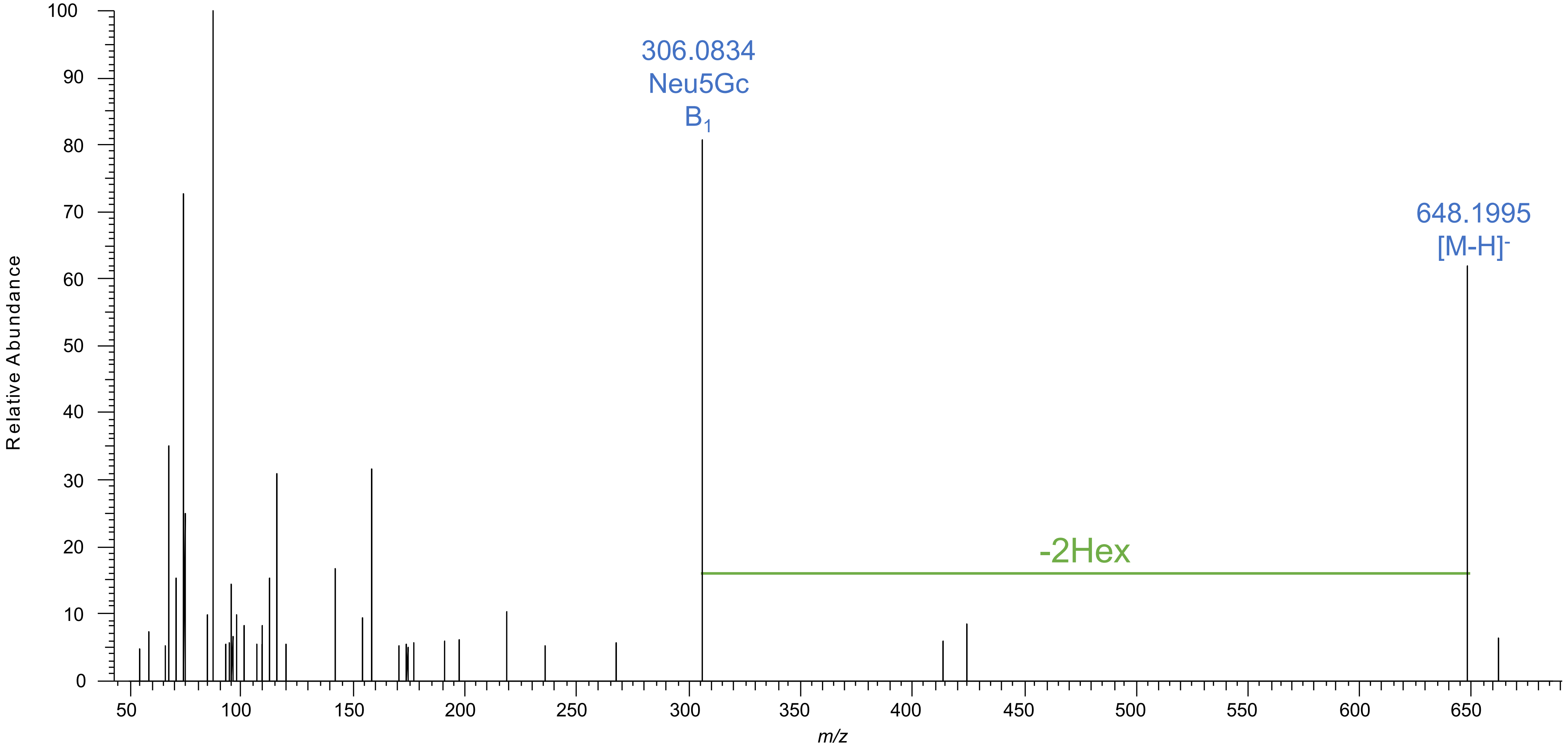

#3b 6‘-Neu5Gc-lactose (6‘-NGL)

MS^2 Spectrum RT 7.86 min
648.1999 *m/z*

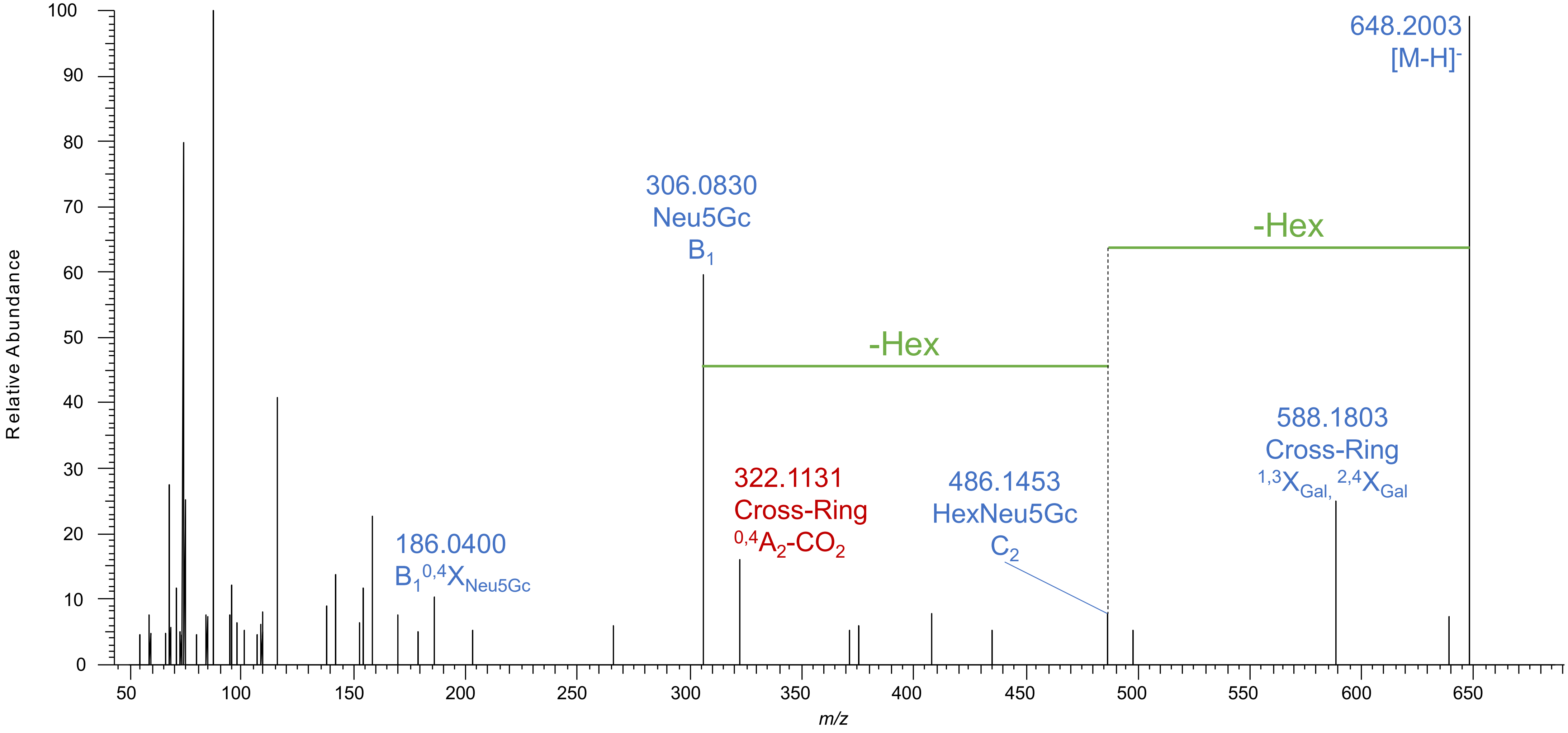

#4 Di-Neu5Gc-lactose (DNGL)

MS Spectrum RT: 12.45 min
No MS^2 Spectrum, see goat

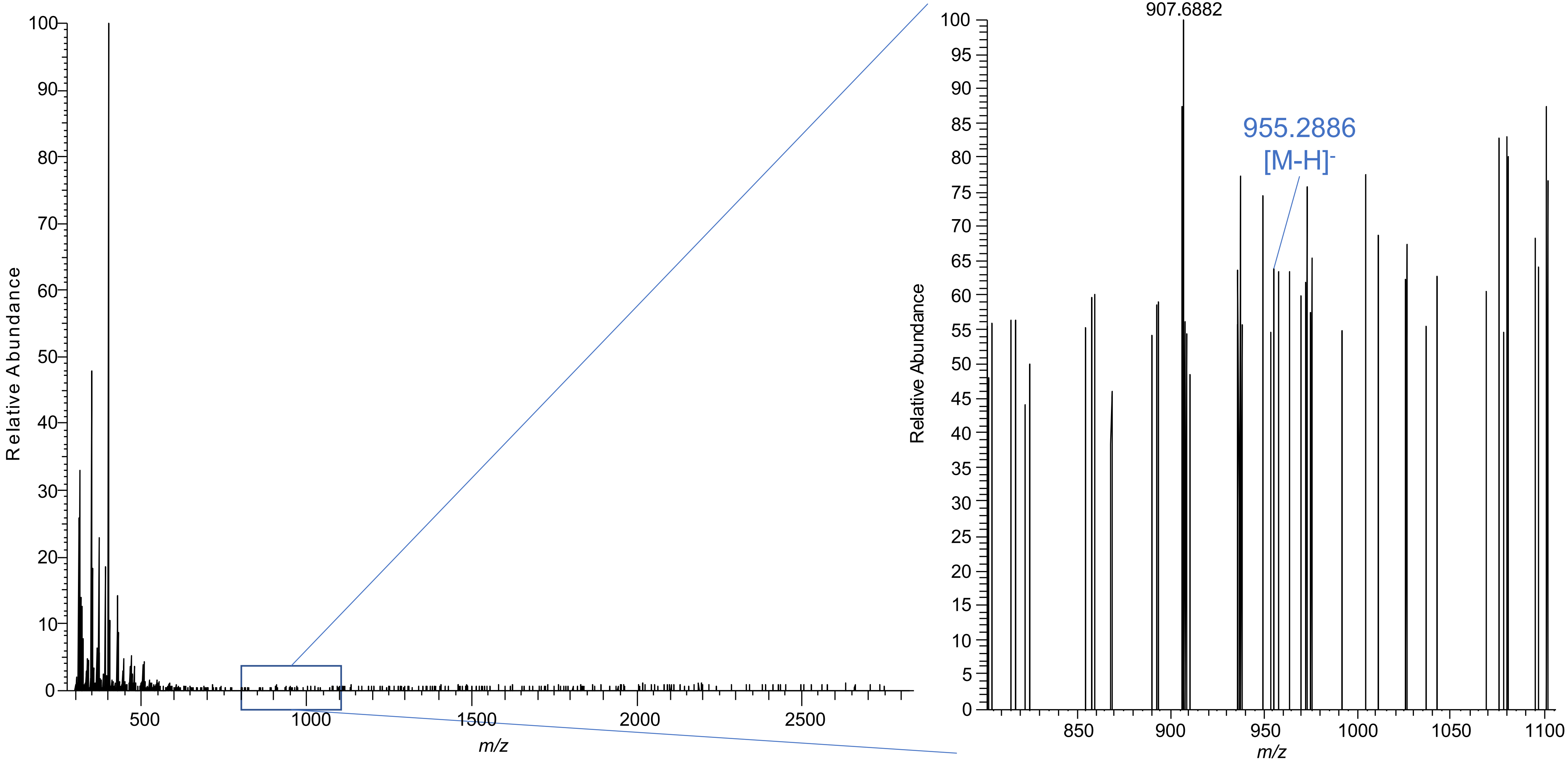

#4L Di-Neu5Gc-lactose (DNGL) lactonized

MS Spectrum RT: 9.43 min
No MS^2 Spectrum,
RT verified with other samples

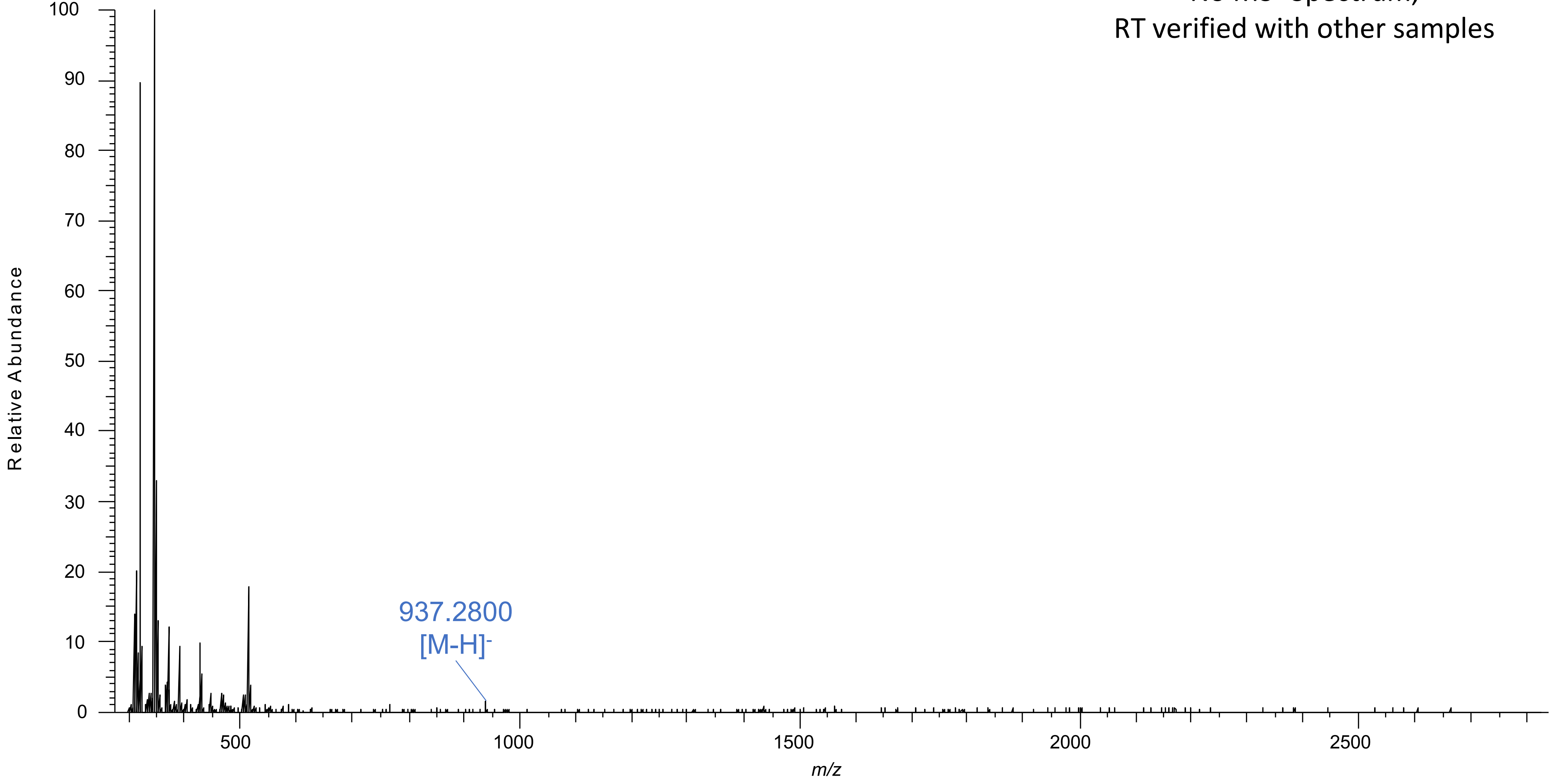

#5a 3'-Sialyllactose (3'-SL)

MS^2 Spectrum RT 5.29 min
632.2048 *m/z*

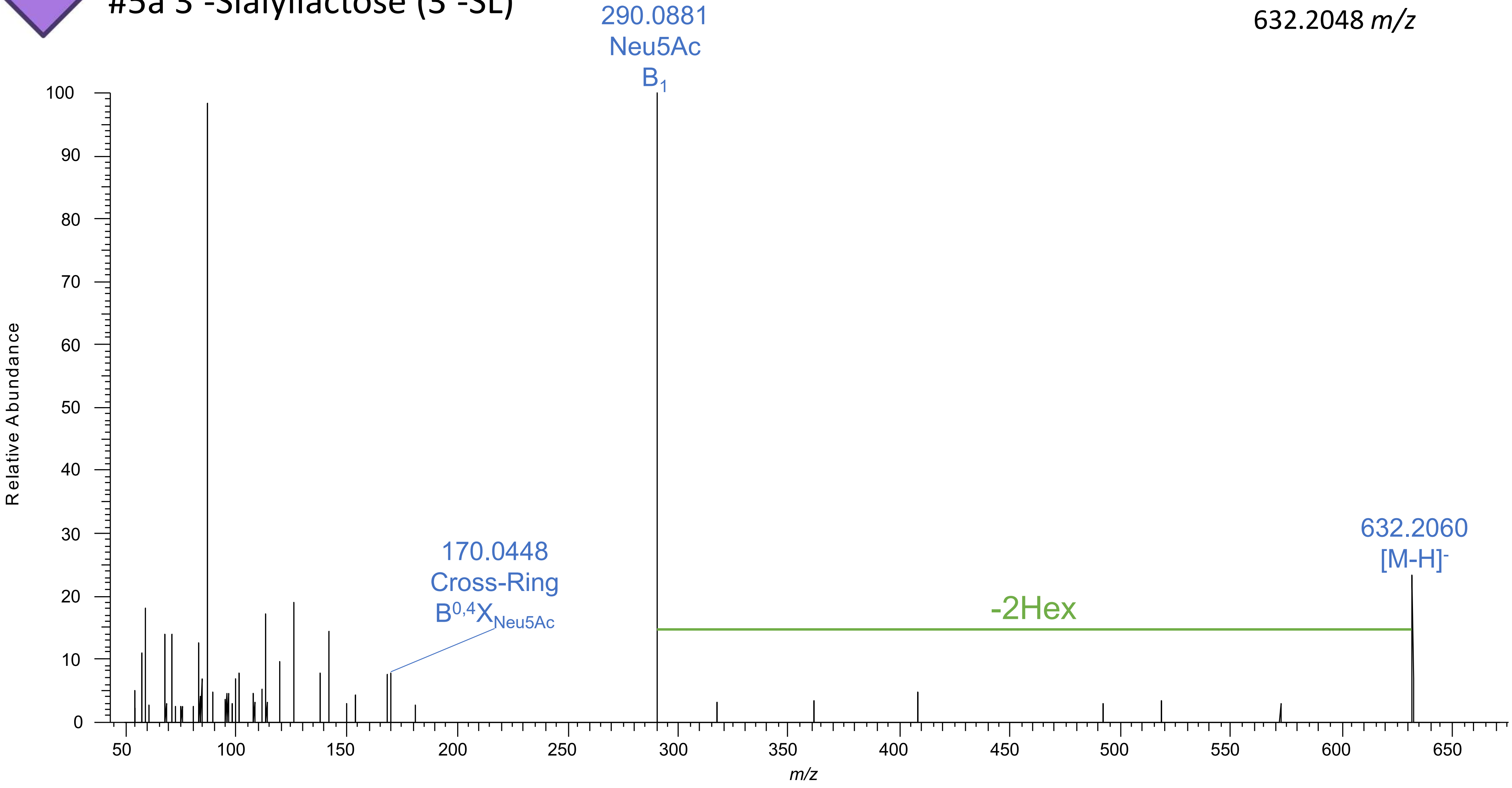

#5b 6‘-Sialyllactose (6‘-SL)

MS^2 Spectrum RT 6.38 min
632.2039 *m/z*

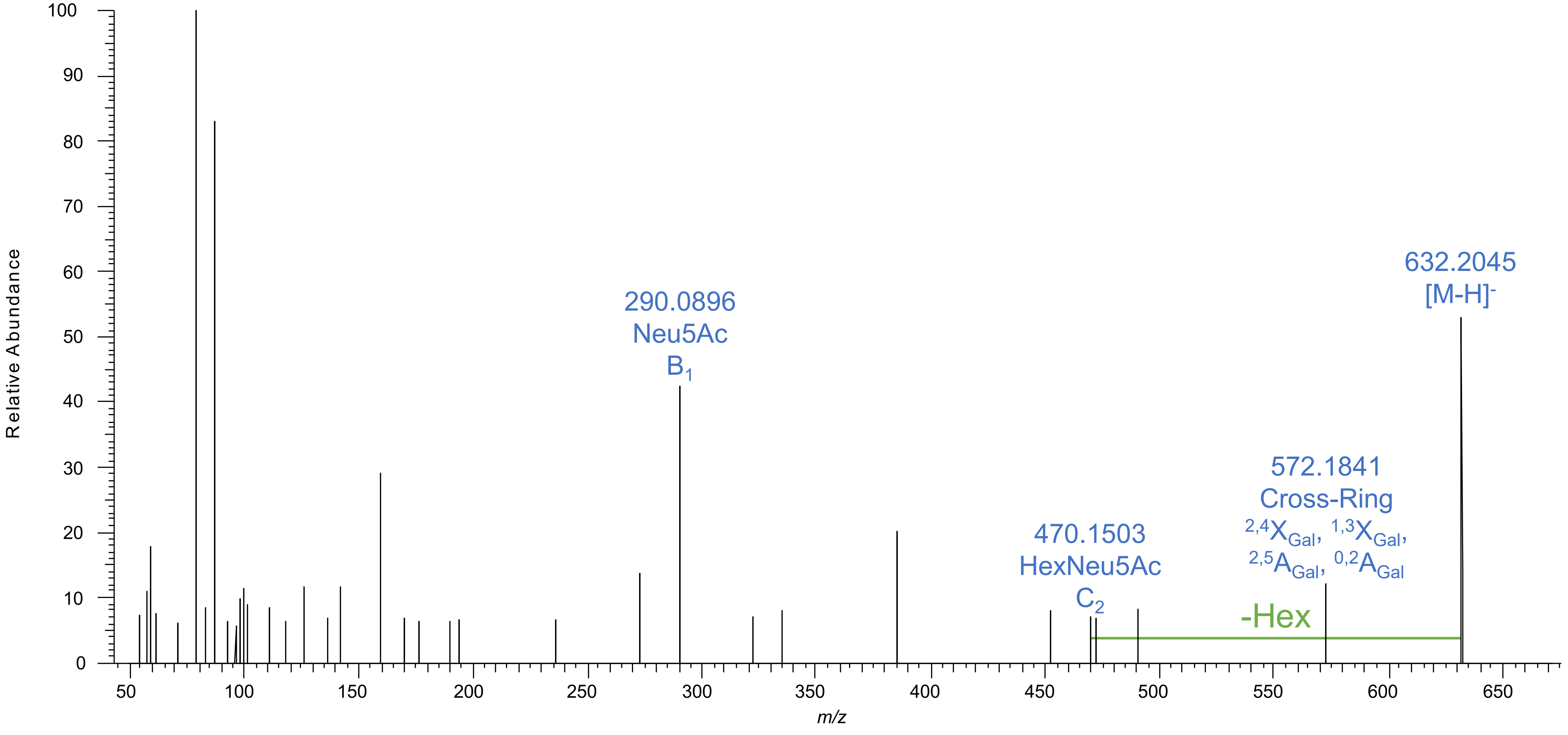

#8 Fucosyllactose (FL)

MS Spectrum RT: 4.05 min
No MS^2 Spectrum, see goat

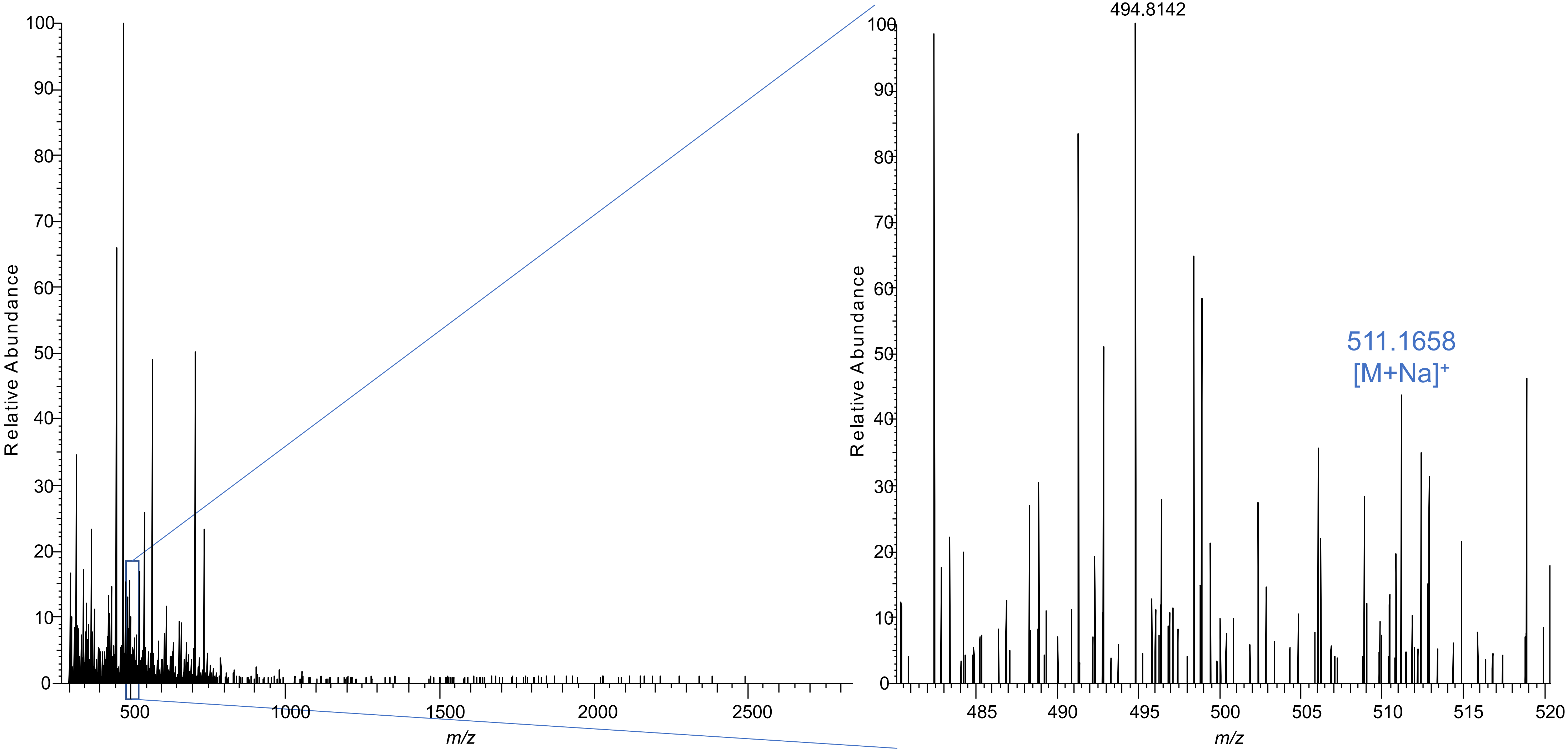

#11 2_1_1_0_0

MS Spectrum RT: 5.80 min
No MS^2 Spectrum,
RT verified with other samples

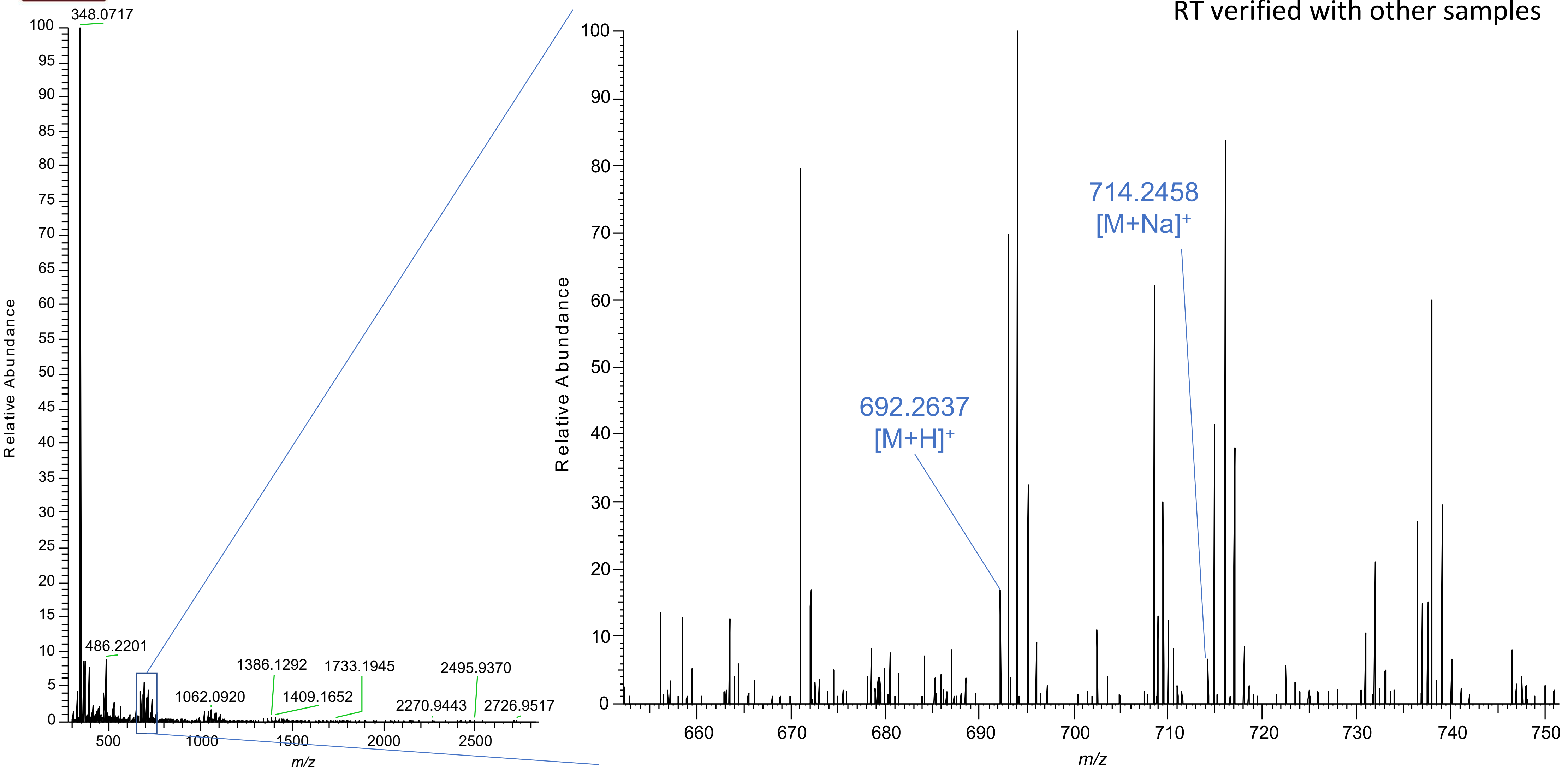

#12b Galactosyllactose (GL)

MS^2 Spectrum RT 6.00 min
527.1603 *m/z*

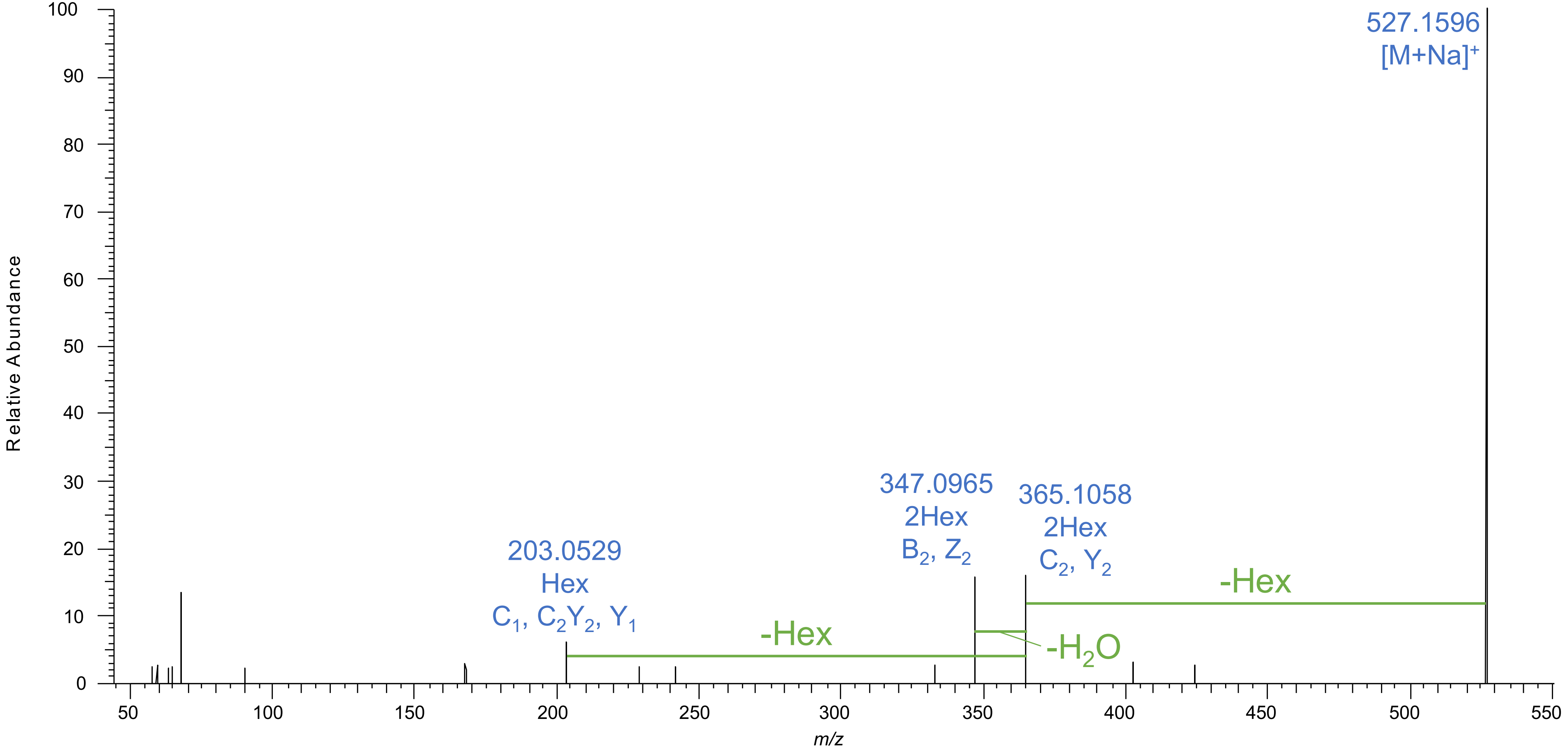

#13a Neu5Gc-GL (NG-GL)

MS^2 Spectrum RT 9.88 min
810.2527 *m/z*

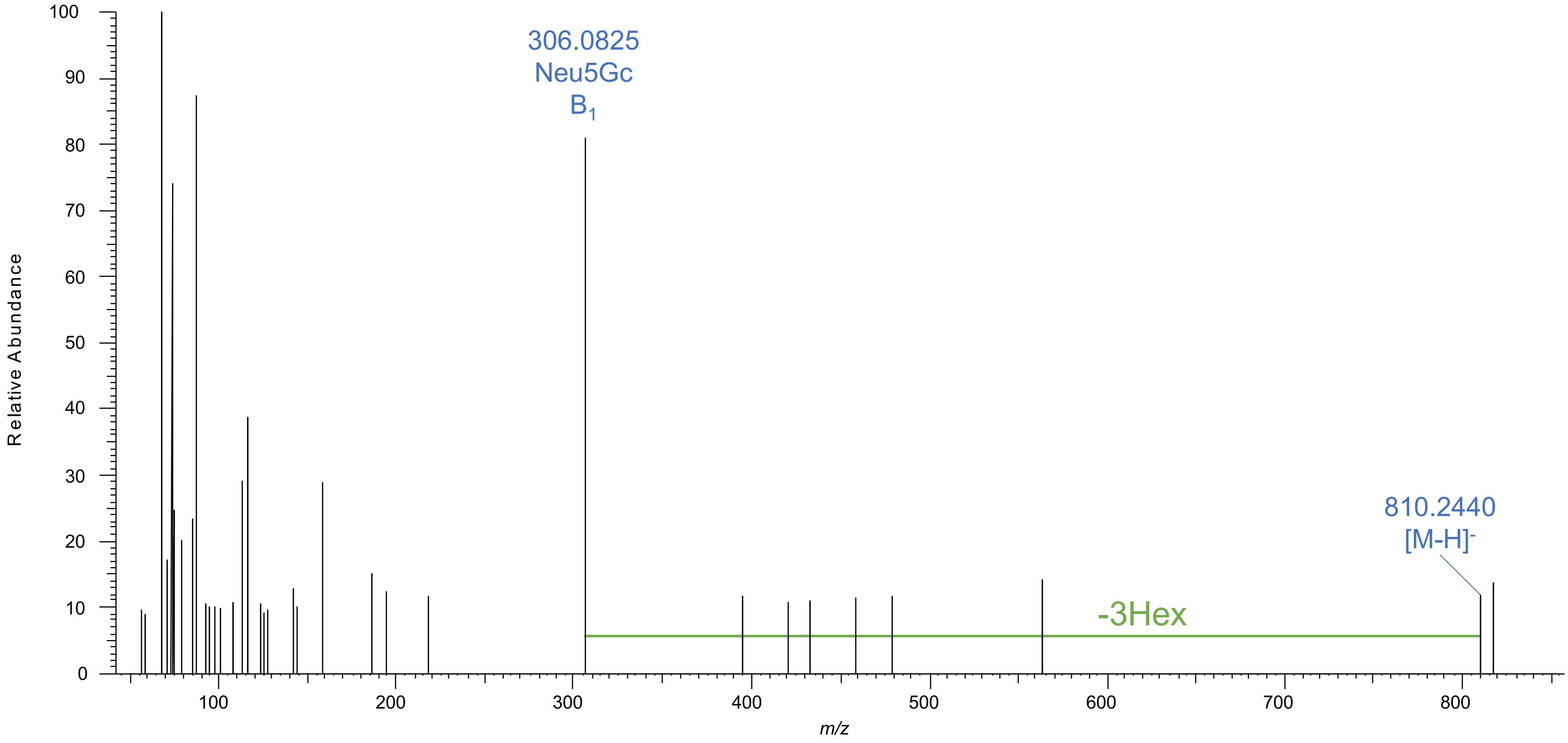

#13b Neu5Gc-GL (NG-GL)

MS Spectrum RT: 10.52 min
No MS^2 Spectrum, see goat

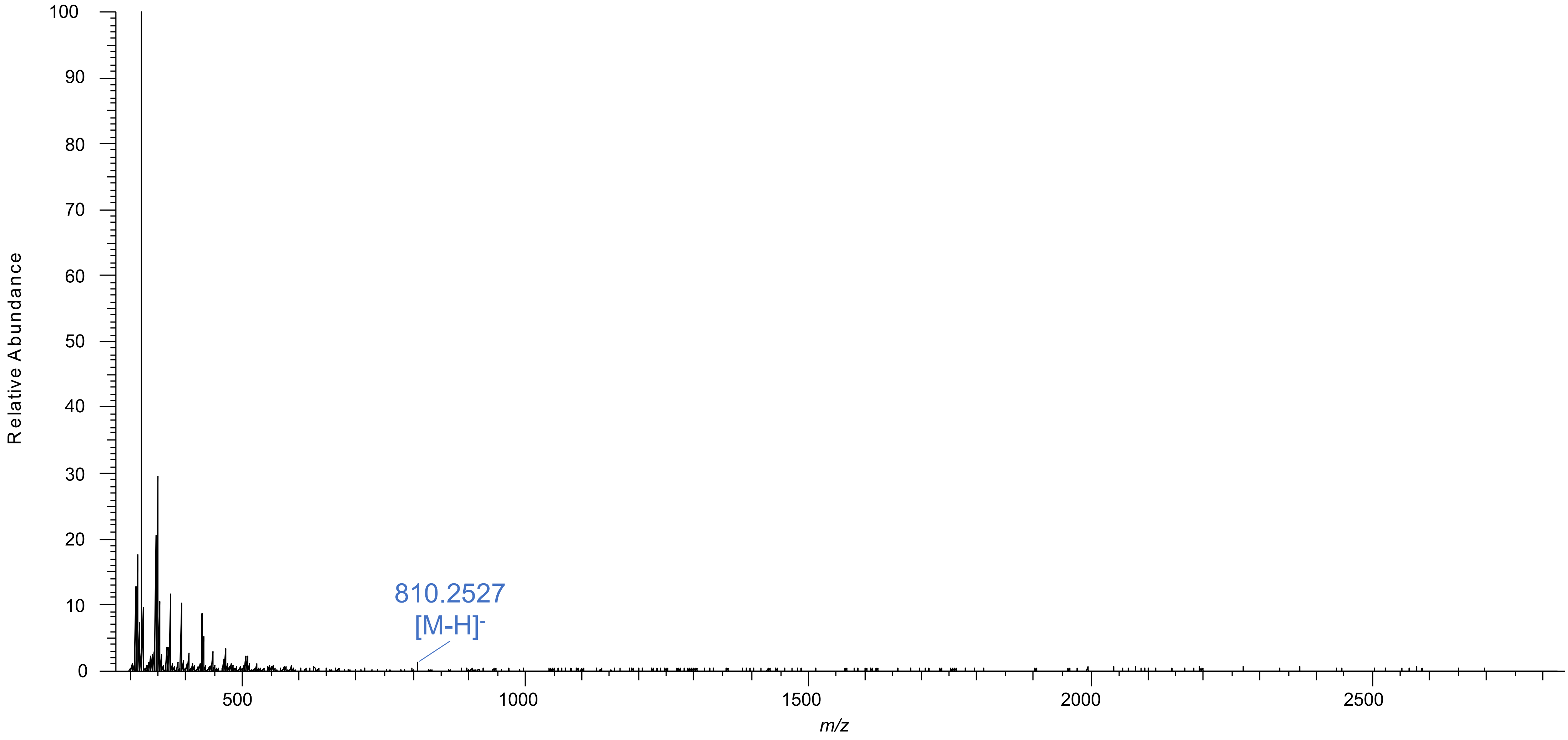

#14 Neu5Gc-Disialyl-triose

MS Spectrum RT: 15.51 min
No MS^2 Spectrum, see goat

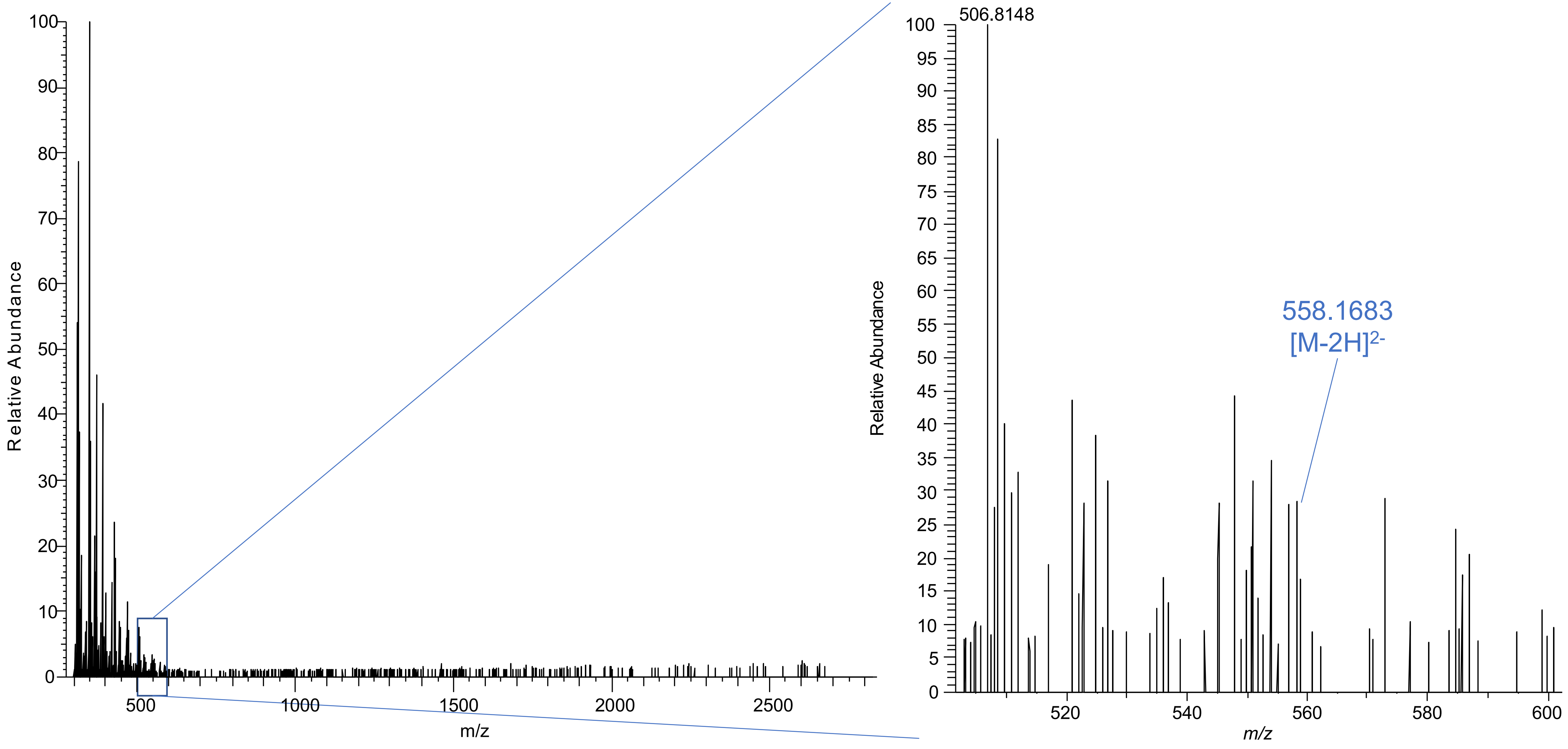

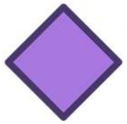

#15a Sialyl-GL (SGL)

MS Spectrum RT: 8.20 min
No MS^2 Spectrum, see goat

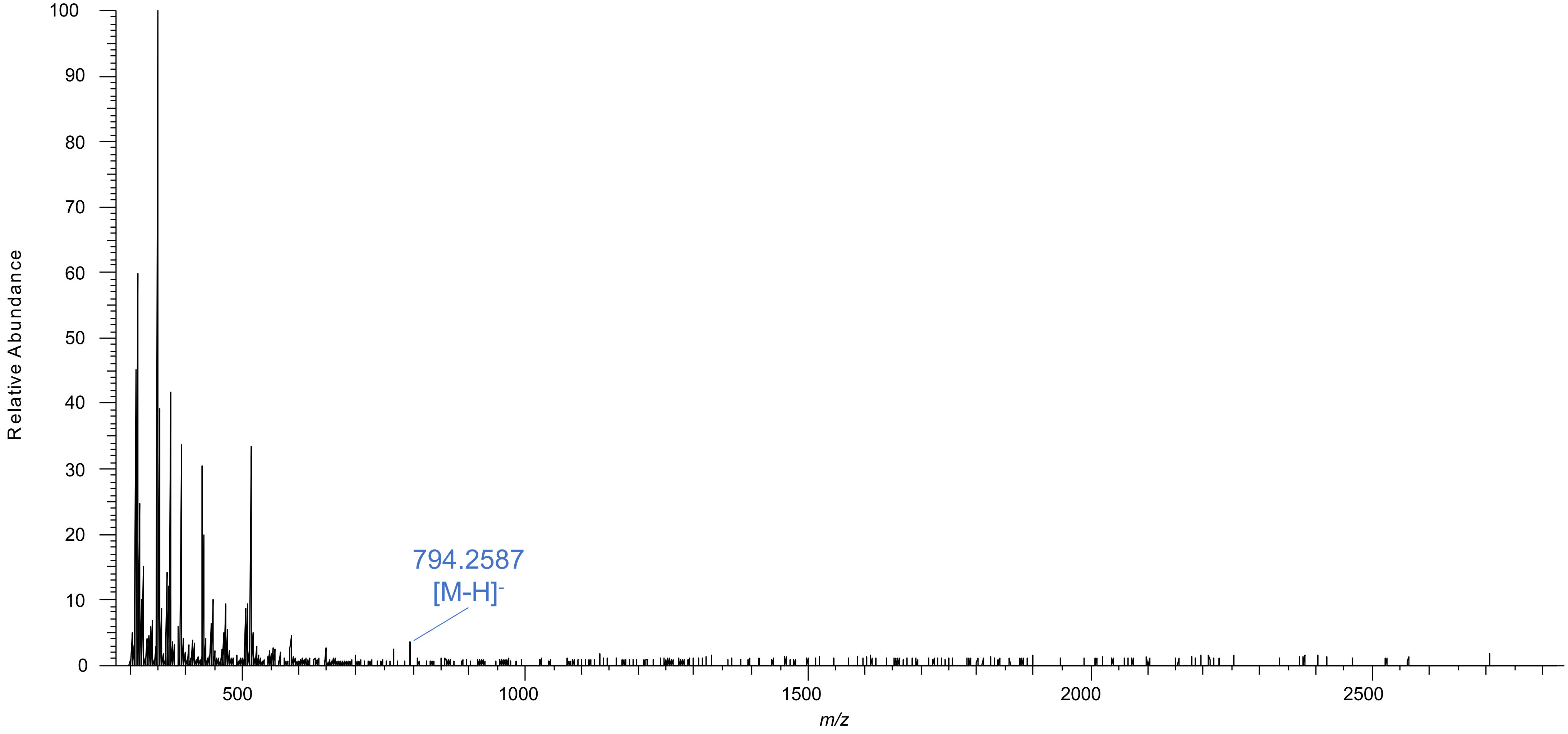

#18a Lacto-N(-neo)-tetraose (LN(n)T)

MS Spectrum RT: 7.25 min
No MS^2 Spectrum,
RT verified with other samples

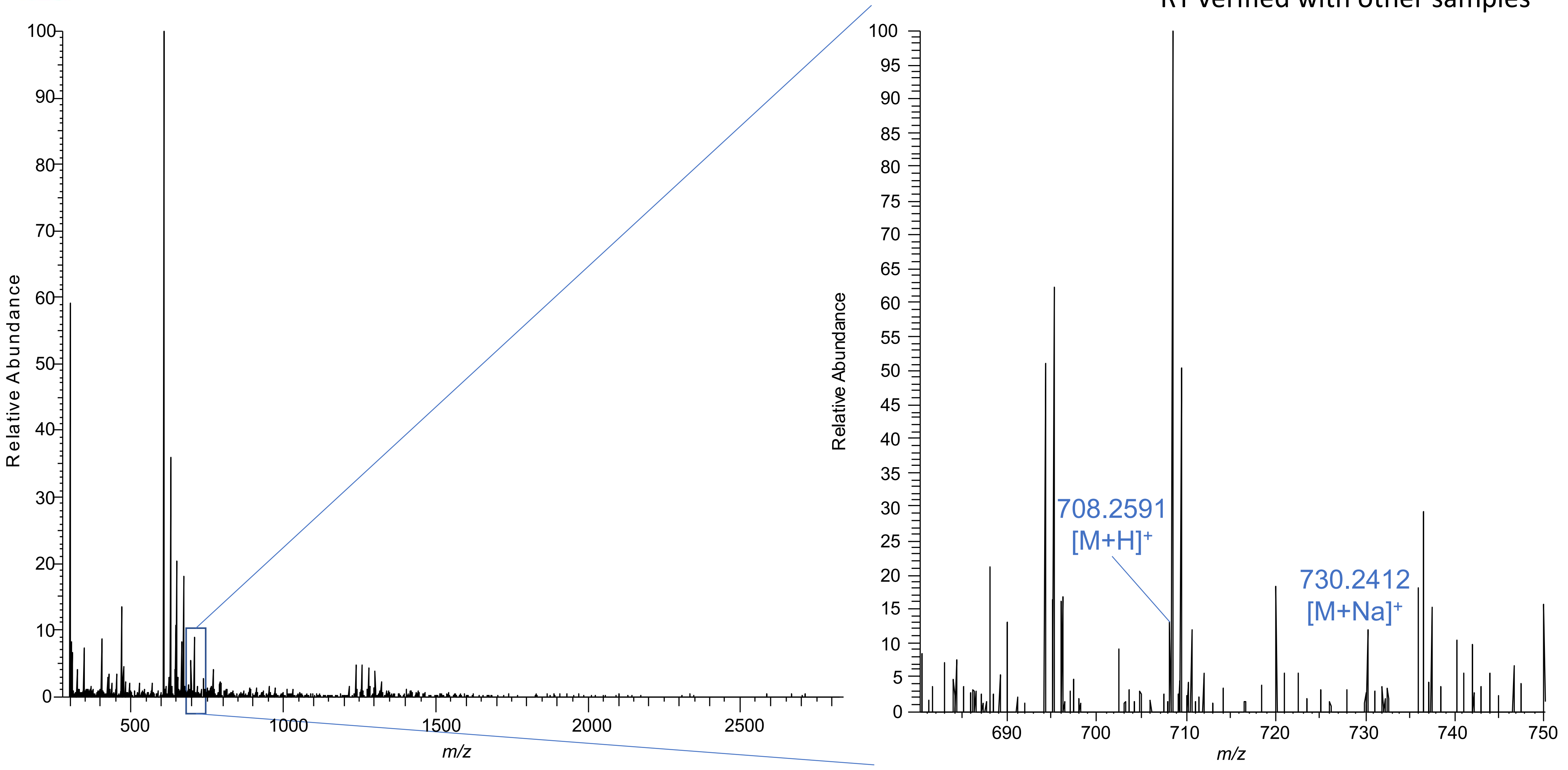

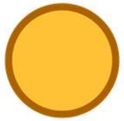

#19 3_3_0_0_0

MS Spectrum RT: 11.83 min

No MS^2 Spectrum, see goat

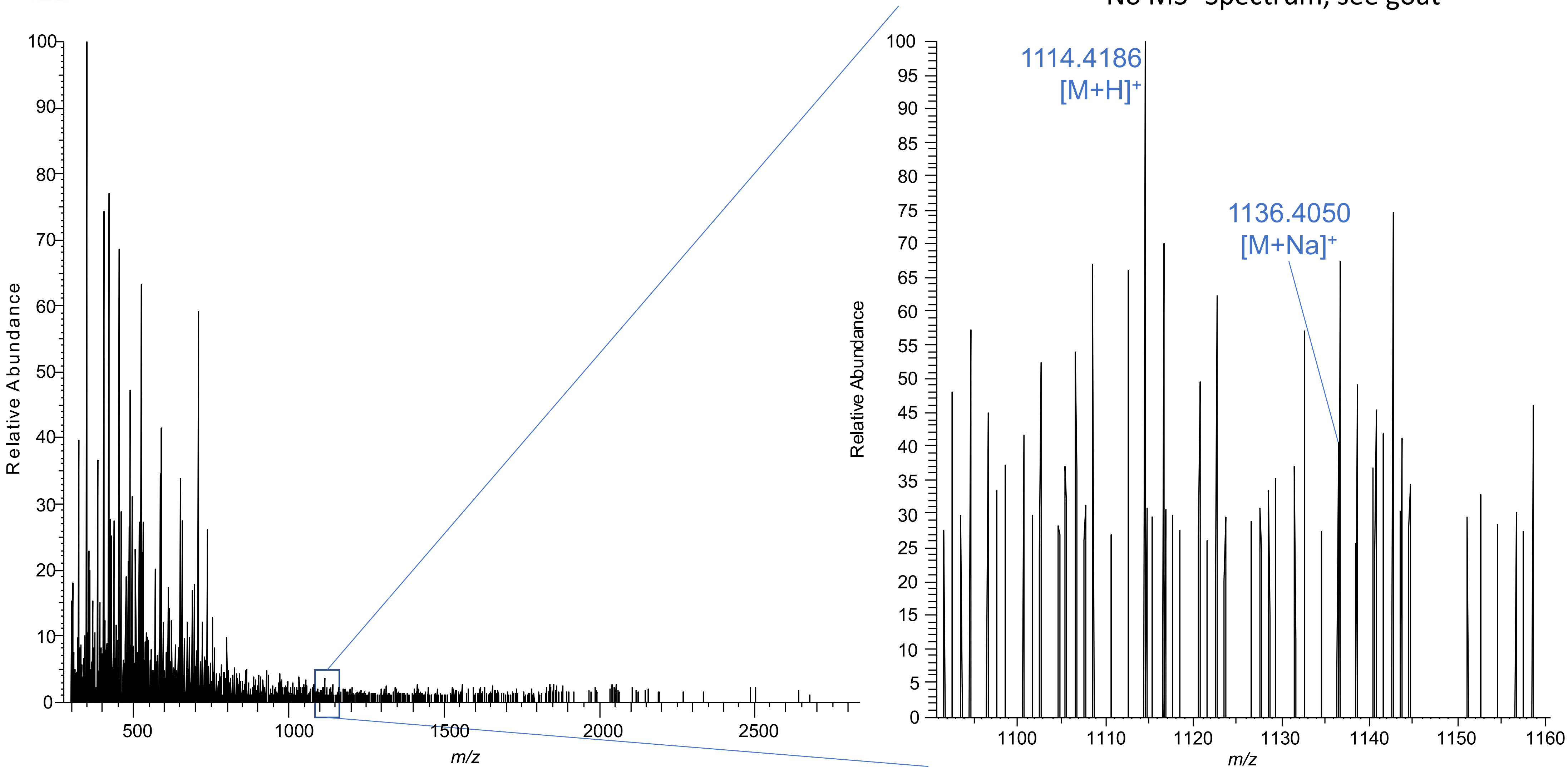

#21 Lacto-N(-neo)-hexaose (LN(n)H)

MS Spectrum RT: 12.85 min
No MS^2 Spectrum, see goat

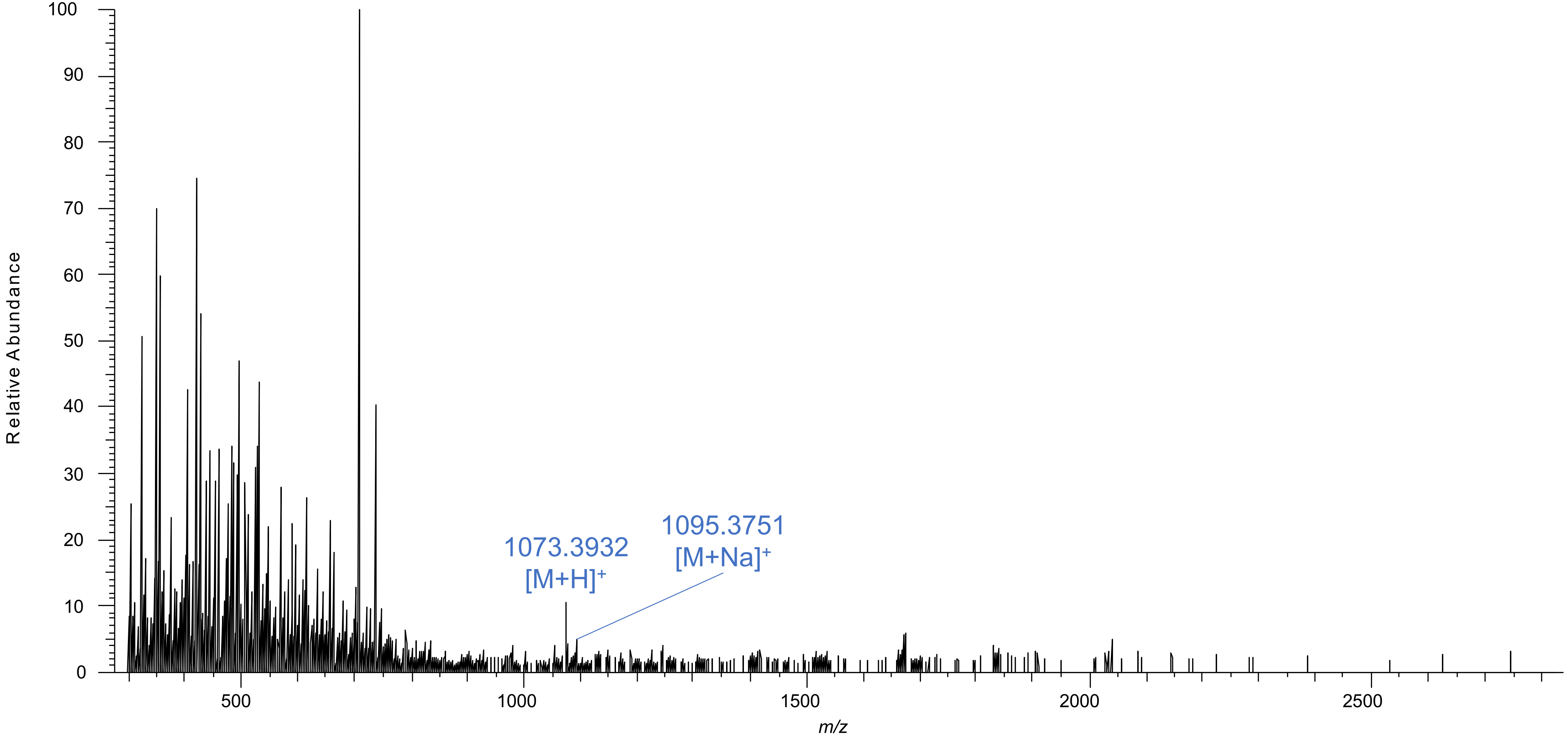

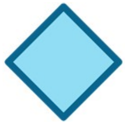

#22 Neu5Gc-LN(n)H (NG-LN(n)H)

MS Spectrum RT: 17.74 min
No MS^2 Spectrum,
RT verified with other samples

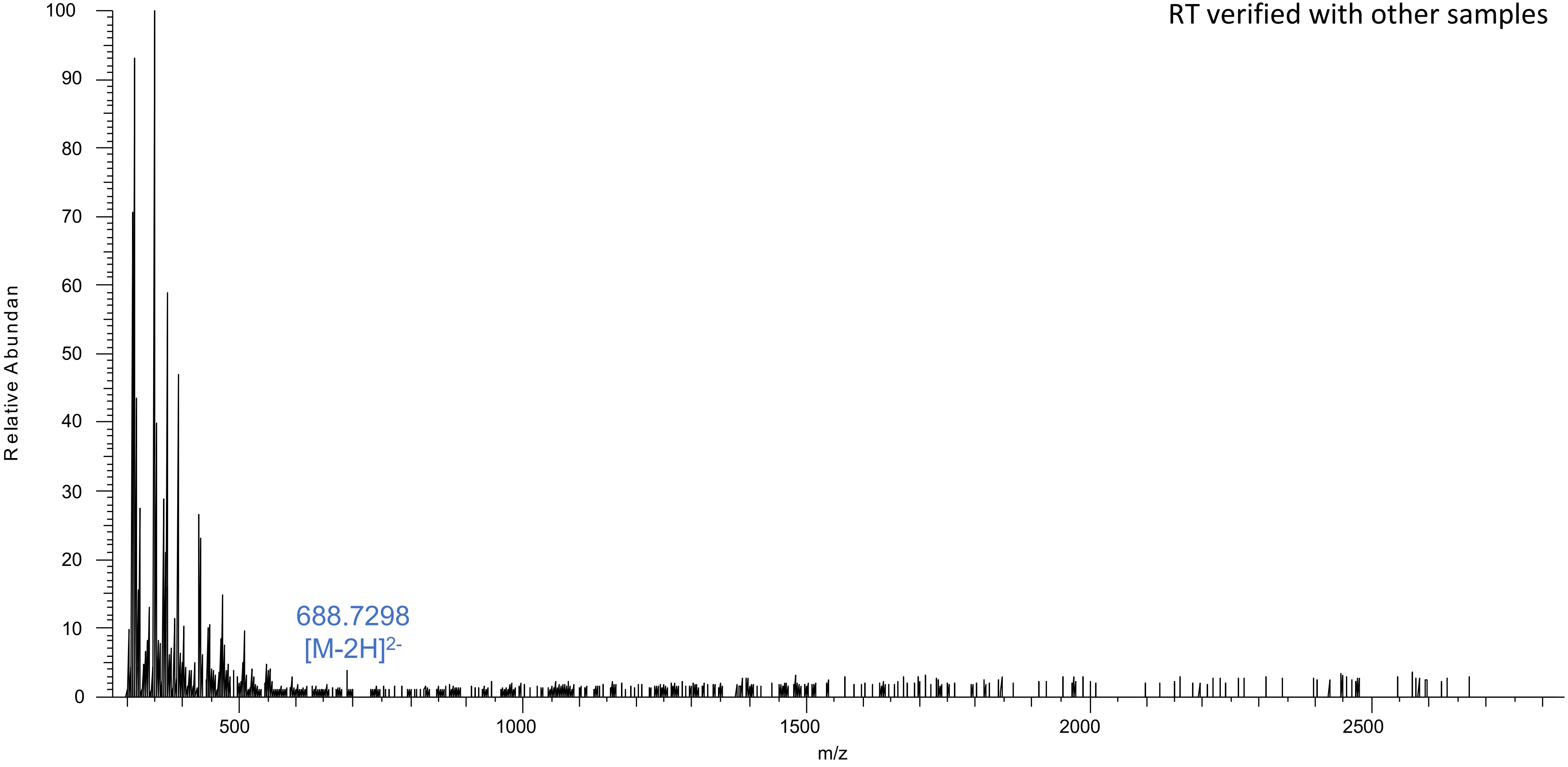

Supplement: Supplementary file 2 [file ao5c13396_si_002.pdf]
