# Supplementary material for: Analysis of the Milk Oligosaccharides Spectrum and Sialylation Status of Milk from West African Dwarf Goat and East Friesian Sheep
Source: ACS Omega. 2026 May 27;11(22):32310–20. doi: 10.1021/acsomega.5c13396 (PMC13261409; doi:10.1021/acsomega.5c13396)
Supplement: Supplementary file 3 [file ao5c13396_si_003.pdf]

Figure S3

Analysis of the Milk Oligosaccharides Spectrum and Sialylation Status of Milk from West African Dwarf Goat and East Friesian Sheep

Lisa Isernhagen[a], Christina E. Galuska[a], Andreas Hoeflich[a] and Sebastian P. Galuska[a, *]

[a]: Research Institute for Farm Animal Biology (FBN), Wilhelm-Stahl-Allee 2, 18196 Dummerstorf, Germany

[*]: Corresponding author: Sebastian P. Galuska, Galuska.sebastian@fbn-dummerstorf.de

Figure description

- Extended version of the literature comparison visualization for goat and sheep MOs (Figure 3 and 4) including also the references with less than 5 identified MOs.

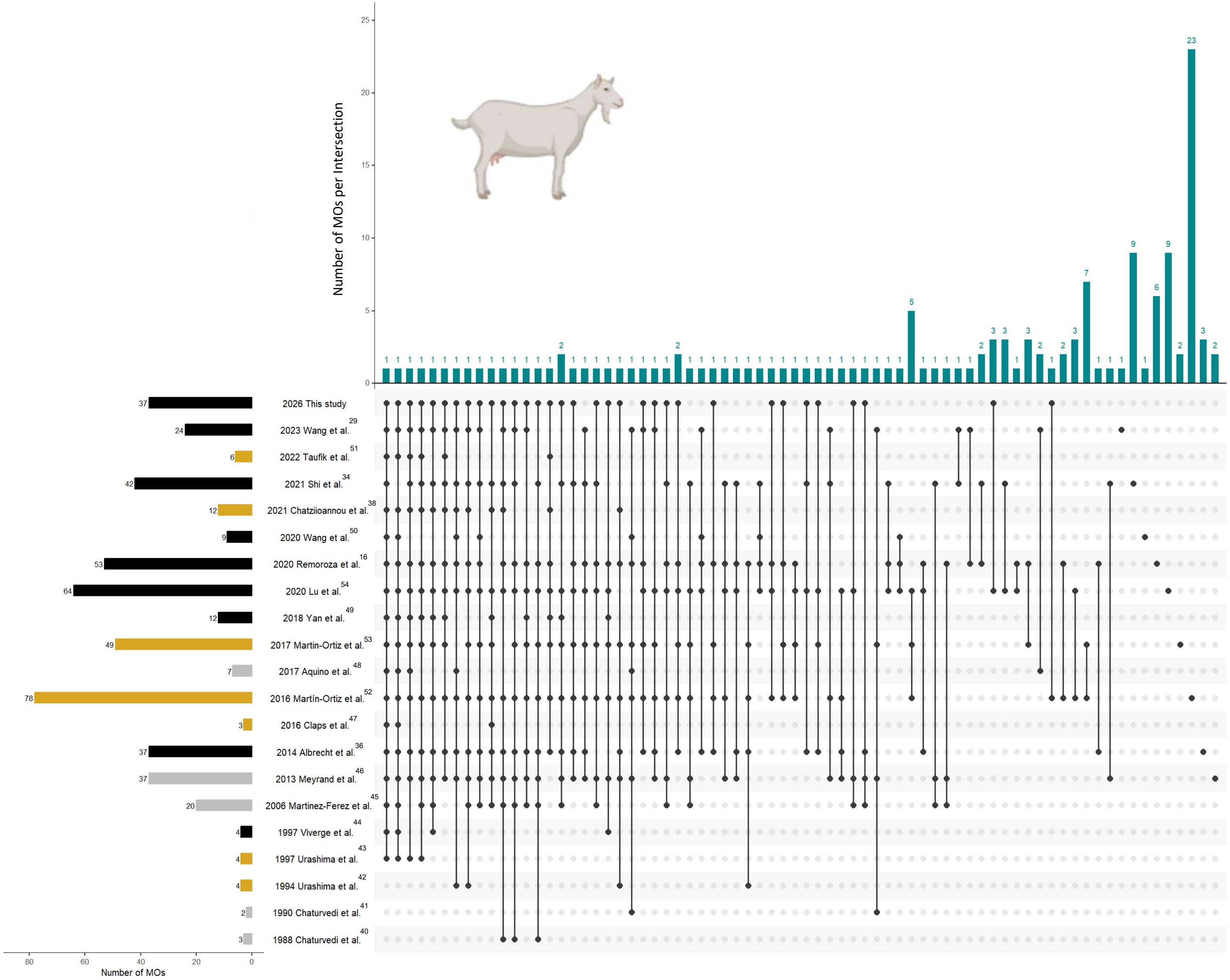

Number of MOs per Intersection
25
20
15
10
5
0
2026 This study
2023 Wang et al.[29]
2022 Taufik et al.[51]
2021 Shi et al.[34]
2021 Chatziioannou et al.[38]
2020 Wang et al.[50]
2020 Remoroza et al.[16]
2020 Lu et al.[54]
2018 Yan et al.[49]
2017 Martin-Ortiz et al.[53]
2017 Aquino et al.[48]
2016 Martín-Ortiz et al.[52]
2016 Claps et al.[47]
2014 Albrecht et al.[36]
2013 Meyrand et al.[46]
2006 Martinez-Ferez et al.[45]
1997 Viverge et al.[44]
1997 Urashima et al.[43]
1994 Urashima et al.[42]
1990 Chaturvedi et al.[41]
1988 Chaturvedi et al.[40]
37
24
6
42
12
9
53
64
12
49
7
78
3
37
37
20
4
4
4
2
3
80
60
40
20
0
Number of MOs

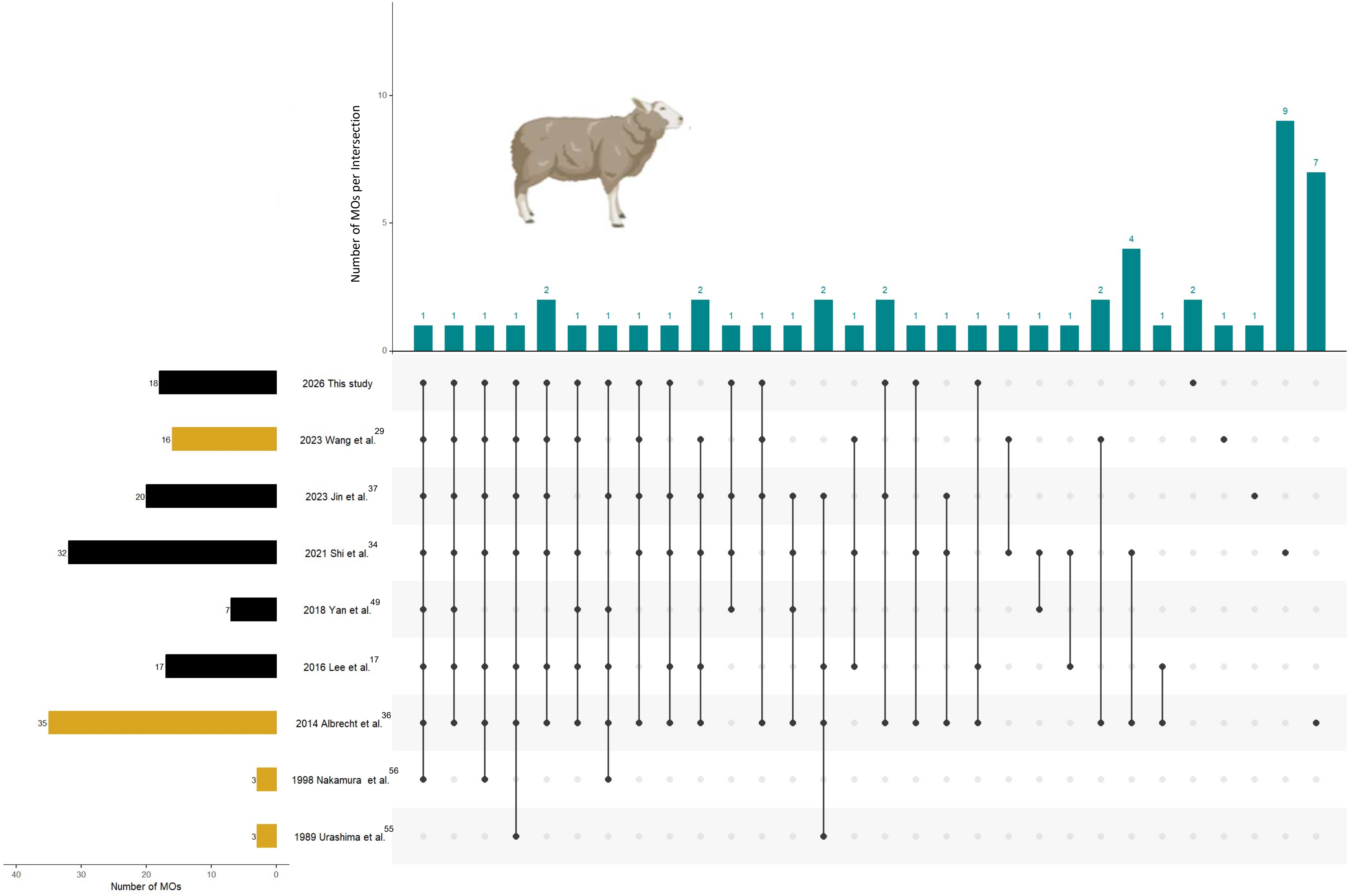
Number of MOs per Intersection
10
5
0
1
2
4
9
7
2026 This study
2023 Wang et al.[29]
2023 Jin et al.[37]
2021 Shi et al.[34]
2018 Yan et al.[49]
2016 Lee et al.[17]
2014 Albrecht et al.[36]
1998 Nakamura et al.[56]
1989 Urashima et al.[55]
18
16
20
32
7
17
35
3
3
40
30
20
10
0
Number of MOs
