# Supplementary material for: Analysis of the Milk Oligosaccharides Spectrum and Sialylation Status of Milk from West African Dwarf Goat and East Friesian Sheep
Source: ACS Omega. 2026 May 27;11(22):32310–20. doi: 10.1021/acsomega.5c13396 (PMC13261409; doi:10.1021/acsomega.5c13396)
Supplement: Supplementary file 4 [file ao5c13396_si_004.pdf]

Analysis of the Milk Oligosaccharides Spectrum and Sialylation Status of Milk from West African Dwarf Goat and East Friesian Sheep

Lisa Isernhagen[a], Christina E. Galuska[a], Andreas Hoeflich[a] and Sebastian P. Galuska[a, *]

[a]: Research Institute for Farm Animal Biology (FBN), Wilhelm-Stahl-Allee 2, 18196 Dummerstorf, Germany; [*]: Corresponding author: Sebastian P. Galuska, galuska.sebastian@fbn-dummerstorf.de

Table S1: Literature comparison for goat and sheep MOs. Given is the last name of the first author, the year, the breed and country of origin for the samples, as well as the time category (colostrum or mature milk), whether the samples were analyzed pooled or individually, the methodology and derivatization, as well as the number of MOs described in the categories neutral, non-fucosylated; neutral, fucosylated; Neu5Ac-sialylated; Neu5Gc-sialylated; Neu5Ac-Neu5Gc-sialylated and total MOs.

Sheep

First Author	Year	Breed	Country of Origin	Time Category	Individual / Pooled	Methodology	Derivatization	Neutral, non-fucosylated	Neutral, fucosylated	Neu5Ac-sialylated	Neu5Gc-sialylated	Neu5Ac-Neu5Gc-sialylated	total MOs
Urashima	1989	Unknown	Japan	Colostrum	Pooled	13C-NMR	Permethylation	3	0	0	0	0	3
Nakamura	1998	Unknown	Japan	Colostrum	Pooled	HPLC/1H-NMR	none	0	0	1	4	0	5
Albrecht	2014	Scottish black-faced mountain sheep	Ireland	Colostrum	Pooled	HILIC-MS	2-AB-Labeling	13	3	8	10	1	35
Lee	2016	Unknown	New Zealand	Mature milk	Unknown	nanoHPLC-Chip/Q-ToF MS	Reduction	8	1	3	5	0	17
Yan	2018	Unknown	China	Mature milk	Unknown	HILIC-MS	none	0	0	1	6	0	7
Shi	2021	Unknown	China	Mature milk	Pooled	MALDI-TOF/TOF MS	Permethylation	11	3	6	9	3	32
Jin	2023	Unknown	Sweden	Mature milk	Unknown	LC-MS/MS	Reduction	8	1	4	7	0	20
Wang	2023	East Friesian and Hu and East Friesian-Hu crossbreed	China	Time-Line	Pooled	UHPLC-ESI-MS/ MS	none	7	2	3	4	0	16
Isernhagen	2025	East Friesian	Germany	Mature milk	Individual	HILIC-MS	none	4	2	4	9	0	19

Goat

First Author	Year	Breed	Country of Animal	Time Category	Individual / Pooled	Methodology	Derivatization	Neutral, non-fucosylated	Neutral, fucosylated	Neu5Ac-sialylated	Neu5Gc-sialylated	Neu5Ac-Neu5Gc-sialylated	total MOs
Chaturvedi	1988	Unknown	India	Unknown	Unknown	1H-NMR	none	3	0	0	0	0	3
Chaturvedi	1990	Unknown	India	Unknown	Unknown	1H-NMR	none	0	2	0	0	0	2
Urashima	1994	Japanese Saanen	Japan	Colostrum	Unknown	1H- / 13C-NMR	none	3	1	0	0	0	4
Urashima	1997	Japanese Saanen	Japan	Colostrum	Unknown	1H-NMR	none	0	0	3	1	0	4
Viverge	1997	Unknown	Unknown	Mature milk	Unknown	1H-NMR	none	0	0	4	0	0	4
Martinez-Ferez	2006	Unknown	Spain	Unknown	Individual	HPAEC-PAD / FAB-MS	none	7	0	7	4	2	20
Meyrand	2013	Alpine	Unknown	Unknown	Individual	Nano-LC-Chip–Q-TOF MS	none	14	6	10	5	2	37
Albrecht	2014	Unknown	Ireland	Mature milk	Pooled	HILIC-MS	2-AB-Labeling	13	3	7	13	1	37
Claps	2016	Garganica and Saanen	Italy	Time-Line	Individual	HPAEC-PAD	none	0	0	3	0	0	3
Martín-Ortiz	2016	Murciano-Granadina	Spain	Colostrum	Individual and pooled	Nano-LC-Chip-Q-TOF MS / HILIC-MS	none	40	3	18	13	4	78
Aquino	2017	Unknown	United States of America	Unknown	Pooled	HPAEC-PAD	none	0	4	3	0	0	7
Martín-Ortiz	2017	Murciano-Granadina	Spain	Time-Line	Individual and pooled	HILIC-QMS / Nano-LC-Chip-Q-TOF MS	none	23	4	11	9	2	49
Yan	2018	Unknown	China	Mature milk	Unknown	HILIC-MS	none	0	0	7	4	1	12
Lu	2020	Guanzhong and Saanen	China	Mature milk	Individual	UPLC-Q-Exactive Focus-MS	none	22	5	19	14	4	64
Remoroza	2020	Saanen	Philippines	Mature milk	Unknown	HILIC-MS/MS	none	25	6	16	6	0	53
Wang	2020	Unknown	China	Mature milk	Unknown	HPAEC-PAD	none	1	3	5	0	0	9
Chatziioannou	2021	Dutch Saanen (Melkgeit)	Netherlands	Time-Line	Individual and pooled	HPLC-FLD	2-AB-Labeling	3	1	5	3	0	12
Shi	2021	Saanen	China	Mature milk	Pooled	MALDI-TOF/TOF MS	Permethylation	18	7	7	8	2	42
Taufik	2022	Etawah Grade	Indonesia	Colostrum	Pooled	HPLC / 1H-NMR	none	0	0	3	3	0	6
Wang	2023	Saanen	China	Mature milk	Pooled	UHPLC-ESI-MS/ MS	none	6	9	6	3	0	24
Isernhagen	2025	West African Dwarf Goats	Germany	Mature milk	Individual	HILIC-MS/MS	none	11	2	13	9	2	37
