# Supplementary material for: Analysis of the Milk Oligosaccharides Spectrum and Sialylation Status of Milk from West African Dwarf Goat and East Friesian Sheep
Source: ACS Omega. 2026 May 27;11(22):32310–20. doi: 10.1021/acsomega.5c13396 (PMC13261409; doi:10.1021/acsomega.5c13396)
Supplement: Supplementary file 5 [file ao5c13396_si_005.pdf]

Analysis of the Milk Oligosaccharides Spectrum and Sialylation Status of Milk from West African Dwarf Goat and East Friesian Sheep

Lisa Isernhagen[a], Christina E. Galuska[a], Andreas Hoeflich[a] and Sebastian P. Galuska[a, *]

[a]: Research Institute for Farm Animal Biology (FBN), Wilhelm-Stahl-Allee 2, 18196 Dummerstorf, Germany; [*]: Corresponding author: Sebastian P. Galuska, galuska.sebastian@fbn-dummerstorf.de

Table S2: Sialic acid quantification. The results for the Neu5Ac and Neu5Gc quantification in West African dwarf goat milk (n=3) and East Friesian sheep milk (n=3) is given with individual ng/µl values as well as the average calculation for each species including the standard error. Milk_Neu5Ac: Neu5Ac quantitated in whole milk; milk_Neu5Gc: Neu5Gc quantitated in whole milk; milk_SA: sum of milk_Neu5Ac and milk_Neu5Gc; MOs_Neu5Ac: Neu5Ac quantitated in the MO fraction; MOs_Neu5Gc: Neu5Gc quantitated in the MO fraction; MOs_SA: sum of MOs_Neu5Ac and MOs_Neu5Gc.

Species	Animal	ID	milk_Neu5Ac [ng/µl]	milk_Neu5Gc [ng/µl]	milk_SA (Neu5Ac + Neu5Gc) [ng/µl]	MOs_Neu5Ac [ng/µl]	MOs_Neu5Gc [ng/µl]	MOs_SA (Neu5Ac + Neu5Gc) [ng/µl]
goat	1	goat_1	150,10	102,77	252,87	40,67	31,07	71,74
goat	2	goat_2	128,69	84,18	212,87	33,28	27,92	61,20
goat	3	goat_3	113,42	55,00	168,42	42,29	22,85	65,14
sheep	1	sheep_1	11,34	166,17	177,51	1,85	49,70	51,55
sheep	2	sheep_2	12,07	147,39	159,46	2,69	58,44	61,13
sheep	3	sheep_3	20,28	170,35	190,63	3,28	66,18	69,46

Average	milk_Neu5Ac [ng/µl]	milk_Neu5Gc [ng/µl]	milk_SA (Neu5Ac + Neu5Gc) [ng/µl]	MOs_Neu5Ac [ng/µl]	MOs_Neu5Gc [ng/µl]	MOs_SA (Neu5Ac + Neu5Gc) [ng/µl]
goat	130.74 ± 10.64	80.65 ± 13.9	211.39 ± 24.39	38.74 ± 2.77	27.28 ± 2.4	66.03 ± 3.08
sheep	14.56 ± 2.87	161.31 ± 7.06	175.87 ± 9.04	2.61 ± 0.42	58.11 ± 4.76	60.72 ± 5.17
